# Exploiting O-GlcNAc dyshomeostasis to screen O-GlcNAc transferase intellectual disability variants

**DOI:** 10.1016/j.stemcr.2024.11.010

**Published:** 2024-12-19

**Authors:** Huijie Yuan, Conor W. Mitchell, Andrew T. Ferenbach, Maria Teresa Bonati, Agnese Feresin, Paul J. Benke, Queenie K.G. Tan, Daan M.F. van Aalten

**Affiliations:** 1 Section for Neurobiology, Department of Molecular Biology and Genetics, Aarhus University, Aarhus, Denmark; 2 Danish Research Institute of Translational Neuroscience DANDRITE-Nordic EMBL Partnership for Molecular Medicine, Aarhus University, Aarhus, Denmark; 3 Division of Molecular, Cell and Developmental Biology, School of Life Sciences, University of Dundee, Dundee, UK; 4 Institute for Maternal and Child Health IRCCS Burlo Garofolo, Trieste, Italy; 5 Department of Medicine, Surgery and Health Sciences, University of Trieste, Trieste, Italy; 6 Joe DiMaggio Children’s Hospital, Hollywood, FL, USA; 7 Department of Clinical Genomics, Mayo Clinic, Rochester, NY, USA

**Keywords:** OGT, O-GlcNAc, neurodevelopment, OGT-CDG

## Abstract

O-GlcNAcylation is an essential protein modification catalyzed by O-GlcNAc transferase (OGT). Missense variants in OGT are linked to a novel intellectual disability syndrome known as OGT congenital disorder of glycosylation (OGT-CDG). The mechanisms by which OGT missense variants lead to this heterogeneous syndrome are not understood, and no unified method exists for dissecting pathogenic from non-pathogenic variants. Here, we develop a double-fluorescence strategy in mouse embryonic stem cells to measure disruption of O-GlcNAc homeostasis by quantifying the effects of variants on endogenous OGT expression. OGT-CDG variants generally elicited a lower feedback response than wild-type and Genome Aggregation Database (gnomAD) OGT variants. This approach was then used to dissect new putative OGT-CDG variants from pathogenic background variants in other disease-associated genes. Our work enables the prediction of pathogenicity for rapidly emerging *de novo* OGT-CDG variants and points to reduced disruption of O-GlcNAc homeostasis as a common mechanism underpinning OGT-CDG.

## Highlights

• The creation of a fluorescently labeled OGT-sfGFP mouse embryonic stem cell line
• The first high-throughput method for OGT-CDG variant prediction
• Reduced O-GlcNAc dyshomeostasis is the common feature for OGT-CDG variants

## Graphical abstract

**Figure:**
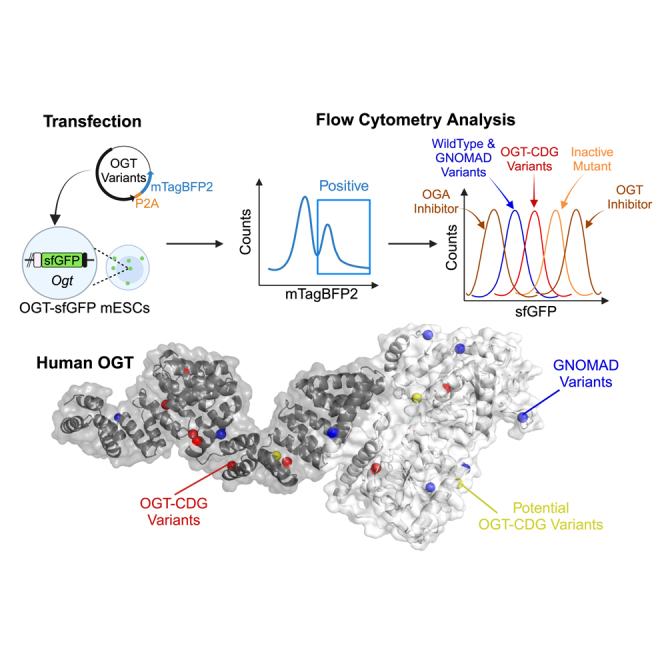

## Teaser

van Aalten et al. developed an endogenous knockin reporter system where the fluorescence signal from tagged OGT serves as a convenient readout to predict the pathogenicity of ID-associated OGT variants. This approach represents the first high-throughput method for OGT-CDG variant screening and highlights that reduced disruption of O-GlcNAc homeostasis is a common feature among OGT-CDG variants.

## Introduction

O-GlcNAcylation, the modification of Ser/Thr hydroxyls with N-acetylglucosamine, is a widespread and conserved post-translational modification (PTM) occurring on over 9,000 nuclear, cytoplasmic, and mitochondrial proteins (Wulff-Fuentes et al., 2021). Unlike other PTMs, O-GlcNAcylation is regulated by just two enzymes. The O-GlcNAc transferase, OGT, composed of an N-terminal tetratricopeptide repeat (TPR) domain and a C-terminal glycosyltransferase (GT) domain, modifies Ser/Thr hydroxyls with GlcNAc (Haltiwanger et al., 1992) while the antagonistic enzyme, the O-GlcNAc hydrolase (OGA), removes O-GlcNAc (Dong and Hart, 1994). The OGT TPR domain forms a superhelical structure that mediates substrate binding and selectivity (Joiner et al., 2021).

Recently, missense variants in OGT have been reported in patients with a novel, syndromic form of X-linked intellectual disability (ID), called the OGT-linked congenital disorder of glycosylation (OGT-CDG) (Pravata et al., 2020a). OGT-CDG variants are present in the TPR and GT domains and lead to a wide range of facial dysmorphic, neurological, behavioral, and peripheral abnormalities. Multiple hypotheses have been proposed to explain how OGT-CDG variants affect OGT activity to cause disease, and various model systems including patient-derived lymphoblasts and skin fibroblasts, human and mouse embryonic stem cells (h/mESCs), *Drosophila melanogaster* and knockin mice have been generated to dissect the effects of OGT-CDG variants on OGT activity and identify dysregulated pathways (Authier et al., 2024; Omelková et al., 2023; Pravata et al., 2019; 2020b; Selvan et al., 2018; Vaidyanathan et al., 2017; Willems et al., 2017). These model systems, in combination with *in vitro* and structural characterization of OGT-CDG variants, have identified destabilizing effects of some TPR variants (Gundogdu et al., 2018; Vaidyanathan et al., 2017; Willems et al., 2017) and loss of catalytic activity of GT domain variants (Omelková et al., 2023; Pravata et al., 2019; 2020b), suggesting that a mixture of dosage and catalytic effects may contribute to disease etiology. However, differences in the dependencies of different cell lines and model organisms for O-GlcNAc and cell-type-specific regulatory mechanisms for the O-GlcNAc cycling enzymes, have complicated the identification of common biochemical characteristics/phenotypes between OGT-CDG variants. Additionally, the different genetic backgrounds of cell and model organisms may interact with the effects of a given OGT-CDG variant, calling into question the generality of any observed effects on O-GlcNAc homeostasis, cell signaling, or organism development. A salient example of this is the OGT A319T variant, which is present in a patient alongside a missense variant in the ID-associated gene *MED12* (Bouazzi et al., 2015). Consequently, no rapid screening method, exploiting a single isogenic background, is available for OGT-CDG, and putative disease variants identified by clinicians must be characterized *in vitro* and *in cellulo*, a costly and time-consuming process that delays the identification of *bona fide* OGT-CDG variants.

Here, we report the generation of an isogenic fluorescent reporter system for measuring the effects of OGT variants on O-GlcNAc homeostasis. Building on the previously described feedback mechanism linking elevated O-GlcNAc levels to reduced endogenous OGT protein levels (Slawson et al., 2005), we fluorescently tagged OGT in mESCs to assay the effects of transfected OGT variants on endogenous OGT levels as a readout of O-GlcNAc homeostasis. Flow cytometry (FC) revealed that while exogenous wild-type OGT markedly reduces endogenous OGT expression, a catalytically inactive OGT mutant does not. Non-pathogenic OGT variants from the general population (Genome Aggregation Database [gnomAD] variants) influence O-GlcNAc homeostasis similarly to wild-type OGT, whereas most OGT-CDG variants differ, showing limited capacity to disrupt O-GlcNAc homeostasis. We report three new OGT-CDG variants and dissect these from background variants in other disease genes, underscoring the potential pathogenic role of a segregating missense mutation in *LMNA*. Collectively, our findings suggest that a reduced ability to disrupt O-GlcNAc homeostasis is a feature that can be used to screen OGT-CDG variants.

## Results

### Three unrelated patients affected by unclassified ID segregate with OGT missense variants

Proband 1 was the firstborn male of healthy, unrelated Caucasian parents with two younger sisters. He was born at 41 weeks via C-section due to breech presentation, at a weight of 2,680 g (3^rd^ centile, small for gestational age), length of 48 cm (7^th^ centile), and OCF of 33 cm (7^th^ centile). Apgar scores were 5, 8, and 10 at 1, 5, and 10 min, respectively. Pregnancy complications included an echogenic intracardiac focus and an arachnoid cyst, with no invasive procedures performed. At birth, the proband had cyanosis due to bilateral asymmetric choanal stenosis, confirmed by maxillofacial computed tomography. Neonatal hypotonia and recurrent bronchiolitis were observed, along with patent foramen ovale and ductus arteriosus on echocardiogram. He had congenital VI cranial nerve paralysis leading to monolateral strabismus and hypermetropia, corrected by lenses. Developmentally, he experienced a global delay, sitting at 9 months, walking at 19 months, and delayed speech progression. At 3.5 years, he formed simple sentences with phonological issues, echolalia, and comprehension difficulties. WPPSI-III testing at 3 years and 4 months indicated borderline cognitive abilities (intelligence quotient 79). Motor skills, particularly manual dexterity and balance (M-ABC-2), were below average. MRI revealed no brain abnormalities. At 6 years, the proband showed poor gaze, sociability, and attention difficulties. Dysmorphic features included heavy eyebrows, narrow palpebral fissures, smooth philtrum, full lips, high-arched palate, clinodactyly, toe syndactyly, and a hyperpigmented dorsal spot. His height was 111 cm (13^th^ centile) and COF 49.7 cm (8^th^ centile), with a stocky build. Genetic testing revealed a wild-type FMR1 CGG expansion and trio-SNP array. Trio-WES identified the *OGT* (GenBank: NM_181672.3): c.1004A>G, p.(N335S) variant, inherited from his asymptomatic mother and maternal grandmother. His mother showed an unbalanced X chromosome inactivation pattern (93:07), while the grandmother’s analysis was uninformative.

Proband 2 was a female child of healthy, unrelated parents of Eastern European ancestry, with an older sister and younger brother. She was born at 40 weeks via vaginal delivery after a pregnancy conceived via *in vitro* fertilization, complicated by suspected aortic coarctation, though postnatal echocardiogram was normal. Her birth weight was 3.1 kg (27^th^ percentile), and length was 51 cm (75^th^ percentile). Family history was notable for a paternal uncle with schizophrenia, with no other neurodevelopmental disorders. In infancy, the proband exhibited clenched fists and delayed fine motor skills, particularly with her pincer grasp at 9 months. She met early motor milestones, sitting at 6 months and standing at 10 months, but walked independently only at 21 months. Speech development stalled after her first word at 12 months, with regression in both speech and motor skills, increased drooling, and a return to soft foods. Electroencephalogram results were normal. Developmental testing at 33 months revealed motor skills equivalent to an 18–20 month old, with a developmental quotient of 79 (Mullen Scales of Early Learning). She did not meet the criteria for autism spectrum disorder. At almost 4 years, she babbled, used 4–30 word approximations, and signed 2–3 words. She walked with inverted feet and ran but often fell, scribbled, and used utensils, though her cognitive processing was slow. She displayed sociable behavior, being overly friendly with strangers, and had occasional tantrums. Intermittent right eye esotropia was noted since 1 year of age, along with adenoid hypertrophy and frequent strep infections. Genetic testing via trio-WES identified a *de novo OGT* variant (c.2542A>T, p.(N848Y)) and a *de novo LMTK3* variant (c.1391G>A, p.(W464^∗^)). At 3 years and 6 months, her height was 88 cm (8^th^ percentile), weight 12.2 kg (13^th^ percentile), and head circumference 46 cm (2^nd^ percentile). Physical examination at 46 months revealed epicanthal folds, prominent infra-orbital folds, anteverted nares, hypotonia, brisk symmetric patellar reflexes, and downgoing Babinski toes, with no digit anomalies.

Proband 3 was a male evaluated at age 9 for cardiomyopathy present since birth and autism features that emerged between ages 2 and 3. He experienced failure to thrive until age 4, requiring breastfeeding and later a pureed food diet. Speech development was delayed, beginning at age 4. He exhibited hand flapping, inconsistent eye contact, and frequent tantrums, leading to an autism diagnosis. Genetic testing revealed two variants of uncertain significance (VUSs): *LMNA* c.1634 G>A, p.(R545H), initially linked to cardiomyopathy but later reclassified as non-pathogenic, and *OGT* c.3040 A>G, p.(M1014V). At age 9, his height was at the 18^th^ percentile and weight at the 3^rd^ percentile. He presented with a mild prominent forehead, flexible joints, a cardiac murmur, decreased muscle mass, fair eye contact, and normal speech and comprehension. Cardiac findings were stable, and speech had significantly improved. He performed at grade level with mild delays in reading and math. While hyperactivity persisted, his autism features had greatly improved, and attention-deficit/hyperactivity disorder became his primary neurodevelopmental diagnosis.

Patients’ clinical phenotypes are summarized in Table S1.

### Fusion of sfGFP to endogenous OGT does not disrupt O-GlcNAc homeostasis

**Figure 1:**
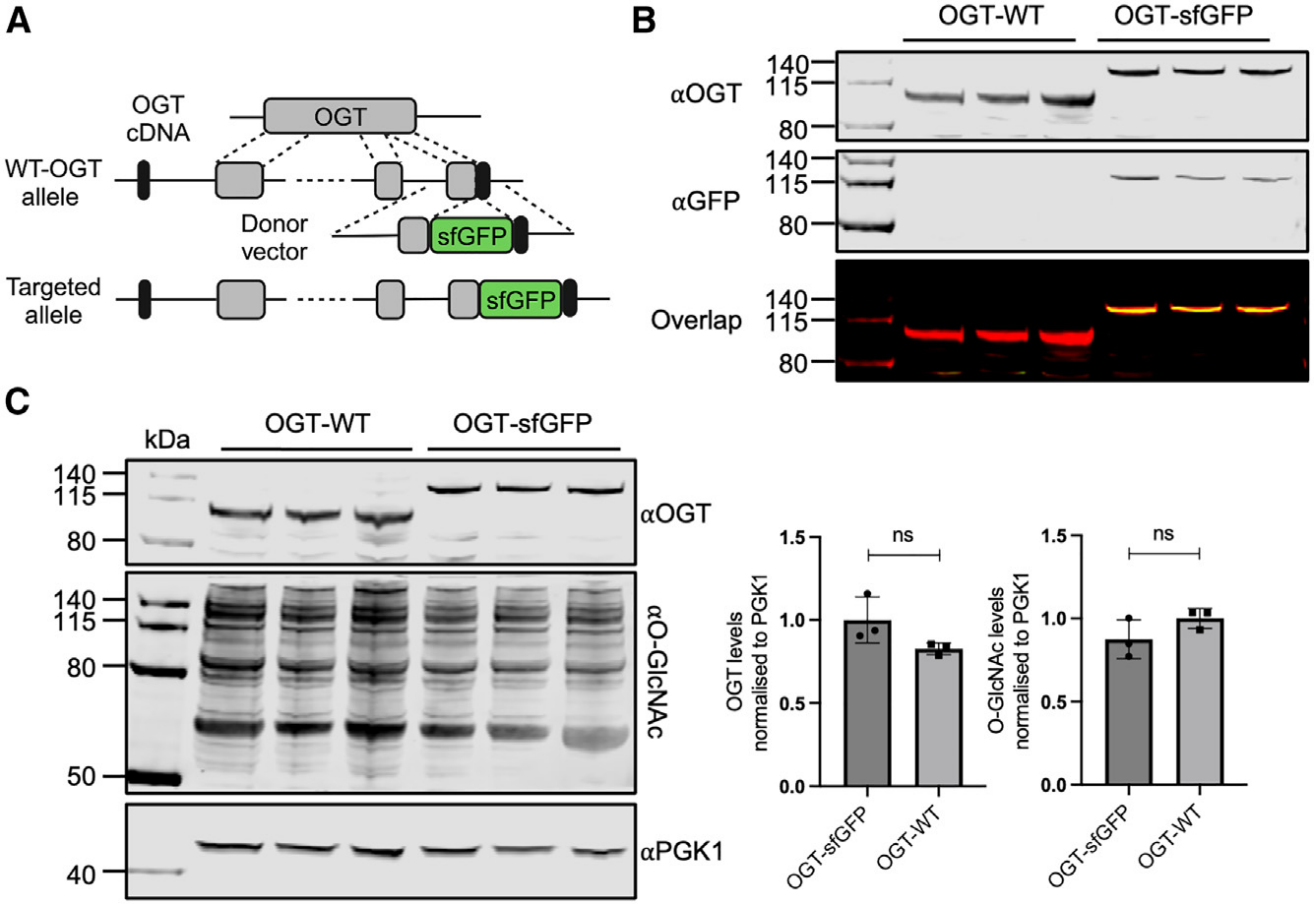
Fusion of sfGFP to endogenous OGT does not disrupt O-GlcNAc homeostasis (A) Schematic of the CRISPR knockin strategy used to fuse sfGFP to the C terminus of endogenous *Ogt* in mESCs. (B) Immunoblot of proteins extracted from CRISPR-engineered OGT-sfGFP and untreated wild-type mESCs, using antibodies against OGT and GFP (see also Figure S1). (C) Left: OGT and O-GlcNAc (RL2) levels in OGT-sfGFP mESCs compared to untreated wild-type mESCs, with PGK1 as a loading control. Right: O-GlcNAc and OGT levels normalized to PGK1 (*n* = 3 independent experiments). Error bars represent the standard error of the mean (SEM). *p* values (unpaired t test): OGT = 0.10, O-GlcNAc = 0.17.

Novel OGT missense variants segregating with ID are typically classified as VUS. Traditional classification requires extensive lab work, and we sought to establish a rapid, high-throughput screening method to identify pathogenic OGT-CDG variants. Given the feedback regulation between OGT, OGA, and global O-GlcNAc levels (Decourcelle et al., 2020), we hypothesized that monitoring OGT levels would offer a reliable readout of O-GlcNAc dyshomeostasis induced by OGT-CDG variants. The super folder variant of green fluorescent protein (sfGFP) (Pédelacq et al., 2006) was fused to the C terminus of endogenous *Ogt* in male mESCs using CRISPR genome editing (Figure 1A). Immunoblotting for OGT showed the expected molecular weight shift of OGT-sfGFP in edited mESCs compared to untreated wild-type controls, and immunoblotting for GFP revealed overlapping signals for OGT and GFP in OGT-sfGFP cells (Figure 1B). No significant changes in O-GlcNAc or OGT levels were observed in the edited OGT-sfGFP mESCs (Figure 1C). Interestingly, only the full-length OGT isoform was observed in these cells (Figure S1). Taken together, these data indicate that sfGFP fusion to endogenous OGT does not disrupt O-GlcNAc homeostasis.

### OGT-sfGFP fluorescence responds to the pharmacological disruption of O-GlcNAc homeostasis

**Figure 2:**
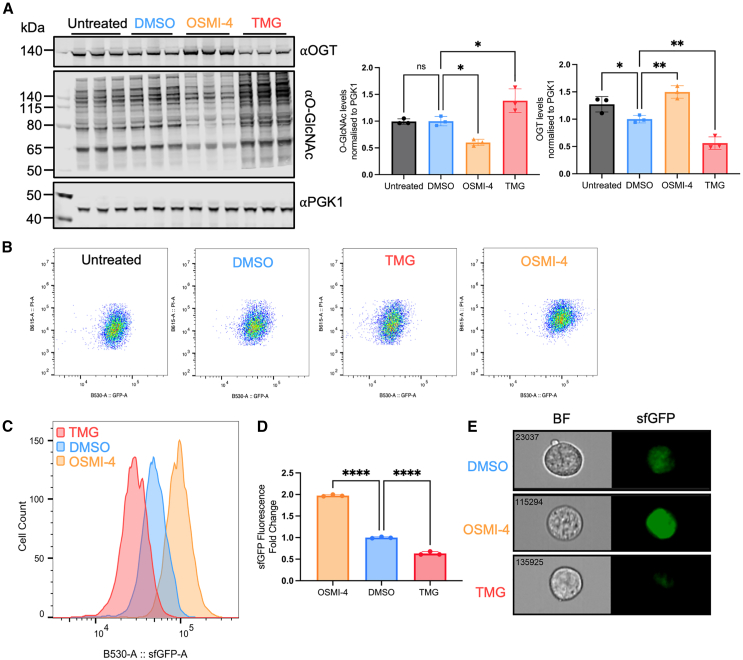
OGT-sfGFP levels/fluorescence respond to the pharmacological disruption of O-GlcNAc homeostasis (A) Immunoblot showing OGT and O-GlcNAc (RL2) levels in OGT-sfGFP mESCs after 24-h treatment with 10 μM OSMI-4b or TMG, alongside a 0.1% DMSO vehicle control and an untreated control. PGK1 served as a loading control. Statistical analysis was performed on the right using urdinary one-way ANOVA (*n* = 3 independent replicates), with error bars representing mean ± SEM. (B) Density plots of gated live singlet OGT-sfGFP mESCs after 10 μM OSMI-4b or TMG treatments (see gating strategy in the no-transfection section of Figure S2). OGT-sfGFP fluorescence is displayed on the x axis, and propidium iodide (PI) fluorescence on the y axis. (C) Overlay of sfGFP histograms showing gated live singlet OGT-sfGFP mESCs after 10 μM OSMI-4b or TMG treatments, with OGT-sfGFP fluorescence on the x axis and cell count on the y axis. (D) Median sfGFP fluorescence values from the sfGFP histogram (Figure 2C) were extracted for each sample and subjected to statistical analysis via ordinary one-way ANOVA (*n* = 3 independent replicates), with error bars indicating mean ± SEM. (E) Images of OGT-sfGFP mESCs after 10 μM OSMI-4b or TMG treatments, captured in both bright-field (BF) and sfGFP fluorescence channels using an ImageStream flow cytometer. Statistical significance for all experiments is denoted as follows: ^∗^ for adjusted *p* < 0.05, ^∗∗^ for *p* < 0.01, ^∗∗∗∗^ for *p* < 0.0001, and ns for *p* > 0.05.

To assess whether OGT levels in OGT-sfGFP mESCs provide a readout of O-GlcNAc homeostasis, we modulated O-GlcNAc levels using inhibitors of OGA (10 μM Thiamet G [TMG] [Yuzwa et al., 2008]) or OGT (10 μM OSMI-4b [Martin et al., 2018]). As expected, immunoblotting revealed that OSMI-4b treatment decreased O-GlcNAc levels but increased OGT-sfGFP protein levels, whereas TMG treatment increased O-GlcNAc levels but reduced OGT-sfGFP protein levels (Figure 2A). Concomitantly, FC analysis revealed that treatment with OSMI-4b increased sfGFP fluorescence, whereas TMG treatment resulted in decreased fluorescence (Figure 2B, gating strategy provided in the no-transfection section in Figure S2). Clear separations in cell populations were observed in the sfGFP histogram (Figure 2C), showing nearly a 2-fold increase in median sfGFP fluorescence with OSMI-4b treatment and an approximately two-fifths reduction after TMG treatment (Figure 2D). Single-cell images captured during FC corroborate these changes (Figure 2E). FC enables high-throughput analysis of OGT-sfGFP levels with larger sample sizes and decreased variability across different cell passages compared to immunoblotting (Figures 2D and 2A). Collectively, these data show that OGT-sfGFP fluorescence responds to the pharmacological disruption of O-GlcNAc homeostasis, consistent with the established feedback regulation of O-GlcNAc.

### Endogenous OGT-sfGFP is a readout for the activity of exogenous OGT variants

**Figure 3:**
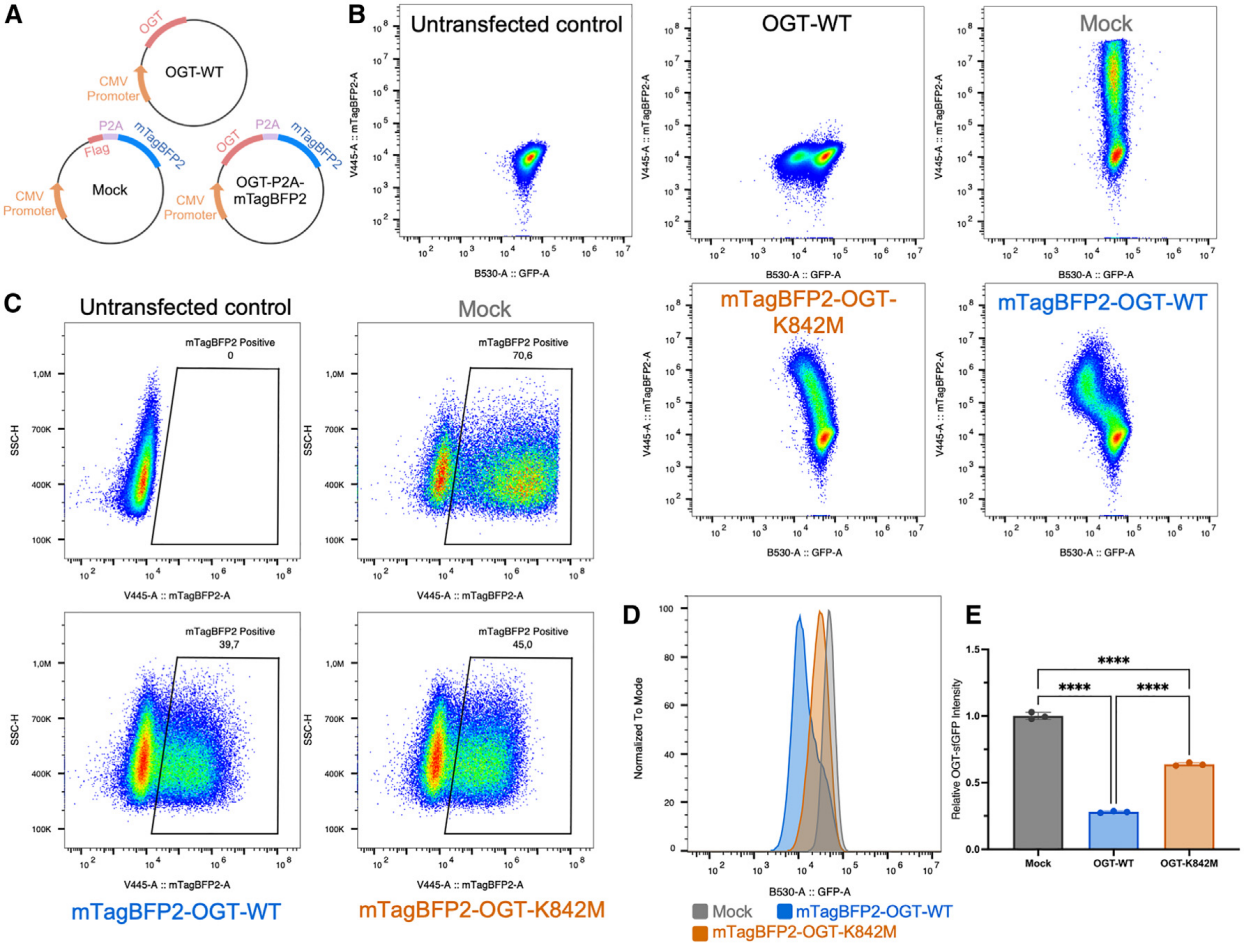
Endogenous OGT-sfGFP is a readout for the activity of exogenous OGT variants (A) Illustration of plasmids used in the study: a wild-type OGT plasmid without fluorescent labeling (OGT-WT), a mock control plasmid with a FLAG tag linked to mTagBFP2 via the P2A linker, and mTagBFP2-OGT plasmids, where OGT or its variants are linked to mTagBFP2 via the P2A linker. (B) Density plots of gated live singlet OGT-sfGFP mESCs after transfection with the unlabeled OGT-WT plasmid, fluorescently labeled mTagBFP2-OGT-K842M or mTagBFP2-OGT-WT plasmids, and the mock control. The x axis shows OGT-sfGFP fluorescence intensity, and the y axis displays mTagBFP2 fluorescence. (C) Selection of transfected mTagBFP2^+^ cells. A universal gate was applied across all samples to select OGT-sfGFP cells expressing mTagBFP2 (see plasmid-transfection section in Figure S2), with untransfected cells used as a control to define the gate. (D) Overlay of sfGFP histograms for gated mTagBFP2^+^ cells. Clear separations in sfGFP fluorescence were observed between cells transfected with mTagBFP2-OGT-WT, mTagBFP2-OGT-K842M, and the mock control plasmids. (E) Statistical analysis of OGT-sfGFP fluorescence in gated mTagBFP2^+^ mESCs following overexpression of mTagBFP2-OGT-WT, mTagBFP2-OGT-K842M, and the mock control. Each data point represents the median sfGFP fluorescence for the corresponding mTagBFP2^+^ cells. Ordinary one-way ANOVA was performed (*n* = 3 independent replicates); ^∗∗∗∗^ denotes an adjusted *p* value <0.0001. Error bars represent mean ± SEM.

Given that endogenous OGT-sfGFP levels were sensitive to pharmacological perturbations of O-GlcNAc levels, we next investigated whether transfection of exogenous OGT variants would affect endogenous OGT-sfGFP fluorescence through O-GlcNAc feedback regulation. To enable gating for transfected cells, we constructed plasmids (Figure 3A) where OGT is fused to fluorescent marker mTagBFP2 (Subach et al., 2011) via a porcine teschovirus-1 self-cleaving 2A peptide (P2A) linker (Kim et al., 2011). The P2A linker enables the translation of separate OGT and mTagBFP2 proteins from a single mRNA strand through ribosome skipping (Kim et al., 2011), generating stochiometric expression of both proteins.

To explore this system, a fluorescently labeled wild-type OGT plasmid (mTagBFP2-OGT-WT) and a catalytically inactive OGT mutant, mTagBFP2-OGT-K842M (Selvan et al., 2015) (Figure 3A), were transfected into OGT-sfGFP mESCs. Wild-type OGT plasmid, lacking the mTagBFP2 fluorescent marker (OGT-WT), and a plasmid encoding only mTagBFP2 (Mock) were used as controls (Figure 3A). OGT-sfGFP mESCs were analyzed by FC 48 h post-transfection. Density maps of gated live cells revealed relationships between sfGFP and mTagBFP2 fluorescence (Figure 3B). Compared to the untransfected control, cells transfected with the unlabeled OGT-WT plasmid showed a subset of cells with reduced OGT-sfGFP fluorescence, indicating downregulation of endogenous OGT-sfGFP following exogenous OGT expression (Figure 3B). The mock plasmid (Figure 3A), encoding only mTagBFP2, exhibited increased mTagBFP2 fluorescence without observable changes in sfGFP fluorescence (Figure 3B), suggesting no disturbance of O-GlcNAc homeostasis. Conversely, mTagBFP2-OGT-WT transfection led to increased mTagBFP2 fluorescence and decreased OGT-sfGFP levels (Figure 3B), further indicating endogenous OGT downregulation in response to exogenous OGT expression. Notably, cells transfected with the catalytic inactive mutant mTagBFP2-OGT-K842M showed only a mild decrease in OGT-sfGFP fluorescence despite high mTagBFP2 levels (Figure 3B), suggesting the feedback regulation may also respond to non-catalytic functions of OGT.

To quantify changes in OGT-sfGFP fluorescence due to exogenous OGT overexpression, a universal gate was employed to select cells with higher mTagBFP2 fluorescence than the untransfected control (Figure 3C; see Figure S2 for gating strategy). These selected mTagBFP2^+^ cells were then presented as an sfGFP histogram (Figure 3D), with median OGT-sfGFP fluorescence used for statistical analysis (Figure 3E), revealing separation of mTagBFP2^+^ cells transfected with mTagBFP2-OGT-WT, mTagBFP2-OGT-K842M, and the mock control (Figure 3D). Cells transfected with mTagBFP2-OGT-WT displayed approximately one-quarter of the OGT-sfGFP fluorescence intensity of the mock control, while mTagBFP2-OGT-K842M-transfected cells retained around three-quarters of the OGT-sfGFP fluorescence intensity (Figure 3E). Taken together, these data demonstrate that endogenous OGT-sfGFP provides a reliable readout for exogenous OGT variant activity and that O-GlcNAc feedback regulation correlates with the activity of the transfected OGT variant.

### Non-pathogenic OGT variants disrupt O-GlcNAc homeostasis similarly to wild-type OGT

**Figure 4:**
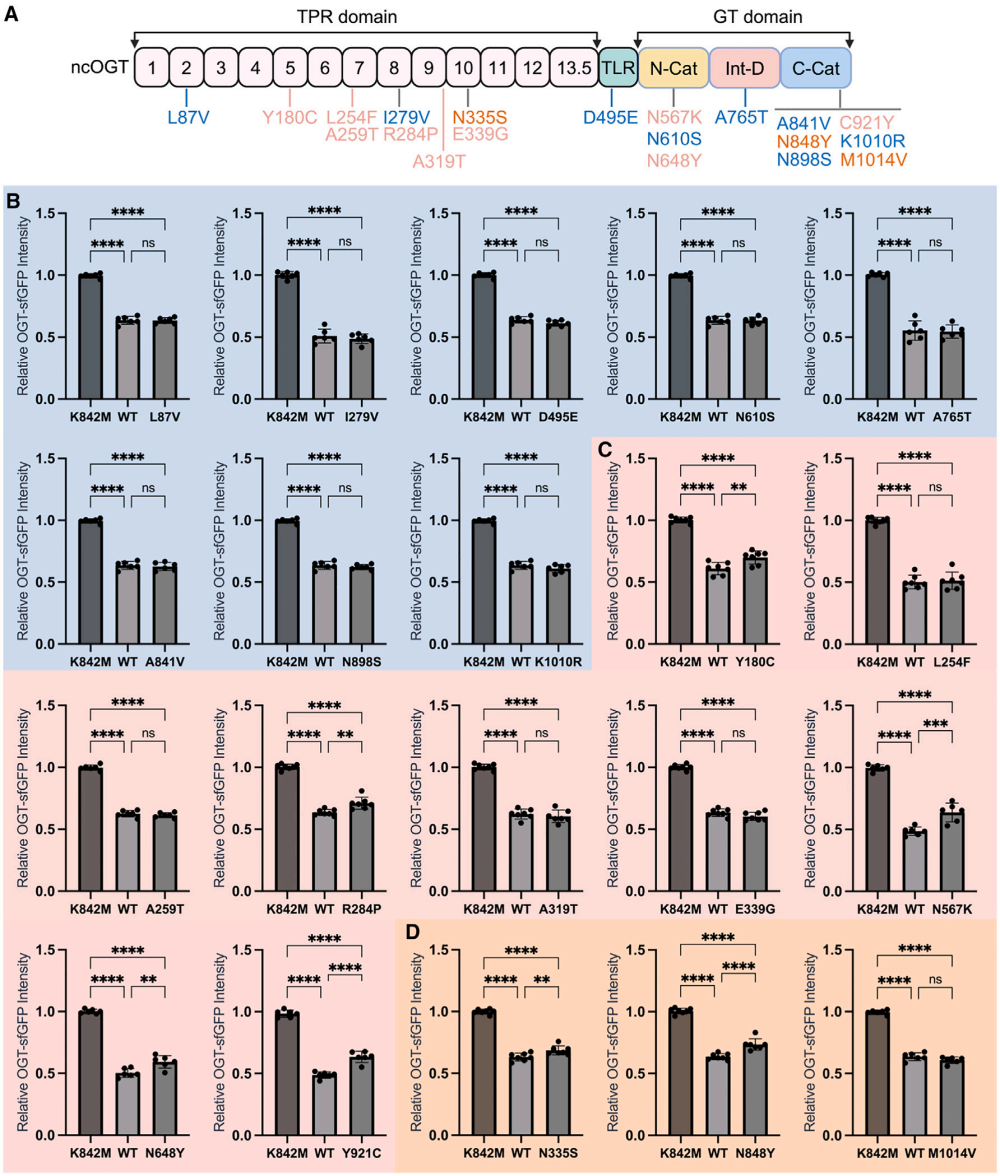
Changes in OGT-sfGFP fluorescence predict OGT-CDG variant pathogenicity (A) Schematic of all OGT variants used in this study. Variants in blue represent the eight most frequent OGT variants from the gnomAD database (gnomAD variants; Table S2). Variants in pink are previously reported pathogenic OGT-CDG variants, while those in orange are three newly identified ID-associated OGT variants reported in this study. All variants are tagged with mTagBFP2 via the P2A linker for transfection. (B–D) Statistical analysis of OGT-sfGFP fluorescence in transfected mTagBFP2^+^ cells. Each OGT variant was transfected into OGT-sfGFP mESCs at least six times across different passages and days. Each data point represents the median OGT-sfGFP fluorescence from an independent transfection sample, normalized to the median fluorescence of the K842M mutant transfected sample. Analysis of gnomAD variants is shown in (B), previously reported pathogenic OGT-CDG variants in (C), and the three newly identified potential OGT-CDG variants in (D). Error bars represent mean ± SEM (*n* ≥ 6). Ordinary one-way ANOVA was performed for each graph, with symbols indicating significance: ^∗∗^ for adjusted *p* < 0.01, ^∗∗∗∗^ for *p* < 0.0001, and ns for *p* > 0.05. A panel of representative density plots for mTagBFP2^+^ cell selection across all transfected variants is presented in Figure S4.

To evaluate the system’s ability to dissecting pathogenic from non-pathogenic OGT variants, we first investigated the eight most common OGT variants in the general population, sourced from gnomAD v.4.0.0 (Chen et al., 2024). These variants span the TPR and GT domains of OGT (Figure 4A), with allele counts ranging from 115 to 21 among 730,947 exome sequences (Table S2). Notably, the original amino acids at these variant sites are generally not evolutionarily conserved, except for Leu279 (Figure S3). Each variant has been observed in hemizygous individuals, with homozygosity documented only for the D495E variant. Given their relative prevalence in the general population, these gnomAD variants are not expected to affect OGT activity. Indeed, when introducing these variants into OGT-sfGFP mESCs, the gated mTagBFP2^+^ cells exhibited reduction in OGT-sfGFP fluorescence comparable to that observed with wild-type OGT transfection (Figure 4B). In all experiments, the catalytically inactive mTagBFP2-OGT-K842M mutant was used as a control to normalize OGT-sfGFP fluorescence in the transfected mTagBFP2^+^ cells. Overall, these findings demonstrate that in our mESC transfection approach, non-pathogenic gnomAD OGT variants similarly disrupt O-GlcNAc homeostasis as wild-type OGT, eliciting O-GlcNAc feedback response to a comparable level.

### Changes in OGT-sfGFP fluorescence predict OGT-CDG variant pathogenicity

The sensitivity of OGT-sfGFP fluorescence to transfected exogenous OGT plasmids suggests that OGT-CDG variants could provoke a less pronounced feedback regulation than non-pathogenic OGT variants. To test this, all currently reported pathogenic OGT-CDG variants were cloned (Omelková et al., 2023; Pravata et al., 2019, 2020b; Selvan et al., 2018; Vaidyanathan et al., 2017; Willems et al., 2017), each labeled with mTagBFP2 via the P2A linker (Figure 4A). These plasmids were transfected into OGT-sfGFP mESCs and analyzed by FC to examine OGT-sfGFP levels in mTagBFP2^+^ cells as described earlier.

Interestingly, all reported pathogenic OGT-CDG variants in the GT domain produced a significantly smaller reduction in OGT-sfGFP fluorescence than wild-type OGT (Figure 4C), suggesting that these variants are either less active or more unstable, leading to weaker disruption of O-GlcNAc homeostasis. Nevertheless, the reduction was still more substantial than the response elicited by the inactive K842M OGT mutant (Figure 4C), which serves as a baseline to normalize OGT-sfGFP fluorescence in each transfection. This aligns with previous research supporting the pathogenic nature of these OGT-CDG variants. Strikingly, certain variants in the TPR domain, including the previously reported R284P OGT-CDG variant (Willems et al., 2017), the newly identified N335S putative OGT-CDG variant, and the Y180C variant (unpublished data), exhibit similar effects to OGT-CDG variants in the catalytic domain, with an attenuated reduction in endogenous OGT-sfGFP fluorescence after transfection (Figures 4C and 4D). However, other OGT-CDG TPR variants (L254F, A259T, A319T, E339G; Figure 4A) behaved similarly to the wild type in OGT-sfGFP mESCs (Figure 4C).

**Figure 5:**
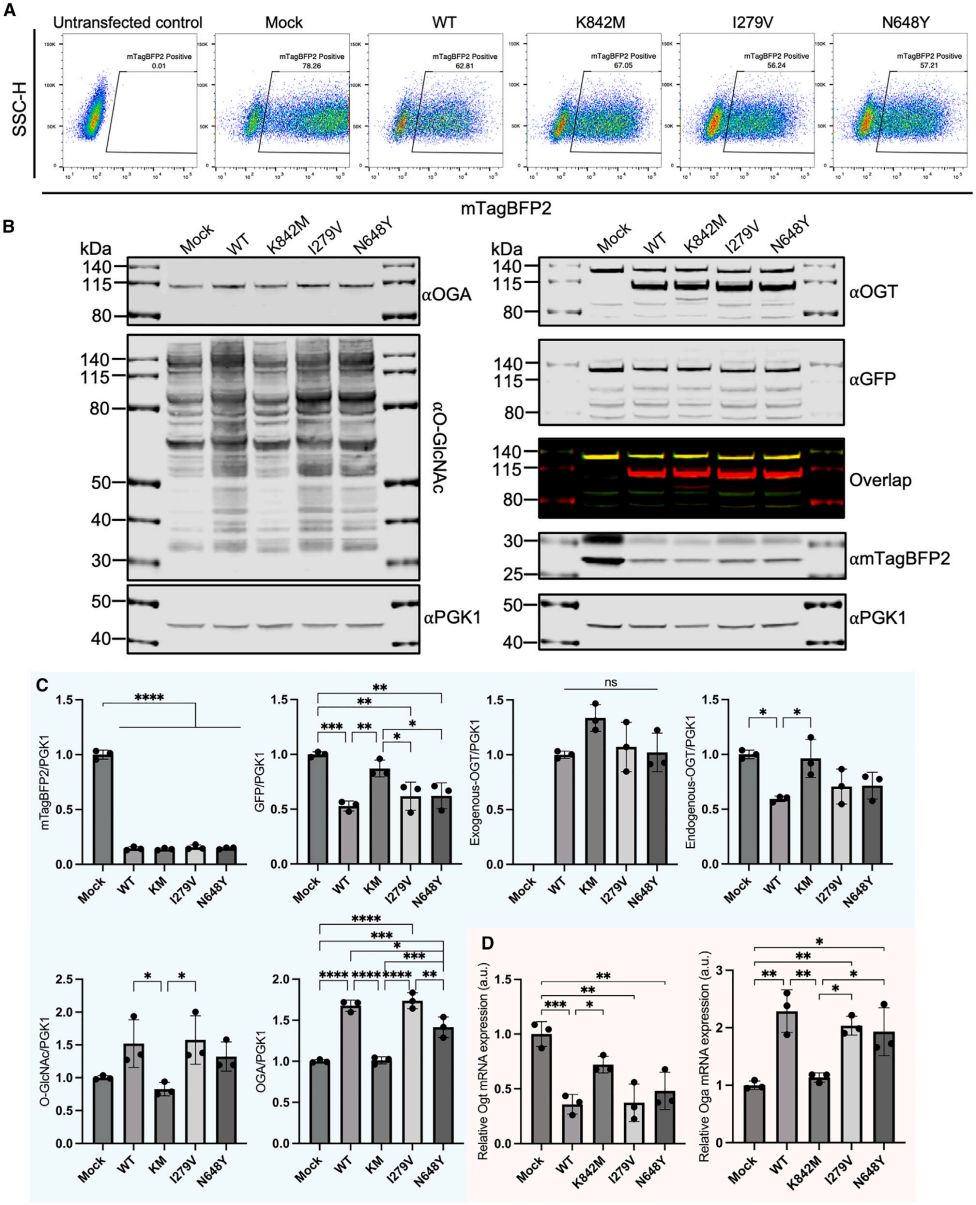
O-GlcNAc feedback regulation involves the transcriptional coordination of OGT and OGA (A) Representative density plots for sorting mTagBFP2^+^ cells following transfection with a subset of plasmids, including the mock control, wild-type OGT, the inactive K842M OGT mutant, the gnomAD I279V variant, and the pathogenic N648Y OGT-CDG variant. The sorted mTagBFP2^+^ cells were then used for immunoblotting (B and C) and RT-qPCR (D) analysis. (B) Representative western blots from lysates of sorted mTagBFP2^+^ cells, blotted for mTagBFP2, GFP, OGT, O-GlcNAc (RL2), and OGA, with PGK1 as a loading control. (C and D) Statistical analysis of western blot (C) and RT-qPCR (D) results. Ordinary one-way ANOVA was performed for all experiments (*n* = 3 independent replicates). Error bars represent mean ± SEM, with significance indicated as follows: ^∗^ for adjusted *p* < 0.05, ^∗∗^ for *p* < 0.01, ^∗∗∗^ for *p* < 0.001, ^∗∗∗∗^ for *p* < 0.0001, and ns or no mark for non-significant results (adjusted *p* > 0.05).

To validate the FC findings, we transfected OGT-sfGFP mESCs with a subset of plasmids, including OGT wild type, the catalytically inactive OGT-K842M mutant, the gnomAD OGT-I279V variant (Table S2), the pathogenic OGT-CDG N648Y variant (Pravata et al., 2020b), and the mock control (Figure 3A). Transfected mTagBFP2^+^ cells were sorted (Figure 5A) and analyzed by immunoblotting (Figures 5B and 5C) and quantitative reverse-transcription PCR (RT-qPCR) (Figure 5D). Immunoblotting revealed that while the mock control had significantly higher mTagBFP2 expression, all other samples showed comparable expression levels, with no significant difference in exogenous OGT expression (Figures 5B and 5C, Note S1). These results suggest that variations in O-GlcNAc feedback regulation observed in FC were unlikely due to differences in plasmid transfection efficiency. A significant reduction in GFP levels was observed across all samples except K842M (Figures 5B and 5C). Similar trends were observed with endogenous OGT protein and O-GlcNAc levels, although the reduction in endogenous OGT protein and the increase in O-GlcNAc were not always statistically significant (Figures 5B and 5C). Nevertheless, these results align with the reduction in sfGFP fluorescence from FC analysis, highlighting the increased sensitivity of our assay compared to immunoblotting. Interestingly, OGA protein levels showed minimal variability between replicates, with a statistically significant increase in all transfected samples except K842M (Figures 5B and 5C). Notably, the N648Y OGT-CDG variant displayed a less pronounced increase in OGA levels than the wild-type and gnomAD I279V variant (Figure 5C). Compared to the mock control, overexpression of wild-type OGT, the gnomAD I279V variant, and the N648Y OGT-CDG variant resulted in significant reductions in *Ogt* mRNA levels and increases in *Oga* mRNA levels (Figure 5D). Overall, the immunoblotting and RT-qPCR data align with the FC results, collectively demonstrating that changes in OGT-sfGFP fluorescence correlate with the pathogenicity of OGT-CDG variants.

### Changes in OGT-sfGFP fluorescence dissect new OGT-CDG cases from background genetic variants

A key challenge in predicting OGT-CDG variant pathogenicity is co-segregation with variants in other genes linked to ID, such as the A319T OGT-CDG variant (Selvan et al., 2018). We sought to determine whether the variant screening method described here could dissect OGT-CDG variants from such complex genetic backgrounds. Two of the new putative OGT-CDG cases described here (Table S1) possess additional variants in disease-associated genes. The OGT-N848Y variant co-occurs with a nonsense mutation in LMTK3, a brain tyrosine kinase (Kawa et al., 2004; Tomomura et al., 2007) implicated in NMDA receptor trafficking, with knockout models showing cognitive dysfunction and hyperactivity (Inoue et al., 2014; Montrose et al., 2019). For the M1014V OGT variant, a concurrent missense mutation was identified in *LMNA* (LMNA-R545H), a gene encoding A-type lamins associated with laminopathies and, in this case, ID (Worman and Bonne, 2007). Interestingly, cells transfected with the N848Y OGT induced reduced O-GlcNAc dyshomeostasis similar to previously identified pathogenic OGT-CDG variants (Figures 4C and 4D), whereas the M1014V OGT variant did not (Figure 4D), mirroring the effects observed in gnomAD variants (Figure 4B).

To further assess the effects of the N848Y and M1014V variants on OGT activity and stability, these variants were recombinantly produced in *E. coli* in a truncated form (Lazarus et al., 2011). In an assay testing catalytic activity against the well-characterized OGT substrate TAK1-binding protein 1 (TAB1, [Pathak et al., 2012]), the M1014V variant displayed wild-type activity, whereas the N848Y variant was catalytically impaired (35-fold decrease in catalytic efficiency; Figures S5A and S5B). Additionally, the N848Y variant showed reduced thermal stability (ΔT_m_ = 4°C, Figure S5C), while the M1014V variant was unaffected (Figure S5C).

Overall, these findings indicate that the N848Y OGT variant is likely pathogenic, whereas the M1014V variant is likely not. Further investigation is required to clarify the potential contributions of the LMTK3 variant to pathology. Notably, the M1014V variant appears in the gnomAD database, recorded in one hemizygous and one heterozygous individual, and a related variant, M1014T, is reported in two additional heterozygous individuals. Conservation analysis shows that methionine at position 1,014 is not evolutionarily conserved (Figure S3), supporting that variants at this position may not substantially affect protein function. These observations further point to the link between LMNA-R545H and ID. Collectively, these data show that changes in endogenous OGT-sfGFP fluorescence effectively dissect *de novo* OGT-CDG variants from background genetic variants.

## Discussion

We have developed a double-fluorescence strategy to assess exogenous OGT variant activity by quantifying the intensity of O-GlcNAc feedback regulation. This approach allows the first comprehensive investigation of all currently identified pathogenic OGT-CDG variants within an isogenic cellular system. It reveals that most pathogenic variants disrupt O-GlcNAc homeostasis to a lesser degree than wild-type and non-pathogenic gnomAD OGT variants, failing to downregulate endogenous OGT to the same extent. This discovery highlights, for the first time, a unifying feature across most currently known pathogenic variants in a cellular context, suggesting that reduced disruption of O-GlcNAc homeostasis is a common characteristic of OGT-CDG variants.

Using transfection and FC, we introduce the first high-throughput method for predicting the pathogenicity of OGT variants associated with ID. With this approach, we evaluated three newly identified OGT variants in ID patients, identifying N335S and N848Y as likely pathogenic, while M1014V was likely benign. The conserved asparagine at position 335, located on the concave side of the TPR lumen, may play a role in substrate recognition (Joiner et al., 2021), although the exact mechanism by which this substitution affects O-GlcNAc homeostasis remains unclear. The benign nature of M1014V shifts attention to the LMNA-R545H variant, which has been associated with ID, showing incomplete penetrance and variable expressivity in other reports (Chan et al., 2016; Guillín-Amarelle et al., 2018; Magno et al., 2021; Patni et al., 2020). Further research is needed to understand how LMNA-R545H, either alone or in combination with M1014V, contributes to ID. Overall, this method aids in identifying a broader pool of pathogenic OGT-CDG variants for detailed biochemical investigations, ultimately advancing our understanding of OGT-CDG phenotype etiology.

Despite its utility, this screening method has limitations. Some OGT-CDG variants located in the TPR domain show disruptions in O-GlcNAc homeostasis similar to wild-type OGT. Notably, variants like A319T and L254F are found alongside mutations in other genes (e.g., *MED12, PRICKLE3*) (Bouazzi et al., 2015; Vaidyanathan et al., 2017). It is also conceivable that subtle changes in the activity or stability of these TPR domain OGT-CDG variants may not be detectable using the overexpression system described in this study. Additionally, the OGT interactome could play a role in OGT-CDG pathology (Stephen et al., 2021), which this assay does not assess. An alternative approach could include a fluorescence resonance energy transfer-based O-GlcNAc sensor that enables spatiotemporal detection of O-GlcNAc modifications (Carrillo et al., 2006; 2011), although this system relies on the O-GlcNAcylation of a single substrate.

Our study reveals the coordinated regulation of OGT and OGA at both mRNA and protein levels when exogenous OGT variants are overexpressed. Notably, the intensity of this regulation correlates with the activity of the variant being overexpressed, offering a new strategy to explore the mechanisms underlying O-GlcNAc feedback regulation. Current research suggests that this feedback regulation may involve multiple mechanisms at the transcriptional, post-transcriptional, and post-translational levels. For example, under hyper-O-GlcNAcylation conditions, OGT and OGA undergo co-transcriptional splicing to retain a highly conserved intron, targeting OGT mRNA for nonsense-mediated decay while promoting OGA mRNA for protein production (Park et al., 2017; Tan et al., 2020). Additionally, OGA mRNA translation is suppressed by micro-RNA539 following hypoxia-reoxygenation injury in cardiomyocytes (Muthusamy et al., 2015). OGT turnover is also regulated by phosphorylation at Ser20 by checkpoint kinase 1, reducing OGT flux toward the proteasome (Li et al., 2017). Overall, our study highlights that both OGT and OGA, at the transcriptional and protein levels, can serve as sensitive probes for detecting changes in O-GlcNAc homeostasis. This provides a robust system for screening drugs, genes, and signaling pathways involved in O-GlcNAc regulation to dissect the mechanisms underpinning O-GlcNAc homeostasis.

## Experimental procedures

### Ethics and consent

Informed consent for clinical testing and publication was obtained from the parents of all three probands, with approval from the relevant ethics committees (project number provided in supplemental experimental procedures).

### Generation of OGT-sfGFP mES cell line

The mESCs used in this study were derived from E14-TG2a.IV (129/Ola) ES cells as previously described (Pravata et al., 2019). Details of CRISPR-Cas reagent designing, cloning procedures, and daily cell maintenance are provided in supplemental experimental procedures.

### OSMI-4b and TMG treatments

OGT-sfGFP cells cultured in the GMEM-BHK 21 medium were treated with either 10 μM OSMI-4b or 10 μM TMG. All treatments were applied for 24 h, followed by either immunoblotting or FC analysis. Additional details are provided in the supplemental experimental procedures.

### Protein extraction, western blot, and RT-qPCR

Details of the western blot, including protein extraction methods, antibody types, incubation times, and RT-qPCR procedures, are provided in the supplemental experimental procedures.

### Transfection

For all transfection experiments, OGT-sfGFP mESCs were cultured in 2i medium (Ying et al., 2008). Transfection was carried out using Lipofectamine 2000 (Thermo Fisher Scientific), following the method reported before (Tamm et al., 2016) to enhance transfection efficiency. Cells were transfected for 48 h before follow-up analysis, with additional details provided in the supplemental experimental procedures.

### FC analysis

OGT-sfGFP mESCs were analyzed by FC using the gating strategy shown in Figure S2, with additional details in the supplemental experimental procedures.

### Enzyme assays and differential scanning fluorimetry

Wild-type and variant OGT protein (323–1,044 aa) were recombinantly purified from *E. coli* as described previously (Omelková et al., 2023). OGT and variants’ activity against the substrate TAB1 was measured using either western blot or the UDP-Glo assay (Promega). OGT and variants’ stability was measured by detecting protein melting temperatures. Extra details are provided in supplemental experimental procedures.

### Statistics

The statistical analyses in this study were conducted using Prism version 10. The specific statistical methods applied are detailed in the legends accompanying each figure.

## Resource availability

### Lead contact

Requests for further information and resources should be directed to and will be fulfilled by the corresponding author, Daan M. F. van Aalten (daan@mbg.au.dk).

### Materials availability

All materials and reagents including the OGT-sfGFP mESC line generated in this study are available from the corresponding author upon request.

### Data and code availability

Original western blot images and raw FC data reported in this study will be shared by the corresponding author upon request. Any additional information required to reanalyze the data reported in this paper is available from the corresponding author upon request.

## Acknowledgments

This work was funded by a 10.13039/100010269Wellcome Trust Investigator Award (110061), a Novo Nordisk Fonden Laureate award (NNF21OC0065969), and a Villum Fonden Investigator award (00054496) to D.M.F.v.A. H.Y. was funded by the 10.13039/501100004543China Scholarship Council. C.W.M. was funded by the 10.13039/501100000268BBSRC EASTBIO Doctoral Training Programme. This work was supported in part by the Danish Research Institute of Translational Neuroscience - DANDRITE of the Nordic-EMBL Partnership for Molecular Medicine and Lundbeckfonden. The authors thank the FACS Core Facility at Aarhus University and Dr Authier for their support. Parental informed consent was obtained for the participation of these patients, and their contribution is gratefully acknowledged.

## Author contributions

H.Y., A.T.F., and D.M.F.v.A. conceived the study; H.Y. and C.W.M. performed experiments; A.T.F. performed molecular biology; H.Y. and D.M.F.v.A. analyzed data; and H.Y., C.W.M., and D.M.F.v.A. interpreted the data and wrote the manuscript with input from all authors. M.T.B., A.F., P.J.B., and Q.K.G.T. collected and compiled clinical data.

## Declaration of interests

The authors declare no competing interests.

## Declaration of generative AI and AI-assisted technologies in the writing process

During the preparation of this work, the authors used ChatGPT to check the grammar and refine the language to enhance conciseness. After using this tool, the authors reviewed and edited the content as needed and take full responsibility for the content of the publication.

## Supplemental information

Document S1. Figures S1–S5 and Tables S1–S4 and Notes S1 and S2 and Supplemental experimental procedures

Document S2. Article plus supplemental information
