# Supplementary material for: Exploiting O-GlcNAc dyshomeostasis to screen O-GlcNAc transferase intellectual disability variants
Source: Stem Cell Reports. 2024 Dec 19;20(1):102380. doi: 10.1016/j.stemcr.2024.11.010 (PMC11784489; doi:10.1016/j.stemcr.2024.11.010)
Supplement: Document S1. Figures S1–S5 and Tables S1–S4 and Notes S1 and S2 and Supplemental experimental procedures [file mmc1.pdf]

Stem Cell Reports, Volume *20*

Supplemental Information

Exploiting O-GlcNAc dyshomeostasis to screen O-GlcNAc transferase intellectual disability variants

Huijie Yuan, Conor W. Mitchell, Andrew T. Ferenbach, Maria Teresa Bonati, Agnese Feresin, Paul J. Benke, Queenie K.G. Tan, and Daan M.F. van Aalten

Figure S1. Full-length OGT isoform was labelled with sfGFP, related to Figure 1.

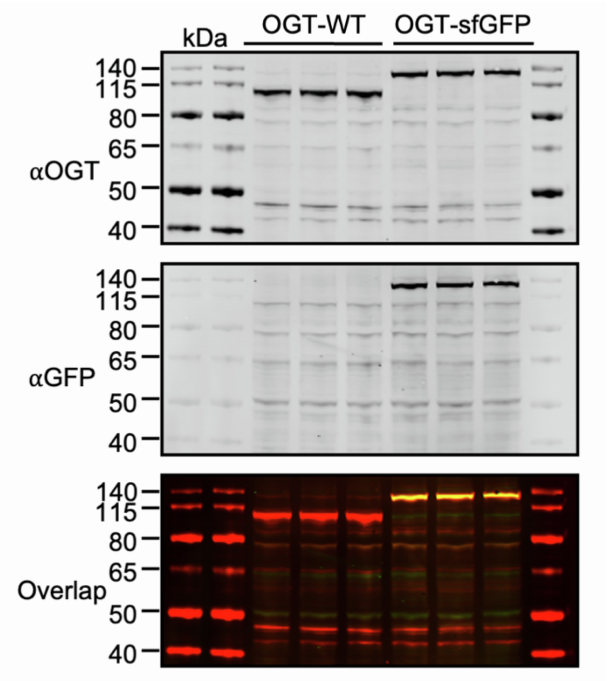

Cell lysates extracted from CRISPR-engineered OGT-sfGFP mESCs and untreated wild type mESCs were subjected to immunoblotting using antibodies against OGT and GFP. OGT was visualised in red, and GFP was visualised in green, with overlapping signals appearing as yellow.

Figure S2. Gating strategy for flow cytometry data analysis, related to Figures 2-5.

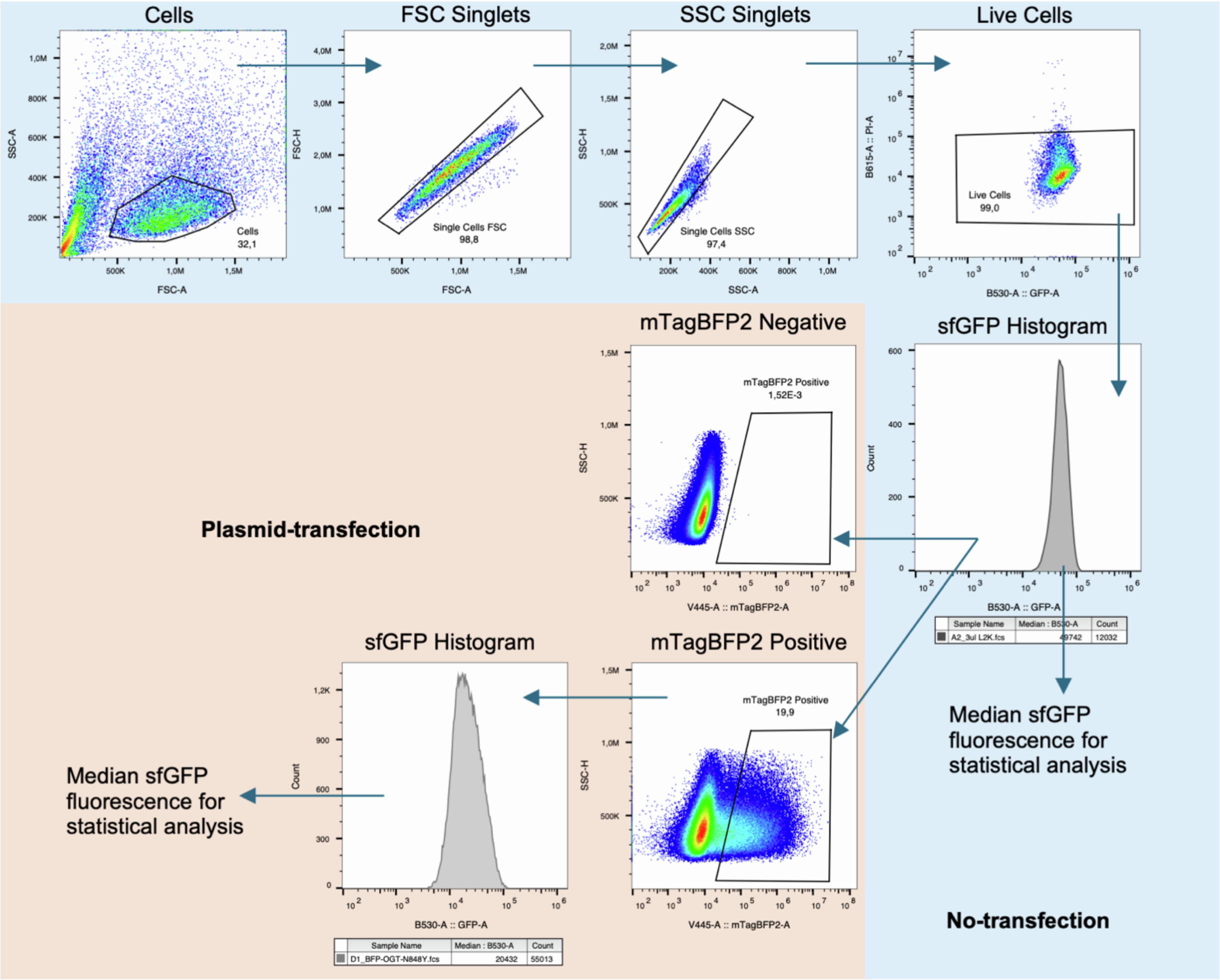

All flow cytometry data were analysed based on this gating strategy. For no-transfection experiments (coloured in light blue), samples were first visualized using forward scatter (FSC) and side scatter (SSC) to gate cells based on size and granularity. These gated cells were then further analysed by comparing FSC height (FSC-H) with FSC area (FSC-A), and SSC height (SSC-H) with SSC area (SSC-A), to isolate cell singlets. The isolated single cells were subsequently plotted on a propidium iodide area (PI-A) versus GFP area (GFP-A) graph to identify live cells. A histogram of sfGFP fluorescence in gated live singlet cells was generated, and the median fluorescence value was extracted for statistical analysis.

For cells transfected with mTagBFP2 labelled plasmids (coloured in light orange), the same procedures were followed with additional steps to select mTagBFP2 positive (mTagBFP2^{+}) cells from the gated live singlet OGT-sfGFP mESCs. A gate was defined based on mTagBFP2 expression, dividing the cells into two groups: mTagBFP2^{+}, indicating successful transfection and expression, and mTagBFP2 negative (mTagBFP2^{-}), indicating no expression of mTagBFP2. The gated mTagBFP2^{+} cells were then visualized in the sfGFP histogram, and the median OGT-sfGFP fluorescence value was extracted for statistical analysis.

Figure S3. Pathogenic and non-pathogenic OGT variants conservation, related to Figures 4-5.

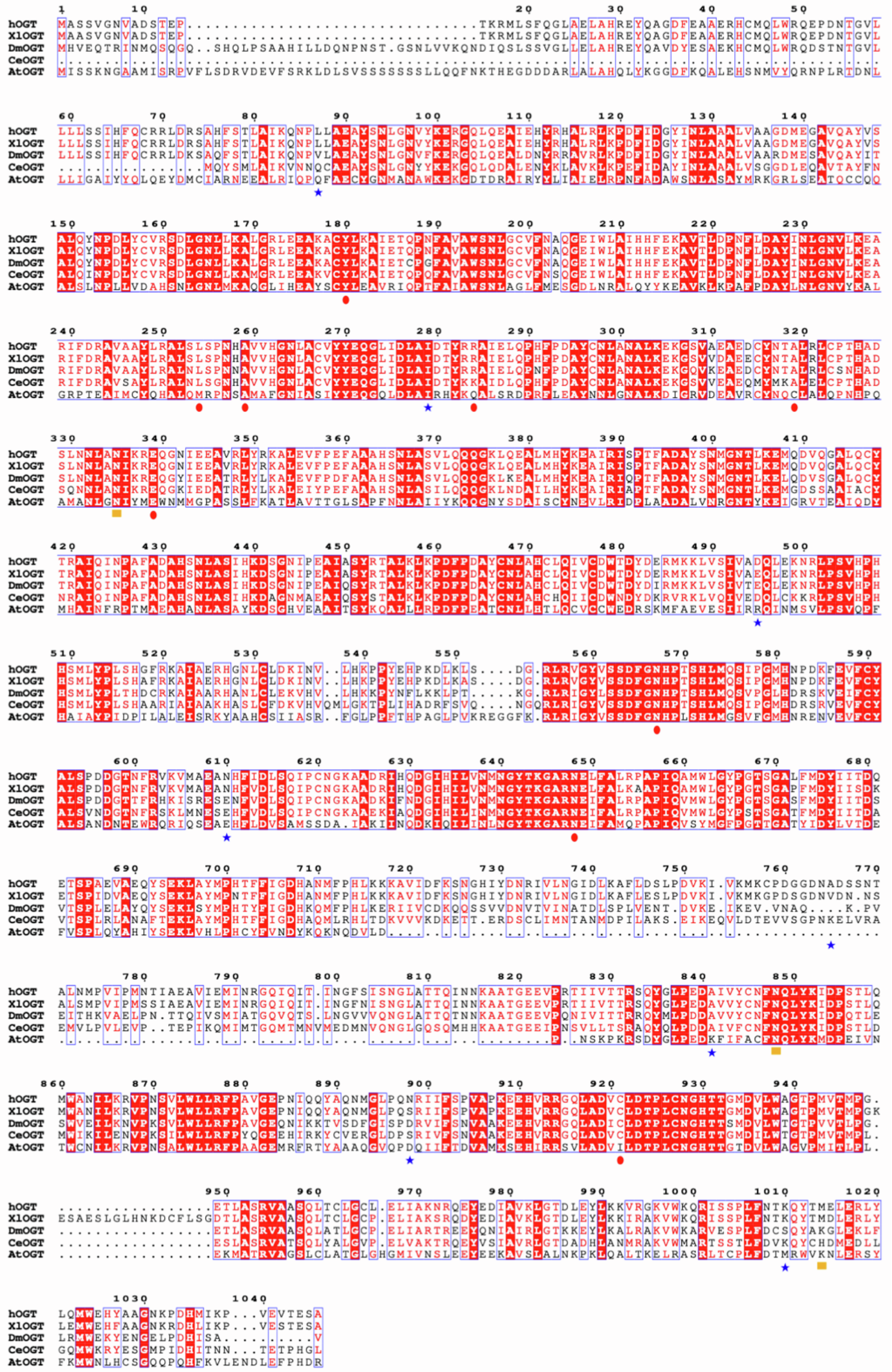

The alignment showcases OGT sequences from various species: human (hOGT, isoform 1), *Xenopus laevis* (*Xl*OGT), *Drosophila melanogaster* (*Dm*OGT), *Caenorhabditis elegans* (*Ce*OGT), and *Arabidopsis thaliana* (*At*OGT). Residues identical across all species are highlighted in red for emphasis, while similar residues are coloured red and enclosed in blue boxes for clarity. Blue star symbols pinpoint the locations of GNOMAD non-pathogenic OGT variants, whereas red solid circles highlight the positions of currently identified pathogenic OGT-CDG variants. Furthermore, orange solid squares denote the locations of newly reported ID-associated OGT variants. Sequence annotation is completed using the online ENDscript server[1].

Figure S4. Representative density plots for mTagBFP2^{+} cell selection, related to Figure 4.

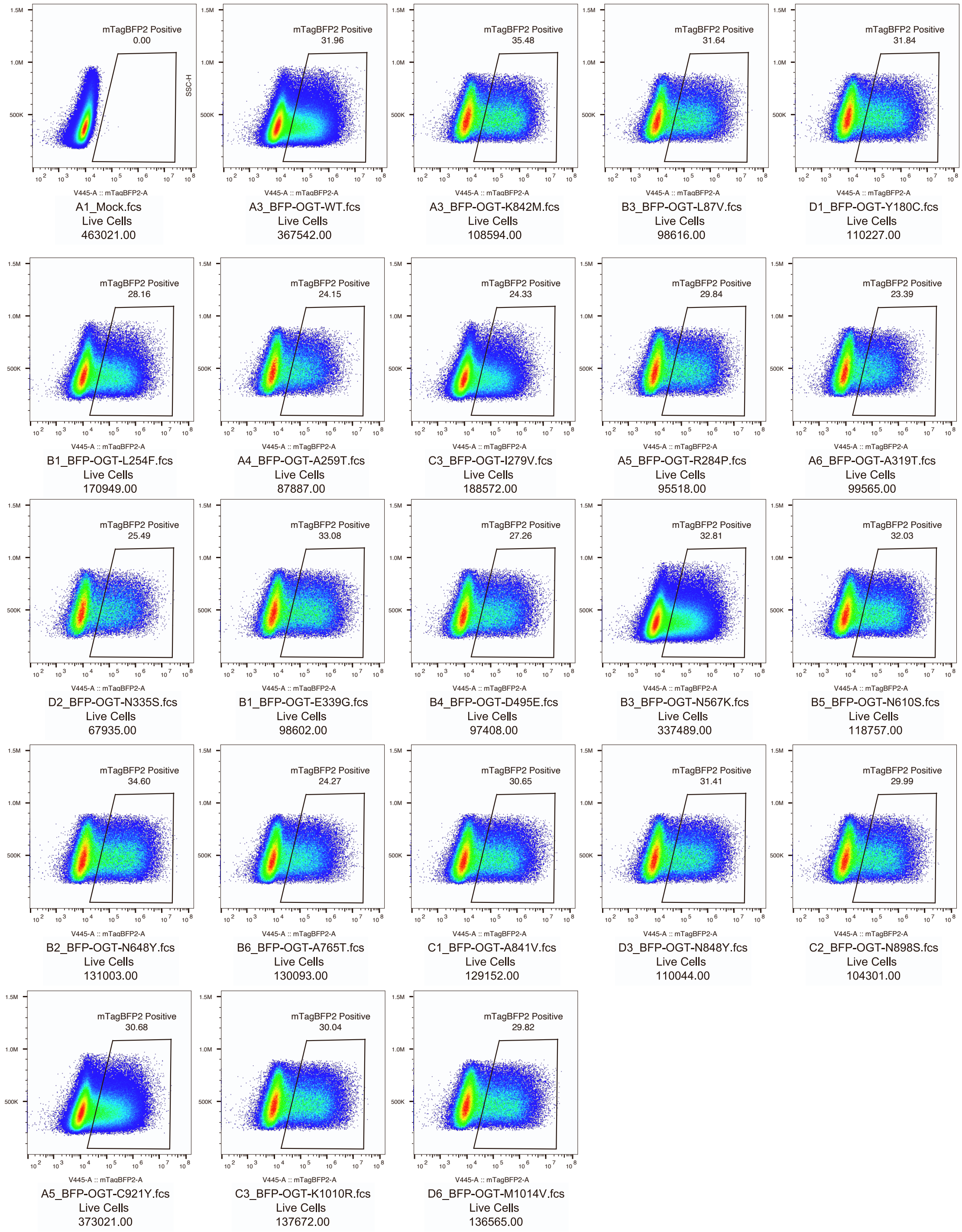

The representative density plots for each type of OGT variant transfected sample are shown, with the specific OGT variant labelled at the bottom of each plot. These plots illustrate the transfection efficiency for each OGT variant and demonstrate the selection of mTagBFP2^{+} cells for further analysis.

Figure S5. The N848Y OGT variant but not the M1014V variant exhibits reduced catalytic efficiency and stability, related to Figure 4.

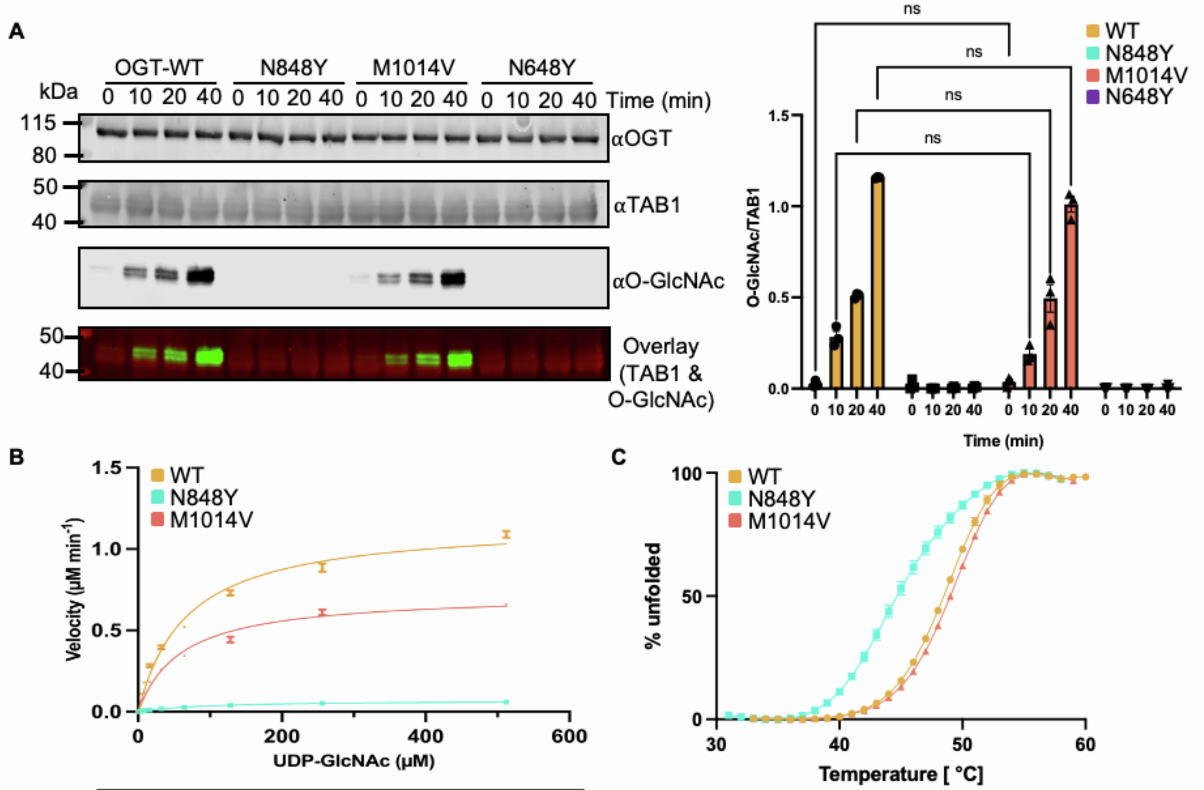

Variant	V_{max} ($nM \cdot s^{-1}$)	K_m (μM)	k_{cat}/K_m ($M^{-1} \cdot s^{-1}$)
WT	1.1 ± 0.033	66 ± 6	0.35
N848Y	0.07 ± 0.002	95 ± 6	0.01
M1014V	0.72 ± 0.020	60 ± 5	0.24

Variant	T_m (°C)
WT	48.37 ± 0.02
N848Y	44.73 ± 0.11
M1014V	48.96 ± 0.03

(A) The N848Y OGT variant but not the M1014V variant showed reduced catalytic activity towards TAB1 *in vitro*. The recombinantly purified wild type OGT or its variants were incubated with TAB1 and UDP-GlcNAc for the indicated time points prior to O-GlcNAc immunoblotting analysis (RL2). Statistical analysis of the immunoblotting data is shown on the right, where an Ordinary One-way ANOVA was performed. The results are presented as the mean O-GlcNAc signal normalized to the TAB1 loading control, with standard error of the mean indicated. The analysis is based on n = 3 independent experiments, with "ns" denoting an adjusted *P* value greater than 0.05.

(B) The N848Y OGT variant but not the M1014V variant presented reduced enzyme kinetics towards TAB1 *in vitro.* The recombinantly purified OGT variants were incubated with excess TAB1 for 40 min during different UDP-GlcNAc concentrations. The reaction was stopped and detected by the UDP-Glo™ kit. Error bars in the graph depict the standard error of the mean, based on three repeated experiments performed on the same day. The kinetics are shown in the table below.

(C) The N848Y OGT variant but not the M1014V variant displayed reduced thermal stability. The recombinantly purified wild type OGT or its variants were analysed in differential scanning fluorimetry. The melting curves and associated T_m values are presented with their standard error of the mean, based on five repeated experiments performed on the same day.

Table S1. Clinical phenotypes of all three probands

OGT variant (reference sequence GenBank: NM_181672.3)	c.1004A>G, p.(N335S)	c.2542A>T, p.(N848Y)	c.3040 A>G, p.(M1014V)
Protein domain	N terminal TPR	Catalytic Region	Catalytic Region
Inheritance	Maternal	*De novo*	
Other genetic variant	-	LMTK3-W464*	LMNA-R545H
Gender	M	F	M
Low birth weight	+	-	+
Hypotonia	+	+	+
Drooling	-	+	-
Developmental delay	+	+	+
Speech and language delay	+	+	+
IQ	WPPSI-III: IQ 79; verbal scale IQ 83; performance scale IQ 81; general speech scale IQ 89	MSEL age 33 moths: Verbal DQ 79, FM 20 months and GM 18 months	Borderline
Epilepsy	-	-	-
Dystonia	-	-	-
Behavioural problems	+	+	-
Brain abnormalities	-	-	-
Eye abnormalities	+	+	-
Ear abnormalities/hearing impairment	-	-	-
Microcephaly	-	+	-
Short stature	-	-	-
Cranio-facial dysmorphisms	+	-	+
Dolicocephaly	-	-	-
Frontal hair upsweep	+	-	
Broad nasal root	-	-	
Full/long philtrum	-	-	
Full lips	+	-	
Non cranio-facial dysmorphisms	+	-	
Clinodactyly	+	-	
Long thin fingers	-	-	
Genital/reproductive abnormalities	-	-	

[a] In the table, a “+” denotes that the patient exhibits the phenotype, while a “−” indicates the absence of the phenotype.

Table S2. Pathogenicity and allele frequency of OGT variants, related to Figures 3-5.

Plasmid Name	Variant Type	Allele Count	Additional information
mTagBFP2-OGT-Y180C	Pathogenic	0	
mTagBFP2-OGT-L254F	Pathogenic	0	
mTagBFP2-OGT-A259T	Pathogenic	0	
mTagBFP2-OGT-R284P	Pathogenic	0	
mTagBFP2-OGT-A319T	Pathogenic	0	
mTagBFP2-OGT-E339G	Pathogenic	0	
mTagBFP2-OGT-N567K	Pathogenic	0	
mTagBFP2-OGT-N648Y	Pathogenic	0	
mTagBFP2-OGT-C921Y	Pathogenic	0	1 additional allele count of C921S
mTagBFP2-OGT-I279V	GNOMAD	115	
mTagBFP2-OGT-E495D	GNOMAD	106	
mTagBFP2-OGT-N610S	GNOMAD	44	
mTagBFP2-OGT-L87V	GNOMAD	29	
mTagBFP2-OGT-K1010R	GNOMAD	29	
mTagBFP2-OGT-A765T	GNOMAD	22	
mTagBFP2-OGT-A841V	GNOMAD	22	
mTagBFP2-OGT-N898S	GNOMAD	21	
mTagBFP2-OGT-N335S	Unknown	0	
mTagBFP2-OGT-N848Y	Unknown	0	
mTagBFP2-OGT-M1014V	Unknown	2	2 additional allele counts of M1014T

The table outlines all types of mTagBFP2-labeled OGT plasmids utilized for transfecting OGT-sfGFP cells. The first column lists the names of the plasmids, each specifying the type of missense mutation present. The second column categorizes the type of each variant: those identified in the literature as causal for ID are labelled ‘Pathogenic’; variants sourced from the general population database, gnomAD[2], are labelled as ‘GNOMAD’; and new ID-associated OGT variants reported in this study are denoted as ‘Unknown’. The third column indicates the allele count of occurrence of the OGT variant from the gnomAD database. The fourth column provides additional information, indicating whether other missense variants at the same residue are reported in the gnomAD database.

Table S3. List of primers used to generate all types of mTagBFP2 labelled OGT plasmid for overexpression in OGT-sfGFP mESCs, related to Figures 3-5.

Plasmid Name	Primers
mTagBFP2-P2A-OGT(K842M)	ctttaatcaactgtacatgatcgacccgtctaccctg, cagggtagacgggtcgatcatgtacagttgattaaag
mTagBFP2-P2A-OGT(Y180C)	gaagcgaaagcctgttgcctgaaagcgattgaaac, gtttcaatcgctttcaggcaacaggctttcgcttc
mTagBFP2-P2A-OGT(L254F)	cctgcgtgcactgagtttttccccgaaccatgccg, cggcatggttcggggaaaaactcagtgcacgcagg
mTagBFP2-P2A-OGT(A259T)	gtccccgaaccataccgtggttcacgg, ccgtgaaccacggtatggttcggggac
mTagBFP2-P2A-OGT(R284P)	gctattgatacctatcgtccggcgattgaactgcagccg, cggctgcagttcaatcgccggacgataggtatcaatagc
mTagBFP2-P2A-OGT(E339G)	ctggcgaacattaagcgtggccaaggcaacattgaagaagc, gcttcttcaatgttgccttggccacgcttaatgttcgccag
mTagBFP2-P2A-OGT(A319T)	gaagattgttataacaccacactgcgtctgtgcccg, cgggcacagacgcagtgtggtgttataacaatcttc
mTagBFP2-P2A-OGT(N567K)	gctctgattttggtaaacatccgaccagccacc, ggtggctggtcggatgtttaccaaaatcagagc
mTagBFP2-P2A-OGT(N648Y)	caccaaaggcgcccgttatgaactgttcgcc, ggcgaacagttcataacgggcgcctttggtg
mTagBFP2-P2A-OGT(C921Y)	cagctggcagacgtgtatctggataccccgctg, cagcggggtatccagatacacgtctgccagctg
mTagBFP2-P2A-OGT(Leu87Val)	gctattaaacaaaatccggtgctggcagaagcttatagc, gctataagcttctgccagcaccggattttgtttaatagc
mTagBFP2-P2A-OGT(Ile279Val)	cctgatcgacctggctgtggatacctatcgtcgcg, cgcgacgataggtatccacagccaggtcgatcagg
mTagBFP2-P2A-OGT(Asp495Glu)	gtctctattgtggccgaacagctggaaaagaacc, ggttcttttccagctgttcggccacaatagagac
mTagBFP2-P2A-OGT(Asn610Ser)	gtgaaagttatggcagaagcttctcacttcatcgatctgtcac, gtgacagatcgatgaagtgagaagcttctgccataactttcac
mTagBFP2-P2A-OGT(Ala765Thr)	gatggcggtgacaacaccgatagttccaacac, gtgttggaactatcggtgttgtcaccgccatc
mTagBFP2-P2A-OGT(A841V)	ggcctgccggaagatgtcattgtgtattgcaac, gttgcaatacacaatgacatcttccggcaggcc
mTagBFP2-P2A-OGT(N898S)	catgggcctgccgcagagtcgtattatcttcag, ctgaagataatacgactctgcggcaggcccatg
mTagBFP2-P2A-OGT(Lys1010Arg)	cgccgctgtttaataccagacagtatacgatggaac, gttccatcgtatactgtctggtattaaacagcggcg
mTagBFP2-P2A-OGT(N335S)	ctgaacaacctggcgagcattaagcgtgaacaag, cttgttcacgcttaatgctcgccaggttgttcag
mTagBFP2-P2A-OGT(N848Y)	gtgtattgcaacttttatcaactgtacaag, cttgtacagttgataaaagttgcaatacac
mTagBFP2-P2A-OGT(M1014V)	gtttaataccaaacagtatacggtggaactggaacgcctg, caggcgttccagttccaccgtatactgtttggtattaaac

Table S4. Sequences of qPCR primers for *Oga*, *Ogt* mRNA measurement, related to Figure 5.

Primer	Sequence (5’ -> 3’)
Actb Forward	AGATCAAGATCATTGCTCCTCCT
Actb Reverse	ACGCAGCTCAGTAACAGTCC
18S Forward	CTCAACACGGGAAACCTCAC
18S Reverse	CGCTCCACCAACTAAGAACG
Ogt Forward	CCCCCTGAGCCCTTCAAAAC
Ogt Reverse	TCGTTGGTTCTGTACTGTCGG
Oga Forward	TGCAGTGGTTAGGGTGTCG
Oga Reverse	AGCAAACGCTGGAACTCTCC

Note S1. Exogenous and Endogenous OGT, related to Figure 5.
Immunoblotting of sorted mTagBFP2$^+$ mESCs with an OGT antibody distinguishes transfected exogenous OGT from endogenous OGT-sfGFP by molecular size, as the sfGFP fusion makes endogenous OGT-sfGFP larger than exogenous transfected OGT.

Note S2. Transfection scale and duration, related to Figures 3-5.
The cell density and the amount of transfection reagents can be scaled up based on the requirements of the subsequent analysis. A DNA to L2K transfection reagent ratio of 1:2 or 1:3 is recommended, as shown in previous experiments[3], to maintain high transfection efficiency while minimizing cytotoxicity. Various transfection durations were tested, with peak exogenous plasmid expression observed at 48 hours post-transfection, which was then chosen for analysing endogenous OGT-sfGFP fluorescence. However, differences in O-GlcNAc feedback regulation between cells transfected with wild type OGT and the OGT-K842M mutant remained evident 72 hours post-transfection.

Supplemental experimental procedures

Ethics and consent

The proband with NM_181672.3: c.1004A>G, p.(N335S) entered the study was approved by the Ethics Committee of IRCCS Burlo Garofolo (Trieste, Italy) with the project number RC10/22, subcentre: L310_22. The proband with NM_181672.2: c.2542A>T, p.(N848Y) was enrolled in the Clinical and Basic Investigations in Congenital Disorders of Glycosylation study at Mayo Clinic in Rochester Minnesota, USA, IRB number 19-005187. The proband with NM_181672.3: c.3040 A>G, p.(M1014V) enrolled in this study was approved by Memorial Healthcare System, Protocol MH # 208.01.

CRISPR/Cas9 reagents and other cloning procedures

The C-terminal sfGFP CRISPR/Cas9 reagents were based on the repair template used for adding a 3xHA tag to mouse OGT[4]. Restrictionless cloning was used to replace the 3xHA tag with sfGFP. The change was confirmed by DNA sequencing. The guide RNA (gRNA) expressing plasmids were used directly as before[4]. Codon-optimised OGT obtained from Genscript was used for exogenous OGT overexpression. The codon bias was originally intended for *E. coli* expression but the ORF has been shown to function in mammalian cells. The sequence was first cloned into pCMV-(c)-HA as a *Bam*HI-*Not*I fragment. This included an upstream CCACC sequence partly coded by the *Bam*HI site. This construct was then cut using *Not*I, dephosphorylated and the BFP sequence was obtained from pBAD-mTagBFP and a P2A sequence was added by the forward primer. The P2A sequence was added by sequential nested PCRs. The final PCR product was cut with *Not*I and *Bsp120i*. This was cloned into the precut vector and this step resulted in the destruction of the *Not*I site at the 3' end of the ORF, but the retention of the *Not*I site between the P2A site and the end of the hOGT ORF. All clones were confirmed with DNA sequencing. Mutations of the hOGT cloned in BP2 were carried out by site-directed mutagenesis based on the Stratagene Quikchange mutagenesis kit, but Kod polymerase was used and *Dpn*I from Thermo Fisher. Sequences of all mutants were confirmed by DNA sequencing. The primer sequences used to generate all human OGT variants for transfection are provided in Table S3.

Codon-optimized *OGT* sequence is provided below, where OGT was shown in lowercase letters, the *Not*I site is shown in uppercase and coloured red, the P2A sequence is shown in uppercase and marked in yellow, and the mTagBFP2 sequence is shown in upper case and coloured blue.

atggcttcaagtgtgggtaatgtggctgattcgacggaaccgaccaaacgtatgctgtcttttcaaggtctggcggaactggctcatcgtgaatat
caggcgggtgattttgaagcggccgaacgtcattgtatgcaactgtggcgccaggaaccggataacaccggcgtgctgctgctgctgagctct
atccattttcagtgccgtcgcctggatcgtagcgcacacttctctacgctggctattaaacaaaatccgctgctggcagaagcttatagcaacctg
ggtaatgtttacaaagaacgcggccaactgcaggaagcgatcgaacattatcgtcacgccctgcgcctgaaaccggattttatcgacggctac
attaacctggcagctgcgctggtcgccgcaggtgatatggaaggcgccgttcaagcatatgtctctgcgctgcagtataacccggacctgtact
gtgtgcgtagtgatctgggtaatctgctgaaagcactgggccgcctggaagaagcgaaagcctgttacctgaaagcgattgaaacccagccg
aactttgcagtcgcttggtctaacctgggttgcgtgttcaatgcacagggcgaaatctggctggctattcatcactttgaaaaagcggtgaccctg
gacccgaatttcctggatgcatatattaacctgggtaatgttctgaaagaagcgcgtatcttcgatcgtgcagtcgctgcatacctgcgtgcactg
agtctgtccccgaaccatgccgtggttcacggtaatctggcatgtgtgtattacgaacagggcctgatcgacctggctattgatacctatcgtcgc
gcgattgaactgcagccgcattttccggatgcctactgcaacctggcgaatgccctgaaagaaaaaggttcggttgcagaagctgaagattgtt
ataacaccgcactgcgtctgtgtccgacccatgctgattccctgaacaacctggcgaacattaagcgtgaacaaggcaacattgaagaagcc
gtccgtctgtatcgtaaagcgctggaagtctttccggaattcgcggcggcacatagtaacctggcctccgtgctgcagcaacagggcaagctg
caggaagctctgatgcactataaagaagcgattcgtatctctccgacctttgccgatgcatacagtaacatgggtaatacgctgaaagaaatgc
aagacgtgcagggcgccctgcaatgttatacccgcgcaattcagatcaacccggctttcgcggatgcccattcaaatctggcatcgattcacaa
agactccggcaacatcccggaagcaattgcttcatatcgtaccgcgctgaagctgaaaccggattttccggacgcttactgcaatctggcgcat
tgtctgcagatcgtctgcgattggacggattatgacgaacgtatgaaaaagctggtctctattgtggccgatcagctggaaaagaaccgcctgc
cgtcagttcatccgcatcactcgatgctgtacccgctgagccatggtttccgtaaagcgatcgccgaacgccacggcaacctgtgcctggataa
gattaatgttctgcataaaccgccatatgaacacccgaaggacctgaaactgagtgatggtcgtctgcgcgttggctacgtcagctctgattttgg
taaccatccgaccagccacctgatgcagtctattccgggcatgcataatccggataagtttgaagtgttctgttatgcgctgtccccggatgacgg
tacgaactttcgtgtgaaagttatggcagaagctaatcacttcatcgatctgtcacagattccgtgcaacggcaaggctgcggaccgcatccatc
aagatggcattcacatcctggttaacatgaatggttacaccaaaggcgcccgtaatgaactgttcgccctgcgtccggcaccgattcaggcaat
gtggctgggttatccgggtaccagcggtgccctgttcatggactacattatcaccgatcaagaaacgtcgccggcagaagtggctgaacagta
tagcgaaaaactggcctacatgccgcataccttttcatcggtgatcatgctaacatgtttccgcacctgaaaaagaaagcggttattgacttcaa
atcgaatggtcacatctatgataaccgtattgtcctgaatggcatcgacctgaaggcgtttctggacagcctgccggatgtgaaaattgttaagat
gaaatgtccggatggcggtgacaacgccgatagttccaacaccgcactgaatatgccggtgattccgatgaacacgatcgcggaagccgtta
tcgaaatgattaatcgcggccaaattcagatcaccattaacggtttcagcatttctaatggcctggcgaccacgcagatcaacaataaagccgc
aaccggtgaagaagtgccgcgtacgattatcgttaccacgcgctctcagtatggcctgccggaagatgccattgtgtattgcaactttaatcaact

gtacaagatcgacccgtctaccctgcagatgtgggcaaacattctgaaacgtgtcccgaatagtgtgctgtggctgctgcgctttccggccgttg
gtgaaccgaacatccaacagtatgcacaaaacatgggcctgccgcagaatcgtattatcttcagcccggtcgccccgaaagaagaacatgt
gcgtcgcggtcagctggcagacgtgtgtctggataccccgctgtgcaatggtcacaccacgggcatggatgttctgtgggctggtaccccgatg
gtcacgatgccgggcgaaaccctggcaagtcgtgttgctgcctcccagctgacctgcctgggttgtctggaactgattgctaagaaccgccag
gaatatgaagacatcgcggttaaactgggtaccgatctggaatacctgaagaaagttcgtggcaaggtctggaaacaacgcatttcatcgccg
ctgtttaataccaaacagtatacgatggaactggaacgcctgtacctgcaaatgtgggaacactacgcagcaggcaataagccggaccatat
gattaagccggtggaagtgaccgaaagcGCGGCCGCGGGAAGCGGAGCTACTAACTTCAGCCTGCTGAAGC
AGGCTGGAGACGTGGAGGAGAACCCTGGACCTATGAGCGAGCTGATTAAGGAGAACATGCACA
TGAAGCTGTACATGGAGGGCACCGTGGACAACCATCACTTCAAGTGCACATCCGAGGGCGAAG
GCAAGCCCTACGAGGGCACCCAGACCATGAGAATCAAGGTGGTCGAGGGCGGCCCTCTCCCCT
TCGCCTTCGACATCCTGGCTACTAGCTTCCTCTACGGCAGCAAGACCTTCATCAACCACACCCAG
GGCATCCCCGACTTCTTCAAGCAGTCCTTCCCTGAGGGCTTCACATGGGAGAGAGTCACCACAT
ACGAAGACGGGGGCGTGCTGACCGCTACCCAGGACACCAGCCTCCAGGACGGCTGCCTCATCT
ACAACGTCAAGATCAGAGGGGTGAACTTCACATCCAACGGCCCTGTGATGCAGAAGAAAACACT
CGGCTGGGAGGCCTTCACCGAGACGCTGTACCCCGCTGACGGCGGCCTGGAAGGCAGAAACG
ACATGGCCCTGAAGCTCGTGGGCGGGAGCCATCTGATCGCAAACGCCAAGACCACATATAGATC
CAAGAAACCCGCTAAGAACCTCAAGATGCCTGGCGTCTACTATGTGGACTACAGACTGGAAAGA
ATCAAGGAGGCCAACAACGAGACCTACGTCGAGCAGCACGAGGTGGCAGTGGCCAGATACTGC
GACCTCCCTAGCAAACTGGGGCACAAGCTTAATTAA

Cell culture and maintenance

The mESCs used in this study were cultured without feeder cells on 0.1% gelatin-coated plates using GMEM-BHK 21 medium (Gibco, ref 11710035) supplemented with 10% [v/v] fetal bovine serum (Gibco, A5256701), 1 mM sodium pyruvate (Gibco, 11360088), 1000 units/mL LIF (MERK, ESG1107), 0.1 mM MEM nonessential amino acids (Gibco, 11140050), and 0.1 mM 2-mercaptoethanol (Gibco, 31350010) in a 5% CO_2 environment at 37 ºC. Cells were maintained in an undifferentiated state without changes in morphology and replication speed and were passaged at a 1:5 ratio. For freezing, cells were preserved using the described GMEM-BHK21 medium supplemented with 10% DMSO. No bacterial contamination was detected throughout the experiment. Cells tested negative for mycoplasma before the experiment began.

To improve transfection efficiency, OGT-sfGFP mESCs cultured in GMEM-BHK 21 medium was switched to 2i medium[5], consisting of 50% DMEM/F-12 with GlutaMAX™ Supplement (Gibco, 31331-093), 50% Neurobasal (Gibco, 21103049), 1X N-2 Supplement (100X) (Gibco, 17502048), and 1X B-27 Supplement (50X, 17504044), supplemented with 1 μM MEK inhibitor PD0325901 (MERK, PZ0162), 3 μM GSK3 inhibitor CHIR99021 (MERK, SML1046), 1000 units/ml LIF (MERK, ESG1107), and 2% [v/v] FBS (Gibco, A5256701). OGT-sfGFP mESCs were cultured in 2i medium for all transfection experiments.

Generation of OGT-sfGFP mES cell line

To tag endogenous OGT with sfGFP, mESCs cultured in GMEM-BHK 21 medium were transfected with Cas9 D10A nickase[6], designed gRNA sequences, and a repair template using Lipofectamine 3000, following the manufacturer's protocol. Details of CRISPR/Cas reagents and cloning procedures are provided above. Post-transfection, the cells underwent puromycin selection at 1 μg/ml for 48 h. The successful tagging was confirmed through restriction enzyme analysis and genomic DNA sequencing. For detailed procedures of the CRISPR/Cas editing, refer to our previously published paper[4].

OSMI-4b and Thiamet G (TMG) treatment

For immunoblotting, 0.5 million OGT-sfGFP mESCs were seeded into 6-well plates and, after 24 hours, treated with 10 μM OSMI-4b or TMG, both at a final concentration of 0.1% DMSO, along with a 0.1% DMSO control and a no-treatment control. After 24 hours of treatment, the cells were harvested for immunoblotting. This experiment was independently repeated three times on different days, each using cells from different passage numbers.

For flow cytometry analysis, 0.1 million OGT-sfGFP mESCs were seeded into 12-well plates. After 24 hours of growth, the cells were treated with 10 μM OSMI-4b or TMG, along with a 0.1% DMSO control. Following 24 hours of treatment, the cells were analysed by flow cytometry (see gating strategy in the No-transfection section of Figure S2). The median sfGFP fluorescence from the sfGFP histogram was used for statistical analysis. This experiment was also independently repeated three

times on different days, with cells from different passage numbers. Stock solutions of OSMI-4b and TMG were prepared in 100% DMSO at a concentration of 10 mM.

Western blot

The harvested cells were washed with PBS and lysed in RIPA buffer (Thermo Fisher). The quantity of extracted crude protein was measured using the PierceTM 660 nm Protein Assay Reagent (Thermo Fisher). 20 µg of crude cell lysate was loaded onto 4-12% Bis-Tris NuPAGE gels (Invitrogen) and transferred to nitrocellulose membranes using a semi-dry transfer system. The transferred membrane was first blocked with 5% BSA [w/v] in TBS-T buffer for at least 1 hour at room temperature. Following this, the membrane was incubated with primary antibodies for the specified times listed below. After primary antibody incubation, the membrane was washed three times with TBS-T buffer, each wash lasting five minutes. The membrane was then incubated with IRDye 680RD (Licor) or IRDye 800CW secondary antibodies for 1 hour at room temperature. Imaging was performed using the Odyssey CLx (Licor) system with 700 nm and 800 nm channels.

The primary antibodies used in this study include OGT (Sigma, DM-17, 1:1000, 2 hours at room temperature), RL2 for O-GlcNAc detection (Thermo Fisher, 1:500, overnight at 4°C), GFP (Proteintech, 66002-1-Ig, 1:5000, 1.5 hours at room temperature), PGK1 (Proteintech, 17811-1-AP, 1:5000, 1.5 hours at room temperature), OGA (Thermo Fisher, SAB4200267, 1:1000, 4 hours at room temperature), and pan-RFP for mTagBFP2 detection (Chromotek, pabr1, 1:5000, 1.5 hours at room temperature).

RT-qPCR analysis

Total RNA was extracted from the sorted mTagBFP2$^+$ cells using the RNAeasy Kit (Qiagen), followed by reverse transcription of 500 ng of RNA using the qScript cDNA Synthesis Kit (Quantabio) according to the manufacturer's protocol. qPCR was then performed using Perfecta SYBR Green FastMix for iQ (Quantabio), as previously described[4], with *Actb* and 18S serving as internal controls. Each sample was analysed in triplicate, with the mean mRNA abundance considered as one independent replicate. The experiment was repeated three times, with each selected plasmid transfected into OGT-sfGFP cells on different days, using cells of different passage numbers. This provided three independent replicates per plasmid type for statistical analysis. Primers are listed in Table S4.

Transfection

For all transfection experiments, OGT-sfGFP mESCs were cultured in 2i medium[5] to improve transfection efficiency (details of medium composition provided in Cell culture and maintenance section above). The transfection method reported before[3] was used (see also Note S2), where 1.5 µg of DNA plasmids was mixed with 3 µl of Lipofectamine 2000 (L2K) in 100 µl of Opti-MEM according to the manufacture's protocol. A suspension of 2.5 × 10^5 cells was incubated with the transfection complex for 5 min before being seeded into 12-well plates. After a 24 h incubation, the cell medium was refreshed, and following an additional 24 h period, cells were harvested for flow cytometry analysis. This transfection procedure applies to all experiments without cell sorting.

OGT-sfGFP mESCs were also transfected with a subset of plasmids, followed by sorting of mTagBFP2$^+$ cells and subsequent RT-qPCR analysis and immunoblotting. For RT-qPCR analysis, 3 µg DNA plasmid was mixed with 6 µl L2K transfection reagent in 250 µl Opti-MEM medium according to the manufacture's protocol. Then a suspension of 0.6 x 10^6 OGT-sfGFP cells were incubated with the transfection complex for 5 minutes before seeded onto 6-well plate. After a 24 h incubation, the cell medium was refreshed, and following an additional 24 h period, cells were harvested for sorting. The gating strategy followed the same principles presented in Figure S1, with approximately one million mTagBFP2$^+$ cells per plasmid type sorted for RT-qPCR analysis after transfection.

For immunoblotting, 6 µg DNA plasmid was mixed with 18 µl L2K transfection reagent in 600 µl Opti-MEM medium according to the manufacture's protocol. Then a suspension of 2.0 x 10^6 OGT-sfGFP cells were incubated with the transfection complex for 5 minutes before seeded onto 100 mm petri dish. After a 24 h incubation, the cell medium was refreshed, and following an additional 24 h period, cells were harvested for sorting. The gating strategy followed the same principles presented in Figure S1, with at least two million mTagBFP2$^+$ cells per plasmid type sorted for immunoblotting.

Representative density plots for all types of plasmid transfection are shown in Figure S4.

Flow cytometer configuration
For sample analysis, NovoCyte 3000 flow cytometer equipped with three lasers (405nm, 488nm, and 640nm) and 13 fluorescence detectors (Agilent, Santa Clara, CA) was utilized. mTagBFP2 fluorescence was detected using a 405 nm laser combined with a 445/45 detector. The 488 nm laser, along with a 488/10 detector, was employed for forward scatter (FSC) and side scatter (SSC) measurements. The detection of sfGFP fluorescence was carried out using a 530/30 detector, and PI detection was achieved with a 615/20 detector. Data acquisition was conducted using the NovoExpress package (version 1.6.2, Agilent, Santa Clara, CA) and data analysis was performed using FlowJo™ (version 10). The gating strategy employed is detailed in Figure S2.

For sorting cells, FACSAria III with 4 lasers (405 nm, 488 nm, 561 nm, 633 nm) and 12 fluorescence detectors (BD Biosciences, San Jose, CA) was utilized. mTagBFP2 fluorescence was detected using a 405 nm laser combined with a 450/40 detector. The 488 nm laser, along with a 488/10 detector, was employed for forward scatter (FSC) and side scatter (SSC) measurements, and detection of sfGFP fluorescence was carried out using a 530/30 detector. PI was detected using a 561 nm laser, along with a 610/20 detector. Data acquisition was conducted using the BD FACSDiva Software version 8.0.2 (BD Biosciences, San Jose, CA).

Flow cytometry analysis
Harvested cells were centrifuged at 300 rpm for 3 min, after which the supernatant was discarded. The cell pellet was then resuspended in a non-sticky cell sorting buffer, which comprised PBS supplemented with 1% BSA, 2.5 mM EDTA, and 25 mM HEPES buffer. Propidium iodide (PI) was added to a final concentration of 10 µg/ml. Samples were stored on ice until analysis, with the flow cytometer configuration listed above. OGT-sfGFP mESCs treated with OSMI-4b or TMG were analysed using the No-transfection gating strategy in Figure S2, while all transfected cells were analysed with the Plasmid-transfection gating strategy in Figure S2. Each sample's OGT-sfGFP fluorescence intensity is determined by the median sfGFP fluorescence value from the sfGFP histogram, which is then used for statistical analysis.

Enzyme activity analysis and Differential scanning fluorimetry
In the *in vitro* O-GlcNAcylation assays, purified OGT (323-1044 aa) was incubated at a concentration of 100 nM with 1 µM of the TAB1 substrate (7-420 aa) in the presence of 1 mM UDP-GlcNAc for multiple 10 min intervals before O-GlcNAc Western blot analysis (using RL2 antibody). The pathogenic N648Y OGT-CDG variant[7] served as a negative control. This procedure was independently replicated three times on separate days. For kinetic studies, 50 nM of purified OGT or its variants (323-1044 aa) were incubated with 20 µM TAB1 (7-420 aa) and varying UDP-GlcNAc concentrations, from 512 µM to 0 µM, for 40 min (substrate consumption was less than 10%). Reactions were terminated and analysed using the UDP-Glo™ kit, adhering to the manufacturer's protocol. The experiment was repeated three times on the same day.

For protein thermal stability, purified OGT (323-1014 aa) or its variants were prepared by diluting the protein to a final concentration of 1 mg/mL in a buffer containing 100 mM Tris-HCl, 200 mM NaCl, and 0.5 mM TCEP. This solution was then mixed with SYPRO Orange Protein Gel Stain (Sigma-Aldrich) at a dilution of 1:5000. Subsequently, 25 µL of the assay samples were subjected to testing and analysis as previously described[8].

Supplemental references

1. Robert, X., and Gouet, P. (2014). Deciphering key features in protein structures with the new ENDscript server. Nucleic Acids Res. *42*, W320–W324. https://doi.org/10.1093/nar/gku316.

2. Chen, S., Francioli, L.C., Goodrich, J.K., Collins, R.L., Kanai, M., Wang, Q., Alföldi, J., Watts, N.A., Vittal, C., Gauthier, L.D., et al. (2024). A genomic mutational constraint map using variation in 76,156 human genomes. Nature *625*, 92–100. https://doi.org/10.1038/s41586-023-06045-0.

3. Tamm, C., Kadekar, S., Pijuan-Galitó, S., and Annerén, C. (2016). Fast and Efficient Transfection of Mouse Embryonic Stem Cells Using Non-Viral Reagents. Stem Cell Rev. Rep. *12*, 584–591. https://doi.org/10.1007/s12015-016-9673-5.

4. Pravata, V.M., Muha, V., Gundogdu, M., Ferenbach, A.T., Kakade, P.S., Vandadi, V., Wilmes, A.C., Borodkin, V.S., Joss, S., Stavridis, M.P., et al. (2019). Catalytic deficiency of O-GlcNAc transferase leads to X-linked intellectual disability. Proc. Natl. Acad. Sci. U. S. A. *116*, 14961–14970. https://doi.org/10.1073/pnas.1900065116.

5. Ying, Q.-L., Wray, J., Nichols, J., Batlle-Morera, L., Doble, B., Woodgett, J., Cohen, P., and Smith, A. (2008). The ground state of embryonic stem cell self-renewal. Nature *453*, 519–523. https://doi.org/10.1038/nature06968.

6. Shen, B., Zhang, W., Zhang, J., Zhou, J., Wang, J., Chen, L., Wang, L., Hodgkins, A., Iyer, V., Huang, X., et al. (2014). Efficient genome modification by CRISPR-Cas9 nickase with minimal off-target effects. Nat. Methods *11*, 399–402. https://doi.org/10.1038/nmeth.2857.

7. Pravata, V.M., Gundogdu, M., Bartual, S.G., Ferenbach, A.T., Stavridis, M., Õunap, K., Pajusalu, S., Žordania, R., Wojcik, M.H., and van Aalten, D.M.F. (2020). A missense mutation in the catalytic domain of O-GlcNAc transferase links perturbations in protein O-GlcNAcylation to X-linked intellectual disability. FEBS Lett. *594*, 717–727. https://doi.org/10.1002/1873-3468.13640.

8. Omelková, M., Fenger, C.D., Murray, M., Hammer, T.B., Pravata, V.M., Bartual, S.G., Czajewski, I., Bayat, A., Ferenbach, A.T., Stavridis, M.P., et al. (2023). An *O* -GlcNAc transferase pathogenic variant linked to intellectual disability affects pluripotent stem cell self-renewal. Dis. Model. Mech. *16*, dmm049132. https://doi.org/10.1242/dmm.049132.
