# Supplementary material for: Exploiting O-GlcNAc dyshomeostasis to screen O-GlcNAc transferase intellectual disability variants
Source: Stem Cell Reports. 2024 Dec 19;20(1):102380. doi: 10.1016/j.stemcr.2024.11.010 (PMC11784489; doi:10.1016/j.stemcr.2024.11.010)
Supplement: Document S2. Article plus supplemental information [file mmc2.pdf]

Stem Cell Reports

Article

OPEN ACCESS

Exploiting O-GlcNAc dyshomeostasis to screen O-GlcNAc transferase intellectual disability variants

Huijie Yuan,[1,2,3] Conor W. Mitchell,[1] Andrew T. Ferenbach,[1,2] Maria Teresa Bonati,[4] Agnese Feresin,[5] Paul J. Benke,[6] Queenie K.G. Tan,[7] and Daan M.F. van Aalten[1,2,3,8,*]

[1]Section for Neurobiology, Department of Molecular Biology and Genetics, Aarhus University, Aarhus, Denmark
[2]Danish Research Institute of Translational Neuroscience DANDRITE-Nordic EMBL Partnership for Molecular Medicine, Aarhus University, Aarhus, Denmark
[3]Division of Molecular, Cell and Developmental Biology, School of Life Sciences, University of Dundee, Dundee, UK
[4]Institute for Maternal and Child Health IRCCS Burlo Garofolo, Trieste, Italy
[5]Department of Medicine, Surgery and Health Sciences, University of Trieste, Trieste, Italy
[6]Joe DiMaggio Children's Hospital, Hollywood, FL, USA
[7]Department of Clinical Genomics, Mayo Clinic, Rochester, NY, USA
[8]Lead contact
*Correspondence: daan@mbg.au.dk
https://doi.org/10.1016/j.stemcr.2024.11.010

SUMMARY

O-GlcNAcylation is an essential protein modification catalyzed by O-GlcNAc transferase (OGT). Missense variants in OGT are linked to a novel intellectual disability syndrome known as OGT congenital disorder of glycosylation (OGT-CDG). The mechanisms by which OGT missense variants lead to this heterogeneous syndrome are not understood, and no unified method exists for dissecting pathogenic from non-pathogenic variants. Here, we develop a double-fluorescence strategy in mouse embryonic stem cells to measure disruption of O-GlcNAc homeostasis by quantifying the effects of variants on endogenous OGT expression. OGT-CDG variants generally elicited a lower feedback response than wild-type and Genome Aggregation Database (gnomAD) OGT variants. This approach was then used to dissect new putative OGT-CDG variants from pathogenic background variants in other disease-associated genes. Our work enables the prediction of pathogenicity for rapidly emerging *de novo* OGT-CDG variants and points to reduced disruption of O-GlcNAc homeostasis as a common mechanism underpinning OGT-CDG.

INTRODUCTION

O-GlcNAcylation, the modification of Ser/Thr hydroxyls with N-acetylglucosamine, is a widespread and conserved post-translational modification (PTM) occurring on over 9,000 nuclear, cytoplasmic, and mitochondrial proteins (Wulff-Fuentes et al., 2021). Unlike other PTMs, O-GlcNAcylation is regulated by just two enzymes. The O-GlcNAc transferase, OGT, composed of an N-terminal tetratricopeptide repeat (TPR) domain and a C-terminal glycosyltransferase (GT) domain, modifies Ser/Thr hydroxyls with GlcNAc (Haltiwanger et al., 1992) while the antagonistic enzyme, the O-GlcNAc hydrolase (OGA), removes O-GlcNAc (Dong and Hart, 1994). The OGT TPR domain forms a superhelical structure that mediates substrate binding and selectivity (Joiner et al., 2021).

Recently, missense variants in OGT have been reported in patients with a novel, syndromic form of X-linked intellectual disability (ID), called the OGT-linked congenital disorder of glycosylation (OGT-CDG) (Pravata et al., 2020a). OGT-CDG variants are present in the TPR and GT domains and lead to a wide range of facial dysmorphic, neurological, behavioral, and peripheral abnormalities. Multiple hypotheses have been proposed to explain how OGT-CDG variants affect OGT activity to cause disease, and various model systems including patient-derived lymphoblasts and skin fibroblasts, human and mouse embryonic stem cells (h/mESCs), *Drosophila melanogaster* and knockin mice have been generated to dissect the effects of OGT-CDG variants on OGT activity and identify dysregulated pathways (Authier et al., 2024; Omelková et al., 2023; Pravata et al., 2019; 2020b; Selvan et al., 2018; Vaidyanathan et al., 2017; Willems et al., 2017). These model systems, in combination with *in vitro* and structural characterization of OGT-CDG variants, have identified destabilizing effects of some TPR variants (Gundogdu et al., 2018; Vaidyanathan et al., 2017; Willems et al., 2017) and loss of catalytic activity of GT domain variants (Omelková et al., 2023; Pravata et al., 2019; 2020b), suggesting that a mixture of dosage and catalytic effects may contribute to disease etiology. However, differences in the dependencies of different cell lines and model organisms for O-GlcNAc and cell-type-specific regulatory mechanisms for the O-GlcNAc cycling enzymes, have complicated the identification of common biochemical characteristics/phenotypes between OGT-CDG variants. Additionally, the different genetic backgrounds of cell and model organisms may interact with the effects of a given OGT-CDG variant, calling into question the generality of any observed effects on O-GlcNAc homeostasis, cell signaling, or organism development. A salient example of this is the OGT A319T variant, which is present in a patient alongside a missense variant in the

Published by Elsevier Inc. on behalf of International Society for Stem Cell Research.
This is an open access article under the CC BY license (http://creativecommons.org/licenses/by/4.0/).

ID-associated gene *MED12* (Bouazzi et al., 2015). Consequently, no rapid screening method, exploiting a single isogenic background, is available for OGT-CDG, and putative disease variants identified by clinicians must be characterized *in vitro* and *in cellulo*, a costly and time-consuming process that delays the identification of *bona fide* OGT-CDG variants.

Here, we report the generation of an isogenic fluorescent reporter system for measuring the effects of OGT variants on O-GlcNAc homeostasis. Building on the previously described feedback mechanism linking elevated O-GlcNAc levels to reduced endogenous OGT protein levels (Slawson et al., 2005), we fluorescently tagged OGT in mESCs to assay the effects of transfected OGT variants on endogenous OGT levels as a readout of O-GlcNAc homeostasis. Flow cytometry (FC) revealed that while exogenous wild-type OGT markedly reduces endogenous OGT expression, a catalytically inactive OGT mutant does not. Non-pathogenic OGT variants from the general population (Genome Aggregation Database [gnomAD] variants) influence O-GlcNAc homeostasis similarly to wild-type OGT, whereas most OGT-CDG variants differ, showing limited capacity to disrupt O-GlcNAc homeostasis. We report three new OGT-CDG variants and dissect these from background variants in other disease genes, underscoring the potential pathogenic role of a segregating missense mutation in *LMNA*. Collectively, our findings suggest that a reduced ability to disrupt O-GlcNAc homeostasis is a feature that can be used to screen OGT-CDG variants.

RESULTS

Three unrelated patients affected by unclassified ID segregate with OGT missense variants

Proband 1 was the firstborn male of healthy, unrelated Caucasian parents with two younger sisters. He was born at 41 weeks via C-section due to breech presentation, at a weight of 2,680 g (3rd centile, small for gestational age), length of 48 cm (7th centile), and OCF of 33 cm (7th centile). Apgar scores were 5, 8, and 10 at 1, 5, and 10 min, respectively. Pregnancy complications included an echogenic intracardiac focus and an arachnoid cyst, with no invasive procedures performed. At birth, the proband had cyanosis due to bilateral asymmetric choanal stenosis, confirmed by maxillofacial computed tomography. Neonatal hypotonia and recurrent bronchiolitis were observed, along with patent foramen ovale and ductus arteriosus on echocardiogram. He had congenital VI cranial nerve paralysis leading to monolateral strabismus and hypermetropia, corrected by lenses. Developmentally, he experienced a global delay, sitting at 9 months, walking at 19 months, and delayed speech progression. At 3.5 years, he formed simple sentences with phonological issues, echolalia, and comprehension difficulties. WPPSI-III testing at 3 years and 4 months indicated borderline cognitive abilities (intelligence quotient 79). Motor skills, particularly manual dexterity and balance (M-ABC-2), were below average. MRI revealed no brain abnormalities. At 6 years, the proband showed poor gaze, sociability, and attention difficulties. Dysmorphic features included heavy eyebrows, narrow palpebral fissures, smooth philtrum, full lips, high-arched palate, clinodactyly, toe syndactyly, and a hyperpigmented dorsal spot. His height was 111 cm (13th centile) and COF 49.7 cm (8th centile), with a stocky build. Genetic testing revealed a wild-type FMR1 CGG expansion and trio-SNP array. Trio-WES identified the *OGT* (GenBank: NM_181672.3): c.1004A>G, p.(N335S) variant, inherited from his asymptomatic mother and maternal grandmother. His mother showed an unbalanced X chromosome inactivation pattern (93:07), while the grandmother's analysis was uninformative.

Proband 2 was a female child of healthy, unrelated parents of Eastern European ancestry, with an older sister and younger brother. She was born at 40 weeks via vaginal delivery after a pregnancy conceived via *in vitro* fertilization, complicated by suspected aortic coarctation, though postnatal echocardiogram was normal. Her birth weight was 3.1 kg (27th percentile), and length was 51 cm (75th percentile). Family history was notable for a paternal uncle with schizophrenia, with no other neurodevelopmental disorders. In infancy, the proband exhibited clenched fists and delayed fine motor skills, particularly with her pincer grasp at 9 months. She met early motor milestones, sitting at 6 months and standing at 10 months, but walked independently only at 21 months. Speech development stalled after her first word at 12 months, with regression in both speech and motor skills, increased drooling, and a return to soft foods. Electroencephalogram results were normal. Developmental testing at 33 months revealed motor skills equivalent to an 18–20 month old, with a developmental quotient of 79 (Mullen Scales of Early Learning). She did not meet the criteria for autism spectrum disorder. At almost 4 years, she babbled, used 4–30 word approximations, and signed 2–3 words. She walked with inverted feet and ran but often fell, scribbled, and used utensils, though her cognitive processing was slow. She displayed sociable behavior, being overly friendly with strangers, and had occasional tantrums. Intermittent right eye esotropia was noted since 1 year of age, along with adenoid hypertrophy and frequent strep infections. Genetic testing via trio-WES identified a *de novo OGT* variant (c.2542A>T, p.(N848Y)) and a *de novo LMTK3* variant (c.1391G>A, p.(W464*)). At 3 years and 6 months, her height was 88 cm (8th percentile), weight 12.2 kg (13th percentile), and head circumference 46 cm (2nd percentile). Physical

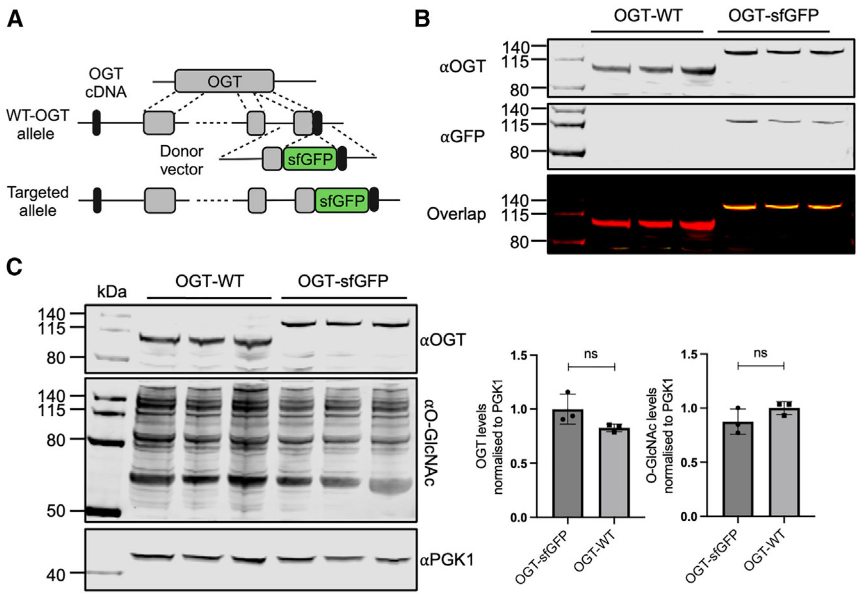

Figure 1. Fusion of sfGFP to endogenous OGT does not disrupt O-GlcNAc homeostasis
(A) Schematic of the CRISPR knockin strategy used to fuse sfGFP to the C terminus of endogenous *Ogt* in mESCs.
(B) Immunoblot of proteins extracted from CRISPR-engineered OGT-sfGFP and untreated wild-type mESCs, using antibodies against OGT and GFP (see also Figure S1).
(C) Left: OGT and O-GlcNAc (RL2) levels in OGT-sfGFP mESCs compared to untreated wild-type mESCs, with PGK1 as a loading control. Right: O-GlcNAc and OGT levels normalized to PGK1 (n = 3 independent experiments). Error bars represent the standard error of the mean (SEM). p values (unpaired t test): OGT = 0.10, O-GlcNAc = 0.17.

examination at 46 months revealed epicanthal folds, prominent infra-orbital folds, anteverted nares, hypotonia, brisk symmetric patellar reflexes, and downgoing Babinski toes, with no digit anomalies.

Proband 3 was a male evaluated at age 9 for cardiomyopathy present since birth and autism features that emerged between ages 2 and 3. He experienced failure to thrive until age 4, requiring breastfeeding and later a pureed food diet. Speech development was delayed, beginning at age 4. He exhibited hand flapping, inconsistent eye contact, and frequent tantrums, leading to an autism diagnosis. Genetic testing revealed two variants of uncertain significance (VUSs): *LMNA* c.1634 G>A, p.(R545H), initially linked to cardiomyopathy but later reclassified as non-pathogenic, and *OGT* c.3040 A>G, p.(M1014V). At age 9, his height was at the 18th percentile and weight at the 3rd percentile. He presented with a mild prominent forehead, flexible joints, a cardiac murmur, decreased muscle mass, fair eye contact, and normal speech and comprehension. Cardiac findings were stable, and speech had significantly improved. He performed at grade level with mild delays in reading and math. While hyperactivity persisted, his autism features had greatly improved, and attention-deficit/hyperactivity disorder became his primary neurodevelopmental diagnosis.

Patients' clinical phenotypes are summarized in Table S1.

Fusion of sfGFP to endogenous OGT does not disrupt O-GlcNAc homeostasis

Novel OGT missense variants segregating with ID are typically classified as VUS. Traditional classification requires extensive lab work, and we sought to establish a rapid, high-throughput screening method to identify pathogenic OGT-CDG variants. Given the feedback regulation between OGT, OGA, and global O-GlcNAc levels (Decourcelle et al., 2020), we hypothesized that monitoring OGT levels would offer a reliable readout of O-GlcNAc dyshomeostasis induced by OGT-CDG variants. The super folder variant of green fluorescent protein (sfGFP) (Pédelacq et al., 2006) was fused to the C terminus of endogenous *Ogt* in male mESCs using CRISPR genome editing (Figure 1A). Immunoblotting for OGT showed the expected molecular weight shift of OGT-sfGFP in edited mESCs compared to untreated wild-type controls, and immunoblotting for GFP revealed overlapping signals for OGT and GFP in OGT-sfGFP cells (Figure 1B). No significant changes in O-GlcNAc or OGT levels were observed in the edited OGT-sfGFP mESCs (Figure 1C). Interestingly, only the full-length OGT isoform was observed in these cells (Figure S1). Taken together, these data indicate that sfGFP fusion to endogenous OGT does not disrupt O-GlcNAc homeostasis.

OGT-sfGFP fluorescence responds to the pharmacological disruption of O-GlcNAc homeostasis

To assess whether OGT levels in OGT-sfGFP mESCs provide a readout of O-GlcNAc homeostasis, we modulated O-GlcNAc levels using inhibitors of OGA (10 μM Thiamet G [TMG] [Yuzwa et al., 2008]) or OGT (10 μM OSMI-4b [Martin et al., 2018]). As expected, immunoblotting revealed that OSMI-4b treatment decreased O-GlcNAc levels but increased OGT-sfGFP protein levels, whereas TMG treatment increased O-GlcNAc levels but reduced OGT-sfGFP protein levels (Figure 2A). Concomitantly, FC analysis revealed that treatment with OSMI-4b increased sfGFP

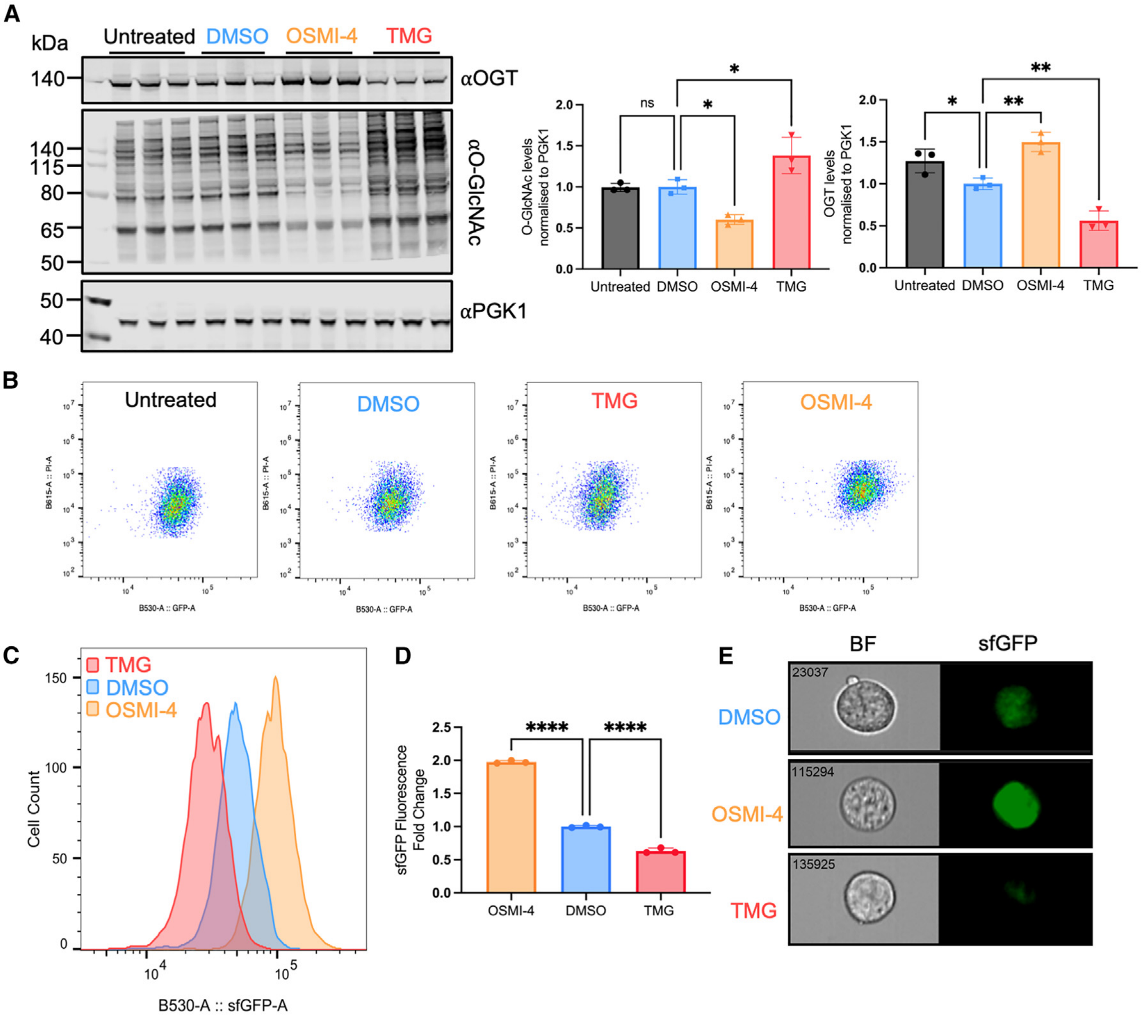

Figure 2. OGT-sfGFP levels/fluorescence respond to the pharmacological disruption of O-GlcNAc homeostasis

(A) Immunoblot showing OGT and O-GlcNAc (RL2) levels in OGT-sfGFP mESCs after 24-h treatment with 10 μM OSMI-4b or TMG, alongside a 0.1% DMSO vehicle control and an untreated control. PGK1 served as a loading control. Statistical analysis was performed on the right using urdinary one-way ANOVA (n = 3 independent replicates), with error bars representing mean ± SEM.

(B) Density plots of gated live singlet OGT-sfGFP mESCs after 10 μM OSMI-4b or TMG treatments (see gating strategy in the no-transfection section of Figure S2). OGT-sfGFP fluorescence is displayed on the x axis, and propidium iodide (PI) fluorescence on the y axis.

(C) Overlay of sfGFP histograms showing gated live singlet OGT-sfGFP mESCs after 10 μM OSMI-4b or TMG treatments, with OGT-sfGFP fluorescence on the x axis and cell count on the y axis.

(D) Median sfGFP fluorescence values from the sfGFP histogram (Figure 2C) were extracted for each sample and subjected to statistical analysis via ordinary one-way ANOVA (n = 3 independent replicates), with error bars indicating mean ± SEM.

(E) Images of OGT-sfGFP mESCs after 10 μM OSMI-4b or TMG treatments, captured in both bright-field (BF) and sfGFP fluorescence channels using an ImageStream flow cytometer. Statistical significance for all experiments is denoted as follows: * for adjusted $p < 0.05$, ** for $p < 0.01$, **** for $p < 0.0001$, and ns for $p > 0.05$.

fluorescence, whereas TMG treatment resulted in decreased fluorescence (Figure 2B, gating strategy provided in the no-transfection section in Figure S2). Clear separations in cell populations were observed in the sfGFP histogram (Figure 2C), showing nearly a 2-fold increase in median sfGFP fluorescence with OSMI-4b treatment and an approximately two-fifths reduction after TMG treatment (Figure 2D). Single-cell images captured during FC corroborate these changes (Figure 2E). FC enables high-throughput analysis of OGT-sfGFP levels with larger sample sizes and decreased variability across different cell passages compared to immunoblotting (Figures 2D and 2A). Collectively, these data show that OGT-sfGFP fluorescence responds to the pharmacological disruption of O-GlcNAc

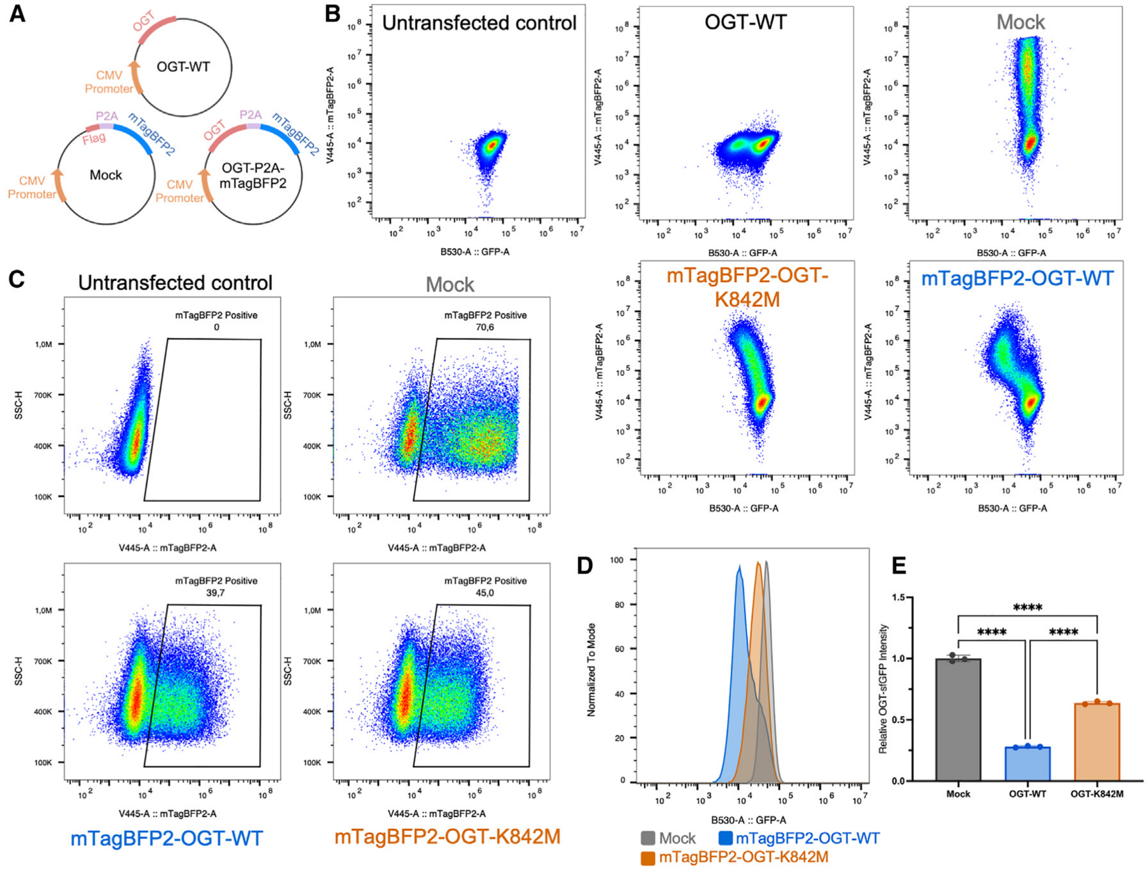

Figure 3. Endogenous OGT-sfGFP is a readout for the activity of exogenous OGT variants

(A) Illustration of plasmids used in the study: a wild-type OGT plasmid without fluorescent labeling (OGT-WT), a mock control plasmid with a FLAG tag linked to mTagBFP2 via the P2A linker, and mTagBFP2-OGT plasmids, where OGT or its variants are linked to mTagBFP2 via the P2A linker.

(B) Density plots of gated live singlet OGT-sfGFP mESCs after transfection with the unlabeled OGT-WT plasmid, fluorescently labeled mTagBFP2-OGT-K842M or mTagBFP2-OGT-WT plasmids, and the mock control. The x axis shows OGT-sfGFP fluorescence intensity, and the y axis displays mTagBFP2 fluorescence.

(C) Selection of transfected mTagBFP2$^+$ cells. A universal gate was applied across all samples to select OGT-sfGFP cells expressing mTagBFP2 (see plasmid-transfection section in Figure S2), with untransfected cells used as a control to define the gate.

(D) Overlay of sfGFP histograms for gated mTagBFP2$^+$ cells. Clear separations in sfGFP fluorescence were observed between cells transfected with mTagBFP2-OGT-WT, mTagBFP2-OGT-K842M, and the mock control plasmids.

(E) Statistical analysis of OGT-sfGFP fluorescence in gated mTagBFP2$^+$ mESCs following overexpression of mTagBFP2-OGT-WT, mTagBFP2-OGT-K842M, and the mock control. Each data point represents the median sfGFP fluorescence for the corresponding mTagBFP2$^+$ cells. Ordinary one-way ANOVA was performed (n = 3 independent replicates); **** denotes an adjusted p value <0.0001. Error bars represent mean ± SEM.

homeostasis, consistent with the established feedback regulation of O-GlcNAc.

Endogenous OGT-sfGFP is a readout for the activity of exogenous OGT variants

Given that endogenous OGT-sfGFP levels were sensitive to pharmacological perturbations of O-GlcNAc levels, we next investigated whether transfection of exogenous OGT variants would affect endogenous OGT-sfGFP fluorescence through O-GlcNAc feedback regulation. To enable gating for transfected cells, we constructed plasmids (Figure 3A) where OGT is fused to fluorescent marker mTagBFP2 (Subach et al., 2011) via a porcine teschovirus-1 self-cleaving 2A peptide (P2A) linker (Kim et al., 2011). The P2A linker enables the translation of separate OGT and mTagBFP2 proteins from a single mRNA strand through ribosome skipping (Kim et al., 2011), generating stochiometric expression of both proteins.

To explore this system, a fluorescently labeled wild-type OGT plasmid (mTagBFP2-OGT-WT) and a catalytically

inactive OGT mutant, mTagBFP2-OGT-K842M (Selvan et al., 2015) (Figure 3A), were transfected into OGT-sfGFP mESCs. Wild-type OGT plasmid, lacking the mTagBFP2 fluorescent marker (OGT-WT), and a plasmid encoding only mTagBFP2 (Mock) were used as controls (Figure 3A). OGT-sfGFP mESCs were analyzed by FC 48 h post-transfection. Density maps of gated live cells revealed relationships between sfGFP and mTagBFP2 fluorescence (Figure 3B). Compared to the untransfected control, cells transfected with the unlabeled OGT-WT plasmid showed a subset of cells with reduced OGT-sfGFP fluorescence, indicating downregulation of endogenous OGT-sfGFP following exogenous OGT expression (Figure 3B). The mock plasmid (Figure 3A), encoding only mTagBFP2, exhibited increased mTagBFP2 fluorescence without observable changes in sfGFP fluorescence (Figure 3B), suggesting no disturbance of O-GlcNAc homeostasis. Conversely, mTagBFP2-OGT-WT transfection led to increased mTagBFP2 fluorescence and decreased OGT-sfGFP levels (Figure 3B), further indicating endogenous OGT downregulation in response to exogenous OGT expression. Notably, cells transfected with the catalytic inactive mutant mTagBFP2-OGT-K842M showed only a mild decrease in OGT-sfGFP fluorescence despite high mTagBFP2 levels (Figure 3B), suggesting the feedback regulation may also respond to non-catalytic functions of OGT.

To quantify changes in OGT-sfGFP fluorescence due to exogenous OGT overexpression, a universal gate was employed to select cells with higher mTagBFP2 fluorescence than the untransfected control (Figure 3C; see Figure S2 for gating strategy). These selected mTagBFP2$^+$ cells were then presented as an sfGFP histogram (Figure 3D), with median OGT-sfGFP fluorescence used for statistical analysis (Figure 3E), revealing separation of mTagBFP2$^+$ cells transfected with mTagBFP2-OGT-WT, mTagBFP2-OGT-K842M, and the mock control (Figure 3D). Cells transfected with mTagBFP2-OGT-WT displayed approximately one-quarter of the OGT-sfGFP fluorescence intensity of the mock control, while mTagBFP2-OGT-K842M-transfected cells retained around three-quarters of the OGT-sfGFP fluorescence intensity (Figure 3E). Taken together, these data demonstrate that endogenous OGT-sfGFP provides a reliable readout for exogenous OGT variant activity and that O-GlcNAc feedback regulation correlates with the activity of the transfected OGT variant.

Non-pathogenic OGT variants disrupt O-GlcNAc homeostasis similarly to wild-type OGT

To evaluate the system's ability to dissecting pathogenic from non-pathogenic OGT variants, we first investigated the eight most common OGT variants in the general population, sourced from gnomAD v.4.0.0 (Chen et al., 2024). These variants span the TPR and GT domains of OGT (Figure 4A), with allele counts ranging from 115 to 21 among 730,947 exome sequences (Table S2). Notably, the original amino acids at these variant sites are generally not evolutionarily conserved, except for Leu279 (Figure S3). Each variant has been observed in hemizygous individuals, with homozygosity documented only for the D495E variant. Given their relative prevalence in the general population, these gnomAD variants are not expected to affect OGT activity. Indeed, when introducing these variants into OGT-sfGFP mESCs, the gated mTagBFP2$^+$ cells exhibited reduction in OGT-sfGFP fluorescence comparable to that observed with wild-type OGT transfection (Figure 4B). In all experiments, the catalytically inactive mTagBFP2-OGT-K842M mutant was used as a control to normalize OGT-sfGFP fluorescence in the transfected mTagBFP2$^+$ cells. Overall, these findings demonstrate that in our mESC transfection approach, non-pathogenic gnomAD OGT variants similarly disrupt O-GlcNAc homeostasis as wild-type OGT, eliciting O-GlcNAc feedback response to a comparable level.

Changes in OGT-sfGFP fluorescence predict OGT-CDG variant pathogenicity

The sensitivity of OGT-sfGFP fluorescence to transfected exogenous OGT plasmids suggests that OGT-CDG variants could provoke a less pronounced feedback regulation than non-pathogenic OGT variants. To test this, all currently reported pathogenic OGT-CDG variants were cloned (Omelková et al., 2023; Pravata et al., 2019, 2020b; Selvan et al., 2018; Vaidyanathan et al., 2017; Willems et al., 2017), each labeled with mTagBFP2 via the P2A linker (Figure 4A). These plasmids were transfected into OGT-sfGFP mESCs and analyzed by FC to examine OGT-sfGFP levels in mTagBFP2$^+$ cells as described earlier.

Interestingly, all reported pathogenic OGT-CDG variants in the GT domain produced a significantly smaller reduction in OGT-sfGFP fluorescence than wild-type OGT (Figure 4C), suggesting that these variants are either less active or more unstable, leading to weaker disruption of O-GlcNAc homeostasis. Nevertheless, the reduction was still more substantial than the response elicited by the inactive K842M OGT mutant (Figure 4C), which serves as a baseline to normalize OGT-sfGFP fluorescence in each transfection. This aligns with previous research supporting the pathogenic nature of these OGT-CDG variants. Strikingly, certain variants in the TPR domain, including the previously reported R284P OGT-CDG variant (Willems et al., 2017), the newly identified N335S putative OGT-CDG variant, and the Y180C variant (unpublished data), exhibit similar effects to OGT-CDG variants in the catalytic domain, with an attenuated reduction in endogenous OGT-sfGFP fluorescence after

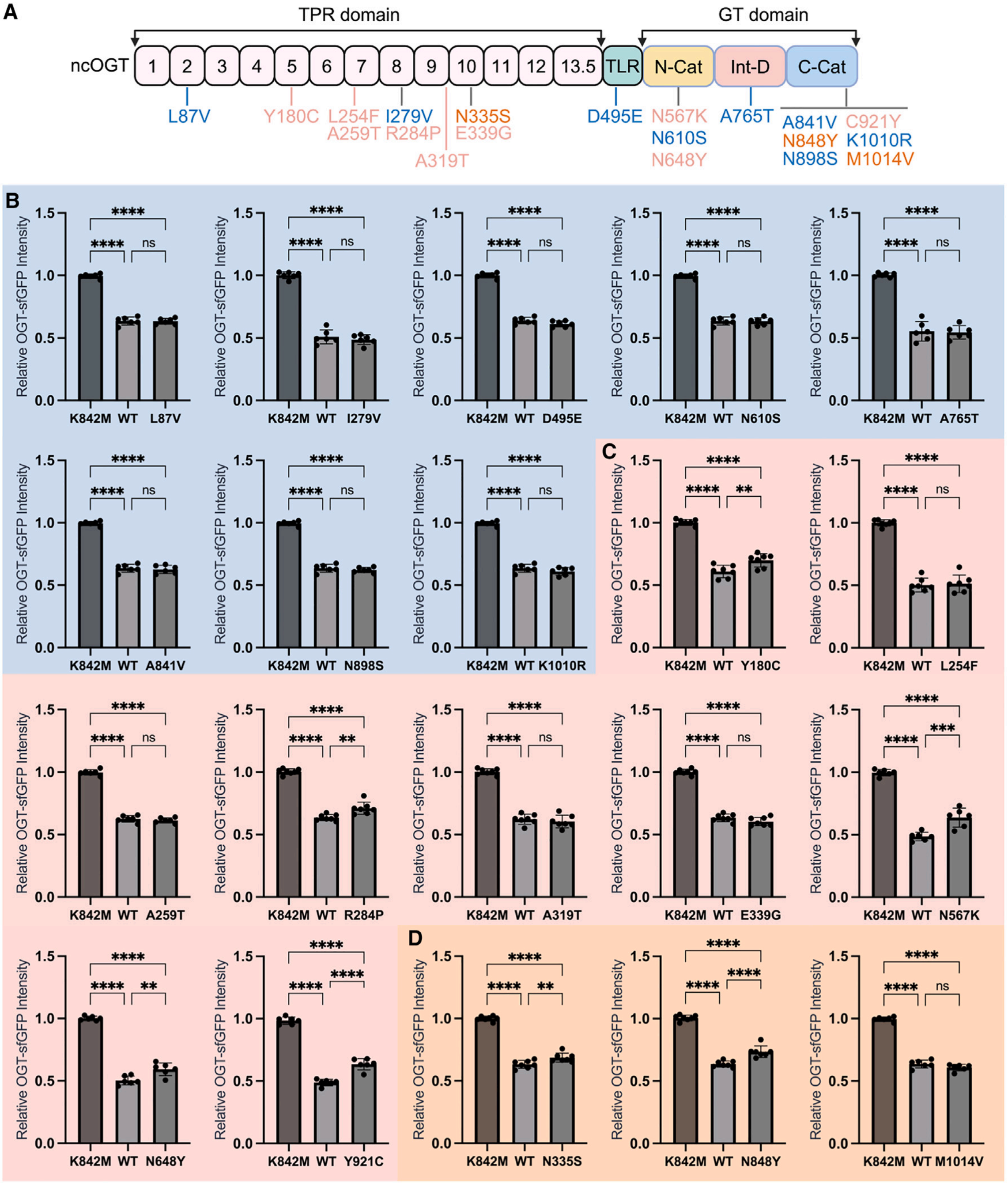

Figure 4. Changes in OGT-sfGFP fluorescence predict OGT-CDG variant pathogenicity

(A) Schematic of all OGT variants used in this study. Variants in blue represent the eight most frequent OGT variants from the gnomAD database (gnomAD variants; Table S2). Variants in pink are previously reported pathogenic OGT-CDG variants, while those in orange are three newly identified ID-associated OGT variants reported in this study. All variants are tagged with mTagBFP2 via the P2A linker for transfection.
(B–D) Statistical analysis of OGT-sfGFP fluorescence in transfected mTagBFP2$^+$ cells. Each OGT variant was transfected into OGT-sfGFP mESCs at least six times across different passages and days. Each data point represents the median OGT-sfGFP fluorescence from an independent transfection sample, normalized to the median fluorescence of the K842M mutant transfected sample. Analysis of gnomAD variants is shown in (B), previously reported pathogenic OGT-CDG variants in (C), and the three newly identified potential OGT-CDG variants in (D). Error bars represent mean ± SEM ($n \geq 6$). Ordinary one-way ANOVA was performed for each graph, with symbols indicating significance: ** for adjusted $p < 0.01$, **** for $p < 0.0001$, and ns for $p > 0.05$. A panel of representative density plots for mTagBFP2$^+$ cell selection across all transfected variants is presented in Figure S4.

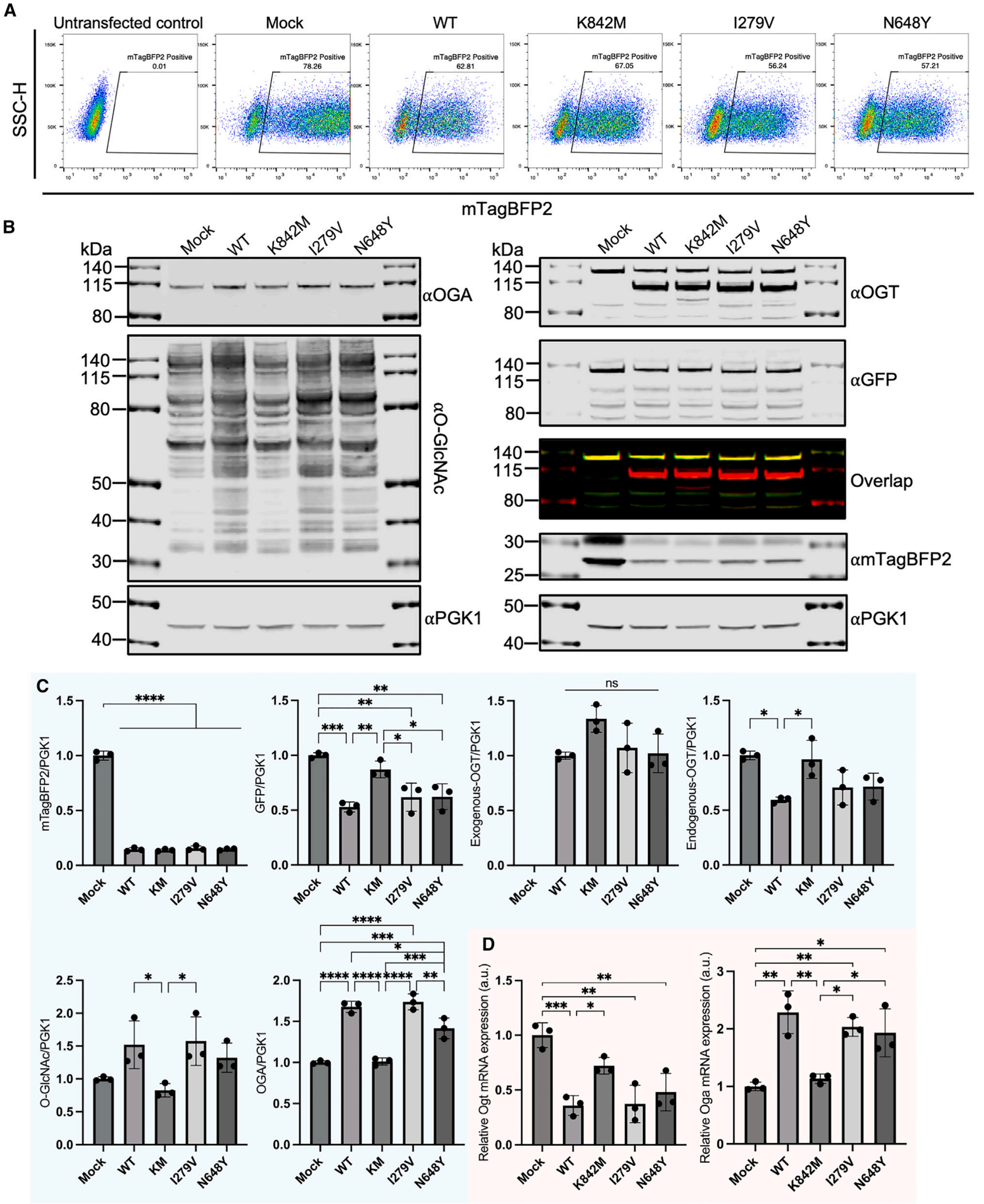

(legend on next page)

transfection (Figures 4C and 4D). However, other OGT-CDG TPR variants (L254F, A259T, A319T, E339G; Figure 4A) behaved similarly to the wild type in OGT-sfGFP mESCs (Figure 4C).

To validate the FC findings, we transfected OGT-sfGFP mESCs with a subset of plasmids, including OGT wild type, the catalytically inactive OGT-K842M mutant, the gnomAD OGT-I279V variant (Table S2), the pathogenic OGT-CDG N648Y variant (Pravata et al., 2020b), and the mock control (Figure 3A). Transfected mTagBFP2$^+$ cells were sorted (Figure 5A) and analyzed by immunoblotting (Figures 5B and 5C) and quantitative reverse-transcription PCR (RT-qPCR) (Figure 5D). Immunoblotting revealed that while the mock control had significantly higher mTagBFP2 expression, all other samples showed comparable expression levels, with no significant difference in exogenous OGT expression (Figures 5B and 5C, Note S1). These results suggest that variations in O-GlcNAc feedback regulation observed in FC were unlikely due to differences in plasmid transfection efficiency. A significant reduction in GFP levels was observed across all samples except K842M (Figures 5B and 5C). Similar trends were observed with endogenous OGT protein and O-GlcNAc levels, although the reduction in endogenous OGT protein and the increase in O-GlcNAc were not always statistically significant (Figures 5B and 5C). Nevertheless, these results align with the reduction in sfGFP fluorescence from FC analysis, highlighting the increased sensitivity of our assay compared to immunoblotting. Interestingly, OGA protein levels showed minimal variability between replicates, with a statistically significant increase in all transfected samples except K842M (Figures 5B and 5C). Notably, the N648Y OGT-CDG variant displayed a less pronounced increase in OGA levels than the wild-type and gnomAD I279V variant (Figure 5C). Compared to the mock control, overexpression of wild-type OGT, the gnomAD I279V variant, and the N648Y OGT-CDG variant resulted in significant reductions in *Ogt* mRNA levels and increases in *Oga* mRNA levels (Figure 5D). Overall, the immunoblotting and RT-qPCR data align with the FC results, collectively demonstrating that changes in OGT-sfGFP fluorescence correlate with the pathogenicity of OGT-CDG variants.

Changes in OGT-sfGFP fluorescence dissect new OGT-CDG cases from background genetic variants

A key challenge in predicting OGT-CDG variant pathogenicity is co-segregation with variants in other genes linked to ID, such as the A319T OGT-CDG variant (Selvan et al., 2018). We sought to determine whether the variant screening method described here could dissect OGT-CDG variants from such complex genetic backgrounds. Two of the new putative OGT-CDG cases described here (Table S1) possess additional variants in disease-associated genes. The OGT-N848Y variant co-occurs with a nonsense mutation in LMTK3, a brain tyrosine kinase (Kawa et al., 2004; Tomomura et al., 2007) implicated in NMDA receptor trafficking, with knockout models showing cognitive dysfunction and hyperactivity (Inoue et al., 2014; Montrose et al., 2019). For the M1014V OGT variant, a concurrent missense mutation was identified in *LMNA* (LMNA-R545H), a gene encoding A-type lamins associated with laminopathies and, in this case, ID (Worman and Bonne, 2007). Interestingly, cells transfected with the N848Y OGT induced reduced O-GlcNAc dyshomeostasis similar to previously identified pathogenic OGT-CDG variants (Figures 4C and 4D), whereas the M1014V OGT variant did not (Figure 4D), mirroring the effects observed in gnomAD variants (Figure 4B).

To further assess the effects of the N848Y and M1014V variants on OGT activity and stability, these variants were recombinantly produced in *E. coli* in a truncated form (Lazarus et al., 2011). In an assay testing catalytic activity against the well-characterized OGT substrate TAK1-binding protein 1 (TAB1, [Pathak et al., 2012]), the M1014V variant displayed wild-type activity, whereas the N848Y variant was catalytically impaired (35-fold decrease in catalytic efficiency; Figures S5A and S5B). Additionally, the N848Y variant showed reduced thermal stability ($\Delta T_m = 4°C$, Figure S5C), while the M1014V variant was unaffected (Figure S5C).

Overall, these findings indicate that the N848Y OGT variant is likely pathogenic, whereas the M1014V variant is likely not. Further investigation is required to clarify the potential contributions of the LMTK3 variant to pathology. Notably, the M1014V variant appears in the gnomAD database, recorded in one hemizygous and one

Figure 5. O-GlcNAc feedback regulation involves the transcriptional coordination of OGT and OGA
(A) Representative density plots for sorting mTagBFP2$^+$ cells following transfection with a subset of plasmids, including the mock control, wild-type OGT, the inactive K842M OGT mutant, the gnomAD I279V variant, and the pathogenic N648Y OGT-CDG variant. The sorted mTagBFP2$^+$ cells were then used for immunoblotting (B and C) and RT-qPCR (D) analysis.
(B) Representative western blots from lysates of sorted mTagBFP2$^+$ cells, blotted for mTagBFP2, GFP, OGT, O-GlcNAc (RL2), and OGA, with PGK1 as a loading control.
(C and D) Statistical analysis of western blot (C) and RT-qPCR (D) results. Ordinary one-way ANOVA was performed for all experiments ($n = 3$ independent replicates). Error bars represent mean ± SEM, with significance indicated as follows: * for adjusted $p < 0.05$, ** for $p < 0.01$, *** for $p < 0.001$, **** for $p < 0.0001$, and ns or no mark for non-significant results (adjusted $p > 0.05$).

heterozygous individual, and a related variant, M1014T, is reported in two additional heterozygous individuals. Conservation analysis shows that methionine at position 1,014 is not evolutionarily conserved (Figure S3), supporting that variants at this position may not substantially affect protein function. These observations further point to the link between LMNA-R545H and ID. Collectively, these data show that changes in endogenous OGT-sfGFP fluorescence effectively dissect *de novo* OGT-CDG variants from background genetic variants.

DISCUSSION

We have developed a double-fluorescence strategy to assess exogenous OGT variant activity by quantifying the intensity of O-GlcNAc feedback regulation. This approach allows the first comprehensive investigation of all currently identified pathogenic OGT-CDG variants within an isogenic cellular system. It reveals that most pathogenic variants disrupt O-GlcNAc homeostasis to a lesser degree than wild-type and non-pathogenic gnomAD OGT variants, failing to downregulate endogenous OGT to the same extent. This discovery highlights, for the first time, a unifying feature across most currently known pathogenic variants in a cellular context, suggesting that reduced disruption of O-GlcNAc homeostasis is a common characteristic of OGT-CDG variants.

Using transfection and FC, we introduce the first high-throughput method for predicting the pathogenicity of OGT variants associated with ID. With this approach, we evaluated three newly identified OGT variants in ID patients, identifying N335S and N848Y as likely pathogenic, while M1014V was likely benign. The conserved asparagine at position 335, located on the concave side of the TPR lumen, may play a role in substrate recognition (Joiner et al., 2021), although the exact mechanism by which this substitution affects O-GlcNAc homeostasis remains unclear. The benign nature of M1014V shifts attention to the LMNA-R545H variant, which has been associated with ID, showing incomplete penetrance and variable expressivity in other reports (Chan et al., 2016; Guillín-Amarelle et al., 2018; Magno et al., 2021; Patni et al., 2020). Further research is needed to understand how LMNA-R545H, either alone or in combination with M1014V, contributes to ID. Overall, this method aids in identifying a broader pool of pathogenic OGT-CDG variants for detailed biochemical investigations, ultimately advancing our understanding of OGT-CDG phenotype etiology.

Despite its utility, this screening method has limitations. Some OGT-CDG variants located in the TPR domain show disruptions in O-GlcNAc homeostasis similar to wild-type OGT. Notably, variants like A319T and L254F are found alongside mutations in other genes (e.g., *MED12, PRICKLE3*) (Bouazzi et al., 2015; Vaidyanathan et al., 2017). It is also conceivable that subtle changes in the activity or stability of these TPR domain OGT-CDG variants may not be detectable using the overexpression system described in this study. Additionally, the OGT interactome could play a role in OGT-CDG pathology (Stephen et al., 2021), which this assay does not assess. An alternative approach could include a fluorescence resonance energy transfer-based O-GlcNAc sensor that enables spatiotemporal detection of O-GlcNAc modifications (Carrillo et al., 2006; 2011), although this system relies on the O-GlcNAcylation of a single substrate.

Our study reveals the coordinated regulation of OGT and OGA at both mRNA and protein levels when exogenous OGT variants are overexpressed. Notably, the intensity of this regulation correlates with the activity of the variant being overexpressed, offering a new strategy to explore the mechanisms underlying O-GlcNAc feedback regulation. Current research suggests that this feedback regulation may involve multiple mechanisms at the transcriptional, post-transcriptional, and post-translational levels. For example, under hyper-O-GlcNAcylation conditions, OGT and OGA undergo co-transcriptional splicing to retain a highly conserved intron, targeting OGT mRNA for nonsense-mediated decay while promoting OGA mRNA for protein production (Park et al., 2017; Tan et al., 2020). Additionally, OGA mRNA translation is suppressed by micro-RNA539 following hypoxia-reoxygenation injury in cardiomyocytes (Muthusamy et al., 2015). OGT turnover is also regulated by phosphorylation at Ser20 by checkpoint kinase 1, reducing OGT flux toward the proteasome (Li et al., 2017). Overall, our study highlights that both OGT and OGA, at the transcriptional and protein levels, can serve as sensitive probes for detecting changes in O-GlcNAc homeostasis. This provides a robust system for screening drugs, genes, and signaling pathways involved in O-GlcNAc regulation to dissect the mechanisms underpinning O-GlcNAc homeostasis.

EXPERIMENTAL PROCEDURES

Ethics and consent

Informed consent for clinical testing and publication was obtained from the parents of all three probands, with approval from the relevant ethics committees (project number provided in supplemental experimental procedures).

Generation of OGT-sfGFP mES cell line

The mESCs used in this study were derived from E14-TG2a.IV (129/Ola) ES cells as previously described (Pravata et al., 2019). Details of CRISPR-Cas reagent designing, cloning procedures, and daily cell maintenance are provided in supplemental experimental procedures.

OSMI-4b and TMG treatments

OGT-sfGFP cells cultured in the GMEM-BHK 21 medium were treated with either 10 μM OSMI-4b or 10 μM TMG. All treatments were applied for 24 h, followed by either immunoblotting or FC analysis. Additional details are provided in the supplemental experimental procedures.

Protein extraction, western blot, and RT-qPCR

Details of the western blot, including protein extraction methods, antibody types, incubation times, and RT-qPCR procedures, are provided in the supplemental experimental procedures.

Transfection

For all transfection experiments, OGT-sfGFP mESCs were cultured in 2i medium (Ying et al., 2008). Transfection was carried out using Lipofectamine 2000 (Thermo Fisher Scientific), following the method reported before (Tamm et al., 2016) to enhance transfection efficiency. Cells were transfected for 48 h before follow-up analysis, with additional details provided in the supplemental experimental procedures.

FC analysis

OGT-sfGFP mESCs were analyzed by FC using the gating strategy shown in Figure S2, with additional details in the supplemental experimental procedures.

Enzyme assays and differential scanning fluorimetry

Wild-type and variant OGT protein (323–1,044 aa) were recombinantly purified from *E. coli* as described previously (Omelková et al., 2023). OGT and variants' activity against the substrate TAB1 was measured using either western blot or the UDP-Glo assay (Promega). OGT and variants' stability was measured by detecting protein melting temperatures. Extra details are provided in supplemental experimental procedures.

Statistics

The statistical analyses in this study were conducted using Prism version 10. The specific statistical methods applied are detailed in the legends accompanying each figure.

RESOURCE AVAILABILITY

Lead contact

Requests for further information and resources should be directed to and will be fulfilled by the corresponding author, Daan M. F. van Aalten (daan@mbg.au.dk).

Materials availability

All materials and reagents including the OGT-sfGFP mESC line generated in this study are available from the corresponding author upon request.

Data and code availability

Original western blot images and raw FC data reported in this study will be shared by the corresponding author upon request. Any additional information required to reanalyze the data reported in this paper is available from the corresponding author upon request.

ACKNOWLEDGMENTS

This work was funded by a Wellcome Trust Investigator Award (110061), a Novo Nordisk Fonden Laureate award (NNF21OC 0065969), and a Villum Fonden Investigator award (00054496) to D.M.F.v.A. H.Y. was funded by the China Scholarship Council. C.W.M. was funded by the BBSRC EASTBIO Doctoral Training Programme. This work was supported in part by the Danish Research Institute of Translational Neuroscience - DANDRITE of the Nordic-EMBL Partnership for Molecular Medicine and Lundbeckfonden. The authors thank the FACS Core Facility at Aarhus University and Dr Authier for their support. Parental informed consent was obtained for the participation of these patients, and their contribution is gratefully acknowledged.

AUTHOR CONTRIBUTIONS

H.Y., A.T.F., and D.M.F.v.A. conceived the study; H.Y. and C.W.M. performed experiments; A.T.F. performed molecular biology; H.Y. and D.M.F.v.A. analyzed data; and H.Y., C.W.M., and D.M.F.v.A. interpreted the data and wrote the manuscript with input from all authors. M.T.B., A.F., P.J.B., and Q.K.G.T. collected and compiled clinical data.

DECLARATION OF INTERESTS

The authors declare no competing interests.

DECLARATION OF GENERATIVE AI AND AI-ASSISTED TECHNOLOGIES IN THE WRITING PROCESS

During the preparation of this work, the authors used ChatGPT to check the grammar and refine the language to enhance conciseness. After using this tool, the authors reviewed and edited the content as needed and take full responsibility for the content of the publication.

SUPPLEMENTAL INFORMATION

Supplemental information can be found online at https://doi.org/10.1016/j.stemcr.2024.11.010.

Received: June 15, 2024
Revised: November 20, 2024
Accepted: November 21, 2024
Published: December 19, 2024

REFERENCES

Authier, F., Ondruskova, N., Ferenbach, A.T., McNeilly, A.D., and Van Aalten, D.M.F. (2024). Neurodevelopmental defects in a mouse model of O- GlcNAc transferase intellectual disability. Dis. Model. Mech. *17*, dmm050671. https://doi.org/10.1242/dmm.050671.

Bouazzi, H., Lesca, G., Trujillo, C., Alwasiyah, M.K., and Munnich, A. (2015). Nonsyndromic X-linked intellectual deficiency in three brothers with a novel MED12 missense mutation [c.5922G>T

(p.Glu1974His)]. Clin. Case Rep. *3*, 604–609. https://doi.org/10.1002/ccr3.301.

Carrillo, L.D., Krishnamoorthy, L., and Mahal, L.K. (2006). A Cellular FRET-Based Sensor for β-O-GlcNAc, A Dynamic Carbohydrate Modification Involved in Signaling. J. Am. Chem. Soc. *128*, 14768–14769. https://doi.org/10.1021/ja065835+.

Carrillo, L.D., Froemming, J.A., and Mahal, L.K. (2011). Targeted in Vivo O-GlcNAc Sensors Reveal Discrete Compartment-specific Dynamics during Signal Transduction. J. Biol. Chem. *286*, 6650–6658. https://doi.org/10.1074/jbc.M110.191627.

Chan, D., McIntyre, A.D., Hegele, R.A., and Don-Wauchope, A.C. (2016). Familial partial lipodystrophy presenting as metabolic syndrome. J. Clin. Lipidol. *10*, 1488–1491. https://doi.org/10.1016/j.jacl.2016.08.012.

Chen, S., Francioli, L.C., Goodrich, J.K., Collins, R.L., Kanai, M., Wang, Q., Alföldi, J., Watts, N.A., Vittal, C., Gauthier, L.D., et al. (2024). A genomic mutational constraint map using variation in 76,156 human genomes. Nature *625*, 92–100. https://doi.org/10.1038/s41586-023-06045-0.

Decourcelle, A., Loison, I., Baldini, S., Leprince, D., and Dehennaut, V. (2020). Evidence of a compensatory regulation of colonic O-GlcNAc transferase and O-GlcNAcase expression in response to disruption of O-GlcNAc homeostasis. Biochem. Biophys. Res. Commun. *521*, 125–130. https://doi.org/10.1016/j.bbrc.2019.10.090.

Dong, D.L., and Hart, G.W. (1994). Purification and characterization of an O-GlcNAc selective N-acetyl-beta-D-glucosaminidase from rat spleen cytosol. J. Biol. Chem. *269*, 19321–19330.

Guillín-Amarelle, C., Sánchez-Iglesias, S., Mera, A., Pintos, E., Castro-Pais, A., Rodríguez-Cañete, L., Pardo, J., Casanueva, F.F., and Araújo-Vilar, D. (2018). Inflammatory myopathy in the context of an unusual overlapping laminopathy. Archives of Endocrinology and Metabolism *62*, 376–382. https://doi.org/10.20945/2359-3997000000048.

Gundogdu, M., Llabrés, S., Gorelik, A., Ferenbach, A.T., Zachariae, U., and van Aalten, D.M.F. (2018). The O-GlcNAc Transferase Intellectual Disability Mutation L254F Distorts the TPR Helix. Cell Chem. Biol. *25*, 513–518.e4. https://doi.org/10.1016/j.chembiol.2018.03.004.

Haltiwanger, R.S., Blomberg, M.A., and Hart, G.W. (1992). Glycosylation of nuclear and cytoplasmic proteins. Purification and characterization of a uridine diphospho-N-acetylglucosamine:polypeptide beta-N-acetylglucosaminyltransferase. J. Biol. Chem. *267*, 9005–9013.

Inoue, T., Hoshina, N., Nakazawa, T., Kiyama, Y., Kobayashi, S., Abe, T., Yamamoto, T., Manabe, T., and Yamamoto, T. (2014). LMTK3 Deficiency Causes Pronounced Locomotor Hyperactivity and Impairs Endocytic Trafficking. J. Neurosci. *34*, 5927–5937. https://doi.org/10.1523/JNEUROSCI.1621-13.2014.

Joiner, C.M., Hammel, F.A., Janetzko, J., and Walker, S. (2021). Protein Substrates Engage the Lumen of O-GlcNAc Transferase's Tetratricopeptide Repeat Domain in Different Ways. Biochemistry *60*, 847–853. https://doi.org/10.1021/acs.biochem.0c00981.

Kawa, S., Fujimoto, J., Tezuka, T., Nakazawa, T., and Yamamoto, T. (2004). Involvement of BREK, a serine/threonine kinase enriched in brain, in NGF signalling. Gene Cell. *9*, 219–232. https://doi.org/10.1111/j.1356-9597.2004.00714.x.

Kim, J.H., Lee, S.-R., Li, L.-H., Park, H.-J., Park, J.-H., Lee, K.Y., Kim, M.-K., Shin, B.A., and Choi, S.-Y. (2011). High cleavage efficiency of a 2A peptide derived from porcine teschovirus-1 in human cell lines, zebrafish and mice. PLoS One *6*, e18556. https://doi.org/10.1371/journal.pone.0018556.

Lazarus, M.B., Nam, Y., Jiang, J., Sliz, P., and Walker, S. (2011). Structure of human O-GlcNAc transferase and its complex with a peptide substrate. Nature *469*, 564–567. https://doi.org/10.1038/nature09638.

Li, Z., Li, X., Nai, S., Geng, Q., Liao, J., Xu, X., and Li, J. (2017). Checkpoint kinase 1–induced phosphorylation of O-linked -N-acetylglucosamine transferase regulates the intermediate filament network during cytokinesis. J. Biol. Chem. *292*, 19548–19555. https://doi.org/10.1074/jbc.M117.811646.

Magno, S., Ceccarini, G., Barison, A., Fabiani, I., Giacomina, A., Gilio, D., Pelosini, C., Rubegni, A., Emdin, M., Gatti, G.L., et al. (2021). Partial Lipodystrophy and LMNA p.R545H Variant. JCM *10*, 1142. https://doi.org/10.3390/jcm10051142.

Martin, S.E.S., Tan, Z.-W., Itkonen, H.M., Duveau, D.Y., Paulo, J.A., Janetzko, J., Boutz, P.L., Törk, L., Moss, F.A., Thomas, C.J., et al. (2018). Structure-Based Evolution of Low Nanomolar O-GlcNAc Transferase Inhibitors. J. Am. Chem. Soc. *140*, 13542–13545. https://doi.org/10.1021/jacs.8b07328.

Montrose, K., Kobayashi, S., Manabe, T., and Yamamoto, T. (2019). Lmtk3-KO Mice Display a Range of Behavioral Abnormalities and Have an Impairment in GluA1 Trafficking. Neuroscience *414*, 154–167. https://doi.org/10.1016/j.neuroscience.2019.06.033.

Muthusamy, S., Hong, K.U., Dassanayaka, S., Hamid, T., and Jones, S.P. (2015). E2F1 Transcription Factor Regulates O-linked N-acetylglucosamine (O-GlcNAc) Transferase and O-GlcNAcase Expression. J. Biol. Chem. *290*, 31013–31024. https://doi.org/10.1074/jbc.M115.677534.

Omelková, M., Fenger, C.D., Murray, M., Hammer, T.B., Pravata, V.M., Bartual, S.G., Czajewski, I., Bayat, A., Ferenbach, A.T., Stavridis, M.P., et al. (2023). An O-GlcNAc transferase pathogenic variant linked to intellectual disability affects pluripotent stem cell self-renewal. Dis Model Mech *16*, dmm049132. https://doi.org/10.1242/dmm.049132.

Park, S.K., Zhou, X., Pendleton, K.E., Hunter, O.V., Kohler, J.J., O'Donnell, K.A., and Conrad, N.K. (2017). A Conserved Splicing Silencer Dynamically Regulates O-GlcNAc Transferase Intron Retention and O-GlcNAc Homeostasis. Cell Rep. *20*, 1088–1099. https://doi.org/10.1016/j.celrep.2017.07.017.

Pathak, S., Borodkin, V.S., Albarbarawi, O., Campbell, D.G., Ibrahim, A., and van Aalten, D.M. (2012). O-GlcNAcylation of TAB1 modulates TAK1-mediated cytokine release. EMBO J. *31*, 1394–1404. https://doi.org/10.1038/emboj.2012.8.

Patni, N., Hatab, S., Xing, C., Zhou, Z., Quittner, C., and Garg, A. (2020). A novel autosomal recessive lipodystrophy syndrome due to homozygous LMNA variant. J. Med. Genet. *57*, 422–426. https://doi.org/10.1136/jmedgenet-2019-106395.

Pédelacq, J.-D., Cabantous, S., Tran, T., Terwilliger, T.C., and Waldo, G.S. (2006). Engineering and characterization of a

superfolder green fluorescent protein. Nat. Biotechnol. *24*, 79–88. https://doi.org/10.1038/nbt1172.

Pravata, V.M., Muha, V., Gundogdu, M., Ferenbach, A.T., Kakade, P.S., Vandadi, V., Wilmes, A.C., Borodkin, V.S., Joss, S., Stavridis, M.P., and van Aalten, D.M.F. (2019). Catalytic deficiency of O-GlcNAc transferase leads to X-linked intellectual disability. Proc. Natl. Acad. Sci. USA *116*, 14961–14970. https://doi.org/10.1073/pnas.1900065116.

Pravata, V.M., Omelková, M., Stavridis, M.P., Desbiens, C.M., Stephen, H.M., Lefeber, D.J., Gecz, J., Gundogdu, M., Õunap, K., Joss, S., et al. (2020a). An intellectual disability syndrome with single-nucleotide variants in O-GlcNAc transferase. Eur. J. Hum. Genet. *28*, 706–714. https://doi.org/10.1038/s41431-020-0589-9.

Pravata, V.M., Gundogdu, M., Bartual, S.G., Ferenbach, A.T., Stavridis, M., Õunap, K., Pajusalu, S., Žordania, R., Wojcik, M.H., and van Aalten, D.M.F. (2020b). A missense mutation in the catalytic domain of O-GlcNAc transferase links perturbations in protein O-GlcNAcylation to X-linked intellectual disability. FEBS Lett. *594*, 717–727. https://doi.org/10.1002/1873-3468.13640.

Selvan, N., Mariappa, D., van den Toorn, H.W.P., Heck, A.J.R., Ferenbach, A.T., and van Aalten, D.M.F. (2015). The Early Metazoan Trichoplax adhaerens Possesses a Functional O-GlcNAc System. J. Biol. Chem. *290*, 11969–11982. https://doi.org/10.1074/jbc.M114.628750.

Selvan, N., George, S., Serajee, F.J., Shaw, M., Hobson, L., Kalscheuer, V., Prasad, N., Levy, S.E., Taylor, J., Aftimos, S., et al. (2018). O-GlcNAc transferase missense mutations linked to X-linked intellectual disability deregulate genes involved in cell fate determination and signaling. J. Biol. Chem. *293*, 10810–10824. https://doi.org/10.1074/jbc.RA118.002583.

Slawson, C., Zachara, N.E., Vosseller, K., Cheung, W.D., Lane, M.D., and Hart, G.W. (2005). Perturbations in O-linked β-N-Acetylglucosamine Protein Modification Cause Severe Defects in Mitotic Progression and Cytokinesis. J. Biol. Chem. *280*, 32944–32956. https://doi.org/10.1074/jbc.M503396200.

Stephen, H.M., Praissman, J.L., and Wells, L. (2021). Generation of an Interactome for the Tetratricopeptide Repeat Domain of O-GlcNAc Transferase Indicates a Role for the Enzyme in Intellectual Disability. J. Proteome Res. *20*, 1229–1242. https://doi.org/10.1021/acs.jproteome.0c00604.

Subach, O.M., Cranfill, P.J., Davidson, M.W., and Verkhusha, V.V. (2011). An Enhanced Monomeric Blue Fluorescent Protein with the High Chemical Stability of the Chromophore. PLoS One *6*, e28674. https://doi.org/10.1371/journal.pone.0028674.

Tamm, C., Kadekar, S., Pijuan-Galitó, S., and Annerén, C. (2016). Fast and Efficient Transfection of Mouse Embryonic Stem Cells Using Non-Viral Reagents. Stem Cell Rev. Rep. *12*, 584–591. https://doi.org/10.1007/s12015-016-9673-5.

Tan, Z.W., Fei, G., Paulo, J.A., Bellaousov, S., Martin, S.E.S., Duveau, D.Y., Thomas, C.J., Gygi, S.P., Boutz, P.L., and Walker, S. (2020). O-GlcNAc regulates gene expression by controlling detained intron splicing. Nucleic Acids Res. *48*, 5656–5669. https://doi.org/10.1093/NAR/GKAA263.

Tomomura, M., Morita, N., Yoshikawa, F., Konishi, A., Akiyama, H., Furuichi, T., and Kamiguchi, H. (2007). Structural and functional analysis of the apoptosis-associated tyrosine kinase (AATYK) family. Neuroscience *148*, 510–521. https://doi.org/10.1016/j.neuroscience.2007.05.048.

Vaidyanathan, K., Niranjan, T., Selvan, N., Teo, C.F., May, M., Patel, S., Weatherly, B., Skinner, C., Opitz, J., Carey, J., et al. (2017). Identification and characterization of a missense mutation in the O-linked β-N-acetylglucosamine (O-GlcNAc) transferase gene that segregates with X-linked intellectual disability. J. Biol. Chem. *292*, 8948–8963. https://doi.org/10.1074/jbc.M116.771030.

Willems, A.P., Gundogdu, M., Kempers, M.J.E., Giltay, J.C., Pfundt, R., Elferink, M., Loza, B.F., Fuijkschot, J., Ferenbach, A.T., Van Gassen, K.L.I., et al. (2017). Mutations in N-acetylglucosamine (O-GlcNAc) transferase in patients with X-linked intellectual disability. J. Biol. Chem. *292*, 12621–12631. https://doi.org/10.1074/jbc.M117.790097.

Worman, H.J., and Bonne, G. (2007). "Laminopathies": A wide spectrum of human diseases. Exp. Cell Res. *313*, 2121–2133. https://doi.org/10.1016/j.yexcr.2007.03.028.

Wulff-Fuentes, E., Berendt, R.R., Massman, L., Danner, L., Malard, F., Vora, J., Kahsay, R., and Olivier-Van Stichelen, S. (2021). The human O-GlcNAcome database and meta-analysis. Sci. Data *8*, 25. https://doi.org/10.1038/s41597-021-00810-4.

Ying, Q.-L., Wray, J., Nichols, J., Batlle-Morera, L., Doble, B., Woodgett, J., Cohen, P., and Smith, A. (2008). The ground state of embryonic stem cell self-renewal. Nature *453*, 519–523. https://doi.org/10.1038/nature06968.

Yuzwa, S.A., Macauley, M.S., Heinonen, J.E., Shan, X., Dennis, R.J., He, Y., Whitworth, G.E., Stubbs, K.A., McEachern, E.J., Davies, G.J., and Vocadlo, D.J. (2008). A potent mechanism-inspired O-GlcNAcase inhibitor that blocks phosphorylation of tau in vivo. Nat. Chem. Biol. *4*, 483–490. https://doi.org/10.1038/nchembio.96.

Stem Cell Reports, Volume *20*

Supplemental Information

Exploiting O-GlcNAc dyshomeostasis to screen O-GlcNAc transferase intellectual disability variants

Huijie Yuan, Conor W. Mitchell, Andrew T. Ferenbach, Maria Teresa Bonati, Agnese Feresin, Paul J. Benke, Queenie K.G. Tan, and Daan M.F. van Aalten

Figure S1. Full-length OGT isoform was labelled with sfGFP, related to Figure 1.

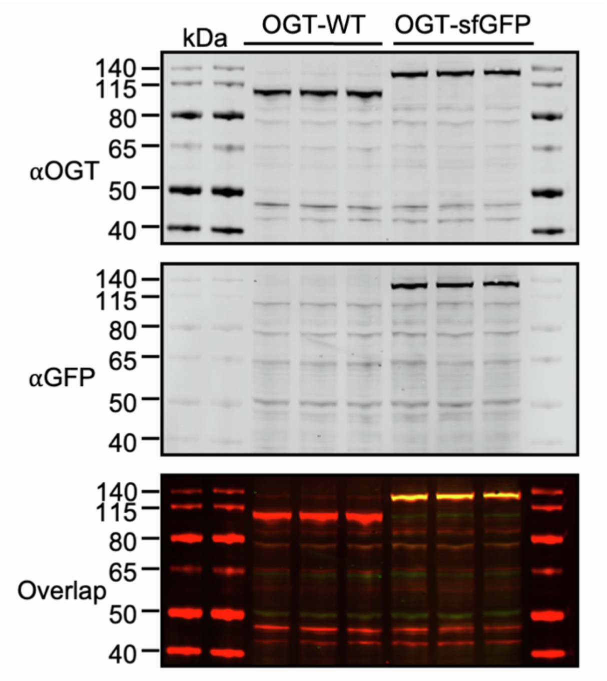

Cell lysates extracted from CRISPR-engineered OGT-sfGFP mESCs and untreated wild type mESCs were subjected to immunoblotting using antibodies against OGT and GFP. OGT was visualised in red, and GFP was visualised in green, with overlapping signals appearing as yellow.

Figure S2. Gating strategy for flow cytometry data analysis, related to Figures 2-5.

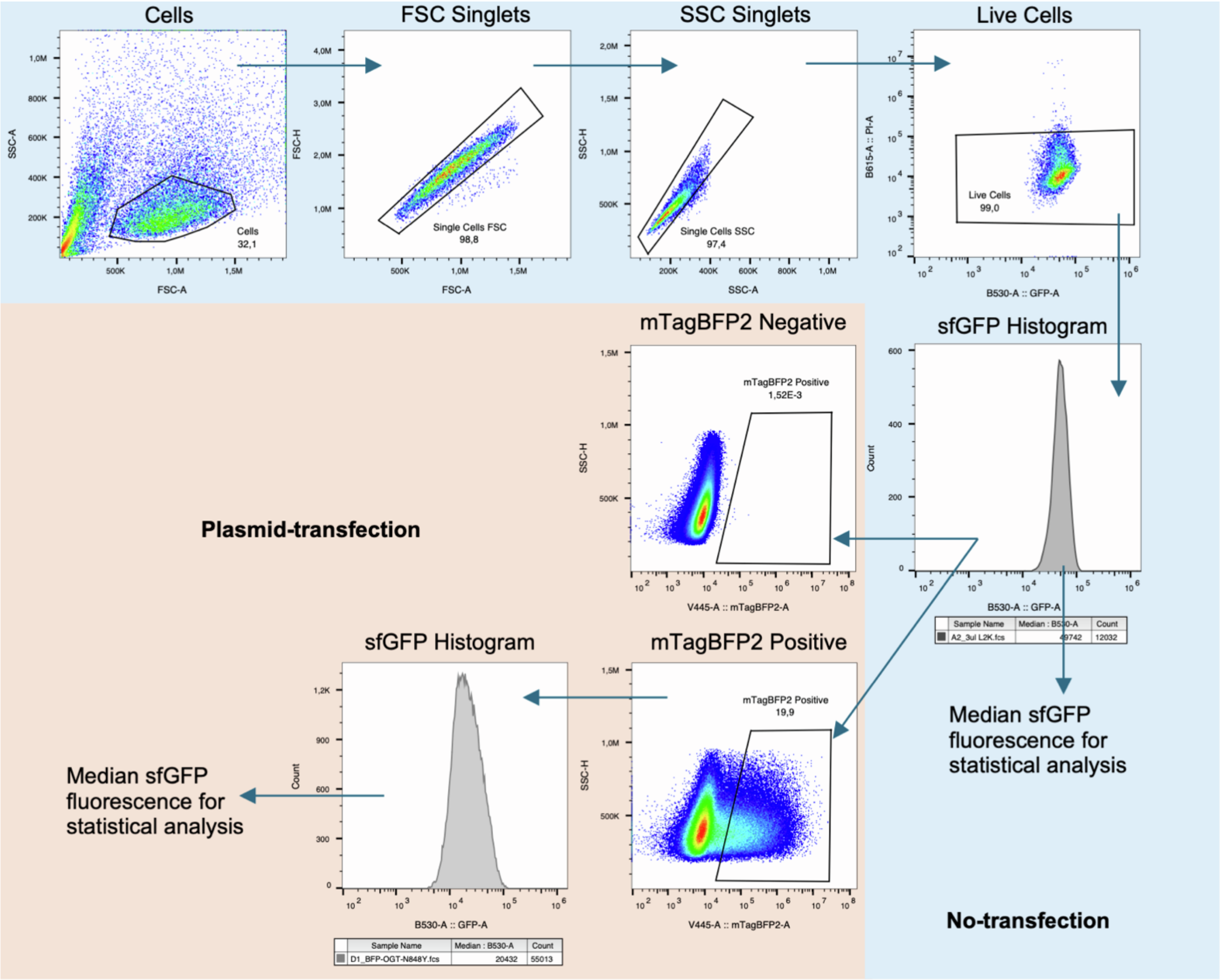

All flow cytometry data were analysed based on this gating strategy. For no-transfection experiments (coloured in light blue), samples were first visualized using forward scatter (FSC) and side scatter (SSC) to gate cells based on size and granularity. These gated cells were then further analysed by comparing FSC height (FSC-H) with FSC area (FSC-A), and SSC height (SSC-H) with SSC area (SSC-A), to isolate cell singlets. The isolated single cells were subsequently plotted on a propidium iodide area (PI-A) versus GFP area (GFP-A) graph to identify live cells. A histogram of sfGFP fluorescence in gated live singlet cells was generated, and the median fluorescence value was extracted for statistical analysis.

For cells transfected with mTagBFP2 labelled plasmids (coloured in light orange), the same procedures were followed with additional steps to select mTagBFP2 positive (mTagBFP2^{+}) cells from the gated live singlet OGT-sfGFP mESCs. A gate was defined based on mTagBFP2 expression, dividing the cells into two groups: mTagBFP2^{+}, indicating successful transfection and expression, and mTagBFP2 negative (mTagBFP2^{-}), indicating no expression of mTagBFP2. The gated mTagBFP2^{+} cells were then visualized in the sfGFP histogram, and the median OGT-sfGFP fluorescence value was extracted for statistical analysis.

Figure S3. Pathogenic and non-pathogenic OGT variants conservation, related to Figures 4-5.

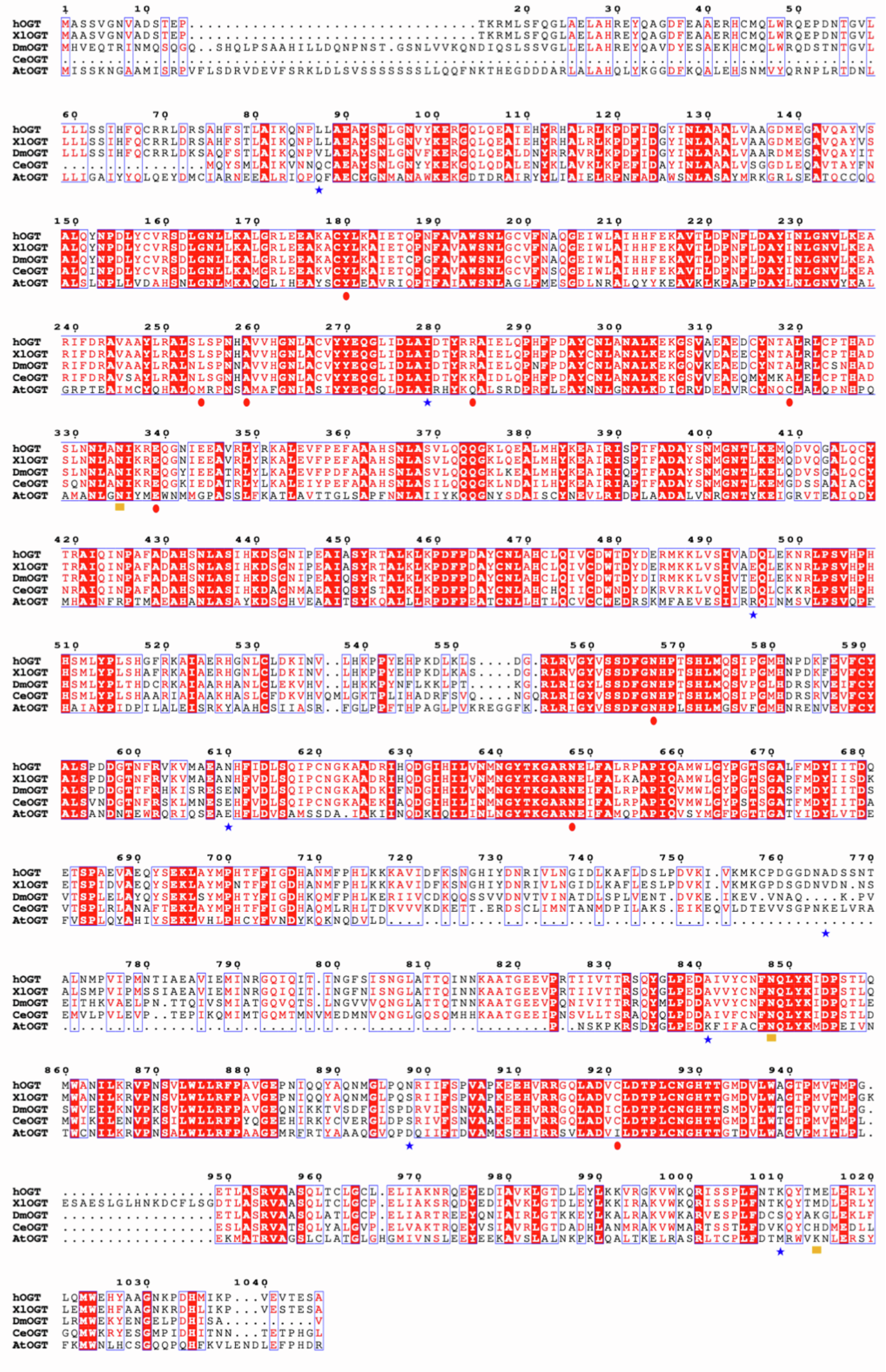

The alignment showcases OGT sequences from various species: human (hOGT, isoform 1), *Xenopus laevis* (*Xl*OGT), *Drosophila melanogaster* (*Dm*OGT), *Caenorhabditis elegans* (*Ce*OGT), and *Arabidopsis thaliana* (*At*OGT). Residues identical across all species are highlighted in red for emphasis, while similar residues are coloured red and enclosed in blue boxes for clarity. Blue star symbols pinpoint the locations of GNOMAD non-pathogenic OGT variants, whereas red solid circles highlight the positions of currently identified pathogenic OGT-CDG variants. Furthermore, orange solid squares denote the locations of newly reported ID-associated OGT variants. Sequence annotation is completed using the online ENDscript server[1].

Figure S4. Representative density plots for mTagBFP2⁺ cell selection, related to Figure 4.

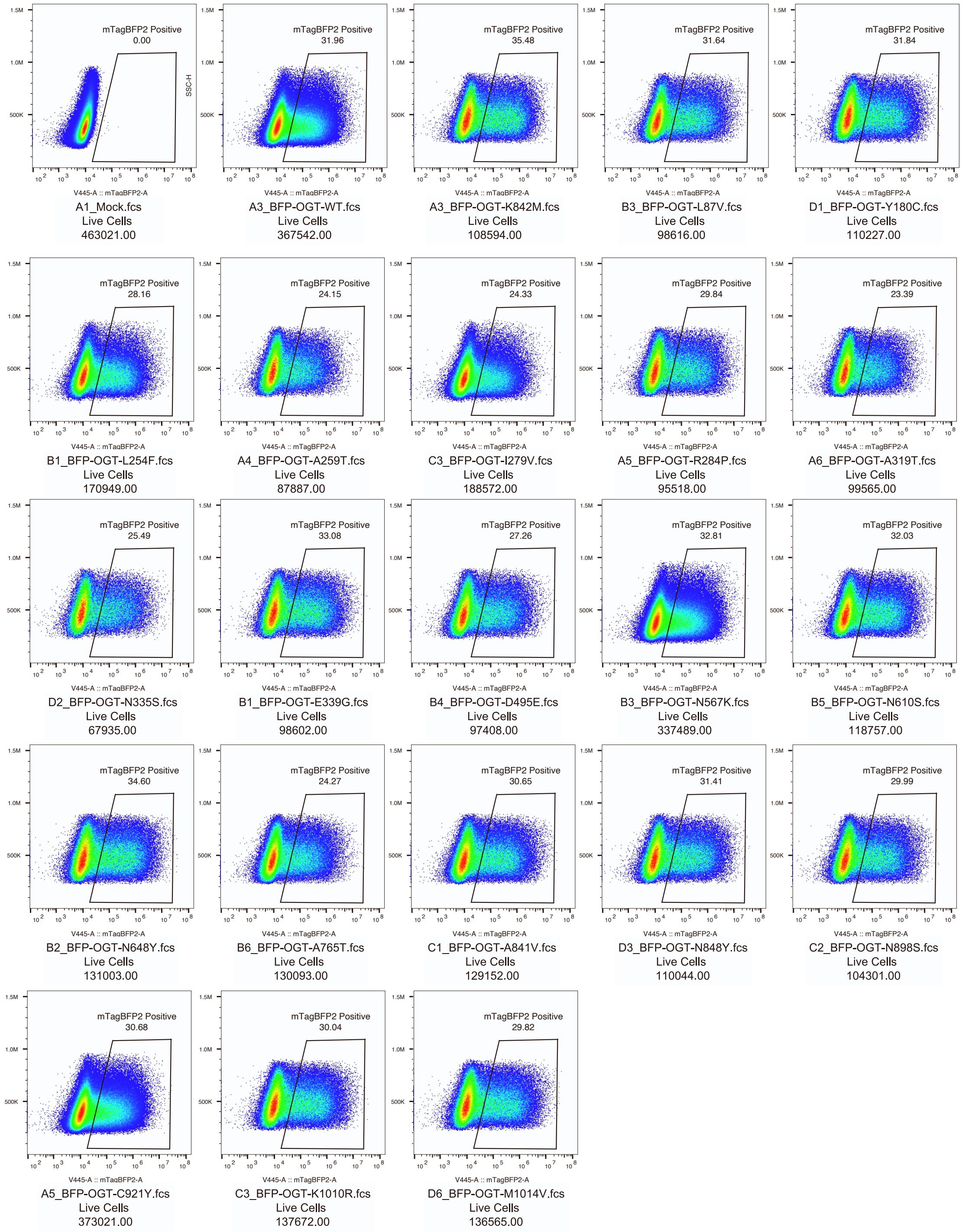

The representative density plots for each type of OGT variant transfected sample are shown, with the specific OGT variant labelled at the bottom of each plot. These plots illustrate the transfection efficiency for each OGT variant and demonstrate the selection of mTagBFP2⁺ cells for further analysis.

Figure S5. The N848Y OGT variant but not the M1014V variant exhibits reduced catalytic efficiency and stability, related to Figure 4.

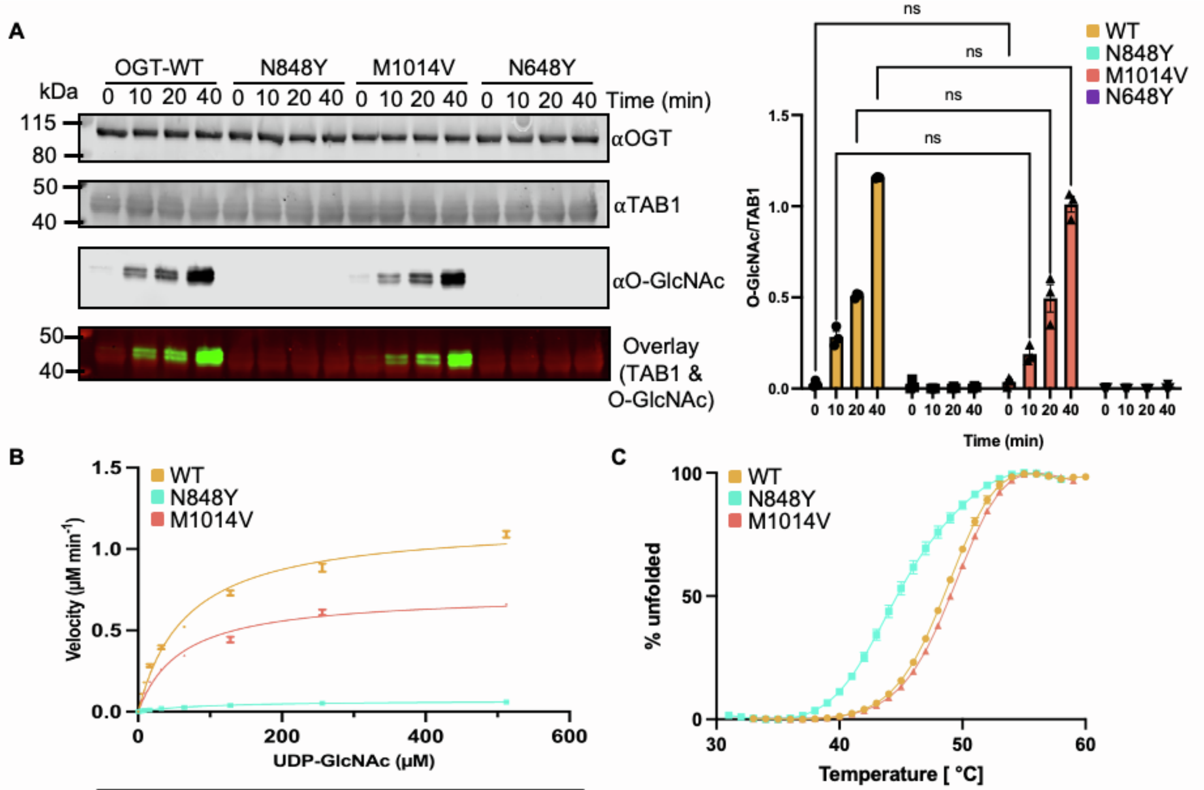

Variant	V_{max} ($nM \cdot s^{-1}$)	K_m (μM)	k_{cat}/K_m ($M^{-1} \cdot s^{-1}$)
WT	1.1 ± 0.033	66 ± 6	0.35
N848Y	0.07 ± 0.002	95 ± 6	0.01
M1014V	0.72 ± 0.020	60 ± 5	0.24

Variant	T_m (°C)
WT	48.37 ± 0.02
N848Y	44.73 ± 0.11
M1014V	48.96 ± 0.03

(A) The N848Y OGT variant but not the M1014V variant showed reduced catalytic activity towards TAB1 *in vitro*. The recombinantly purified wild type OGT or its variants were incubated with TAB1 and UDP-GlcNAc for the indicated time points prior to O-GlcNAc immunoblotting analysis (RL2). Statistical analysis of the immunoblotting data is shown on the right, where an Ordinary One-way ANOVA was performed. The results are presented as the mean O-GlcNAc signal normalized to the TAB1 loading control, with standard error of the mean indicated. The analysis is based on n = 3 independent experiments, with "ns" denoting an adjusted *P* value greater than 0.05.

(B) The N848Y OGT variant but not the M1014V variant presented reduced enzyme kinetics towards TAB1 *in vitro.* The recombinantly purified OGT variants were incubated with excess TAB1 for 40 min during different UDP-GlcNAc concentrations. The reaction was stopped and detected by the UDP-Glo^{TM} kit. Error bars in the graph depict the standard error of the mean, based on three repeated experiments performed on the same day. The kinetics are shown in the table below.

(C) The N848Y OGT variant but not the M1014V variant displayed reduced thermal stability. The recombinantly purified wild type OGT or its variants were analysed in differential scanning fluorimetry. The melting curves and associated T_m values are presented with their standard error of the mean, based on five repeated experiments performed on the same day.

Table S1. Clinical phenotypes of all three probands

OGT variant (reference sequence GenBank: NM_181672.3)	c.1004A>G, p.(N335S)	c.2542A>T, p.(N848Y)	c.3040 A>G, p.(M1014V)
Protein domain	N terminal TPR	Catalytic Region	Catalytic Region
Inheritance	Maternal	*De novo*	
Other genetic variant	-	LMTK3-W464*	LMNA-R545H
Gender	M	F	M
Low birth weight	+	-	+
Hypotonia	+	+	+
Drooling	-	+	-
Developmental delay	+	+	+
Speech and language delay	+	+	+
IQ	WPPSI-III: IQ 79; verbal scale IQ 83; performance scale IQ 81; general speech scale IQ 89	MSEL age 33 moths: Verbal DQ 79, FM 20 months and GM 18 months	Borderline
Epilepsy	-	-	-
Dystonia	-	-	-
Behavioural problems	+	+	-
Brain abnormalities	-	-	-
Eye abnormalities	+	+	-
Ear abnormalities/hearing impairment	-	-	-
Microcephaly	-	+	-
Short stature	-	-	-
Cranio-facial dysmorphisms	+	-	+
Dolicocephaly	-	-	-
Frontal hair upsweep	+	-	
Broad nasal root	-	-	
Full/long philtrum	-	-	
Full lips	+	-	
Non cranio-facial dysmorphisms	+	-	
Clinodactyly	+	-	
Long thin fingers	-	-	
Genital/reproductive abnormalities	-	-	

[a] In the table, a “+” denotes that the patient exhibits the phenotype, while a “−” indicates the absence of the phenotype.

Table S2. Pathogenicity and allele frequency of OGT variants, related to Figures 3-5.

Plasmid Name	Variant Type	Allele Count	Additional information
mTagBFP2-OGT-Y180C	Pathogenic	0	
mTagBFP2-OGT-L254F	Pathogenic	0	
mTagBFP2-OGT-A259T	Pathogenic	0	
mTagBFP2-OGT-R284P	Pathogenic	0	
mTagBFP2-OGT-A319T	Pathogenic	0	
mTagBFP2-OGT-E339G	Pathogenic	0	
mTagBFP2-OGT-N567K	Pathogenic	0	
mTagBFP2-OGT-N648Y	Pathogenic	0	
mTagBFP2-OGT-C921Y	Pathogenic	0	1 additional allele count of C921S
mTagBFP2-OGT-I279V	GNOMAD	115	
mTagBFP2-OGT-E495D	GNOMAD	106	
mTagBFP2-OGT-N610S	GNOMAD	44	
mTagBFP2-OGT-L87V	GNOMAD	29	
mTagBFP2-OGT-K1010R	GNOMAD	29	
mTagBFP2-OGT-A765T	GNOMAD	22	
mTagBFP2-OGT-A841V	GNOMAD	22	
mTagBFP2-OGT-N898S	GNOMAD	21	
mTagBFP2-OGT-N335S	Unknown	0	
mTagBFP2-OGT-N848Y	Unknown	0	
mTagBFP2-OGT-M1014V	Unknown	2	2 additional allele counts of M1014T

The table outlines all types of mTagBFP2-labeled OGT plasmids utilized for transfecting OGT-sfGFP cells. The first column lists the names of the plasmids, each specifying the type of missense mutation present. The second column categorizes the type of each variant: those identified in the literature as causal for ID are labelled ‘Pathogenic’; variants sourced from the general population database, gnomAD[2], are labelled as ‘GNOMAD’; and new ID-associated OGT variants reported in this study are denoted as ‘Unknown’. The third column indicates the allele count of occurrence of the OGT variant from the gnomAD database. The fourth column provides additional information, indicating whether other missense variants at the same residue are reported in the gnomAD database.

Table S3. List of primers used to generate all types of mTagBFP2 labelled OGT plasmid for overexpression in OGT-sfGFP mESCs, related to Figures 3-5.

Plasmid Name	Primers
mTagBFP2-P2A-OGT(K842M)	ctttaatcaactgtacatgatcgacccgtctaccctg, cagggtagacgggtcgatcatgtacagttgattaaag
mTagBFP2-P2A-OGT(Y180C)	gaagcgaaagcctgttgcctgaaagcgattgaaac, gtttcaatcgctttcaggcaacaggctttcgcttc
mTagBFP2-P2A-OGT(L254F)	cctgcgtgcactgagtttttccccgaaccatgccg, cggcatggttcggggaaaaactcagtgcacgcagg
mTagBFP2-P2A-OGT(A259T)	gtccccgaaccataccgtggttcacgg, ccgtgaaccacggtatggttcggggac
mTagBFP2-P2A-OGT(R284P)	gctattgatacctatcgtccggcgattgaactgcagccg, cggctgcagttcaatcgccggacgataggtatcaatagc
mTagBFP2-P2A-OGT(E339G)	ctggcgaacattaagcgtggccaaggcaacattgaagaagc, gcttcttcaatgttgccttggccacgcttaatgttcgccag
mTagBFP2-P2A-OGT(A319T)	gaagattgttataacaccacactgcgtctgtgcccg, cgggcacagacgcagtgtggtgttataacaatcttc
mTagBFP2-P2A-OGT(N567K)	gctctgattttggtaaacatccgaccagccacc, ggtggctggtcggatgtttaccaaaatcagagc
mTagBFP2-P2A-OGT(N648Y)	caccaaaggcgcccgttatgaactgttcgcc, ggcgaacagttcataacgggcgcctttggtg
mTagBFP2-P2A-OGT(C921Y)	cagctggcagacgtgtatctggataccccgctg, cagcggggtatccagatacacgtctgccagctg
mTagBFP2-P2A-OGT(Leu87Val)	gctattaaacaaaatccggtgctggcagaagcttatagc, gctataagcttctgccagcaccggattttgtttaatagc
mTagBFP2-P2A-OGT(Ile279Val)	cctgatcgacctggctgtggatacctatcgtcgcg, cgcgacgataggtatccacagccaggtcgatcagg
mTagBFP2-P2A-OGT(Asp495Glu)	gtctctattgtggccgaacagctggaaaagaacc, ggttcttttccagctgttcggccacaatagagac
mTagBFP2-P2A-OGT(Asn610Ser)	gtgaaagttatggcagaagcttctcacttcatcgatctgtcac, gtgacagatcgatgaagtgagaagcttctgccataactttcac
mTagBFP2-P2A-OGT(Ala765Thr)	gatggcggtgacaacaccgatagttccaacac, gtgttggaactatcggtgttgtcaccgccatc
mTagBFP2-P2A-OGT(A841V)	ggcctgccggaagatgtcattgtgtattgcaac, gttgcaatacacaatgacatcttccggcaggcc
mTagBFP2-P2A-OGT(N898S)	catgggcctgccgcagagtcgtattatcttcag, ctgaagataatacgactctgcggcaggcccatg
mTagBFP2-P2A-OGT(Lys1010Arg)	cgccgctgtttaataccagacagtatacgatggaac, gttccatcgtatactgtctggtattaaacagcggcg
mTagBFP2-P2A-OGT(N335S)	ctgaacaacctggcgagcattaagcgtgaacaag, cttgttcacgcttaatgctcgccaggttgttcag
mTagBFP2-P2A-OGT(N848Y)	gtgtattgcaacttttatcaactgtacaag, cttgtacagttgataaaagttgcaatacac
mTagBFP2-P2A-OGT(M1014V)	gtttaataccaaacagtatacggtggaactggaacgcctg, caggcgttccagttccaccgtatactgtttggtattaaac

Table S4. Sequences of qPCR primers for *Oga*, *Ogt* mRNA measurement, related to Figure 5.

Primer	Sequence (5’ -> 3’)
Actb Forward	AGATCAAGATCATTGCTCCTCCT
Actb Reverse	ACGCAGCTCAGTAACAGTCC
18S Forward	CTCAACACGGGAAACCTCAC
18S Reverse	CGCTCCACCAACTAAGAACG
Ogt Forward	CCCCCTGAGCCCTTCAAAAC
Ogt Reverse	TCGTTGGTTCTGTACTGTCGG
Oga Forward	TGCAGTGGTTAGGGTGTCG
Oga Reverse	AGCAAACGCTGGAACTCTCC

Note S1. Exogenous and Endogenous OGT, related to Figure 5.
Immunoblotting of sorted mTagBFP2$^+$ mESCs with an OGT antibody distinguishes transfected exogenous OGT from endogenous OGT-sfGFP by molecular size, as the sfGFP fusion makes endogenous OGT-sfGFP larger than exogenous transfected OGT.

Note S2. Transfection scale and duration, related to Figures 3-5.
The cell density and the amount of transfection reagents can be scaled up based on the requirements of the subsequent analysis. A DNA to L2K transfection reagent ratio of 1:2 or 1:3 is recommended, as shown in previous experiments[3], to maintain high transfection efficiency while minimizing cytotoxicity. Various transfection durations were tested, with peak exogenous plasmid expression observed at 48 hours post-transfection, which was then chosen for analysing endogenous OGT-sfGFP fluorescence. However, differences in O-GlcNAc feedback regulation between cells transfected with wild type OGT and the OGT-K842M mutant remained evident 72 hours post-transfection.

Supplemental experimental procedures

Ethics and consent
The proband with NM_181672.3: c.1004A>G, p.(N335S) entered the study was approved by the Ethics Committee of IRCCS Burlo Garofolo (Trieste, Italy) with the project number RC10/22, subcentre: L310_22. The proband with NM_181672.2: c.2542A>T, p.(N848Y) was enrolled in the Clinical and Basic Investigations in Congenital Disorders of Glycosylation study at Mayo Clinic in Rochester Minnesota, USA, IRB number 19-005187. The proband with NM_181672.3: c.3040 A>G, p.(M1014V) enrolled in this study was approved by Memorial Healthcare System, Protocol MH # 208.01.

CRISPR/Cas9 reagents and other cloning procedures
The C-terminal sfGFP CRISPR/Cas9 reagents were based on the repair template used for adding a 3xHA tag to mouse OGT[4]. Restrictionless cloning was used to replace the 3xHA tag with sfGFP. The change was confirmed by DNA sequencing. The guide RNA (gRNA) expressing plasmids were used directly as before[4]. Codon-optimised OGT obtained from Genscript was used for exogenous OGT overexpression. The codon bias was originally intended for *E. coli* expression but the ORF has been shown to function in mammalian cells. The sequence was first cloned into pCMV-(c)-HA as a *Bam*HI-*Not*I fragment. This included an upstream CCACC sequence partly coded by the *Bam*HI site. This construct was then cut using *Not*I, dephosphorylated and the BFP sequence was obtained from pBAD-mTagBFP and a P2A sequence was added by the forward primer. The P2A sequence was added by sequential nested PCRs. The final PCR product was cut with *Not*I and *Bsp120i*. This was cloned into the precut vector and this step resulted in the destruction of the *Not*I site at the 3' end of the ORF, but the retention of the *Not*I site between the P2A site and the end of the hOGT ORF. All clones were confirmed with DNA sequencing. Mutations of the hOGT cloned in BP2 were carried out by site-directed mutagenesis based on the Stratagene Quikchange mutagenesis kit, but Kod polymerase was used and *Dpn*I from Thermo Fisher. Sequences of all mutants were confirmed by DNA sequencing. The primer sequences used to generate all human OGT variants for transfection are provided in Table S3.

Codon-optimized *OGT* sequence is provided below, where OGT was shown in lowercase letters, the *Not*I site is shown in uppercase and coloured red, the P2A sequence is shown in uppercase and marked in yellow, and the mTagBFP2 sequence is shown in upper case and coloured blue.
atggcttcaagtgtgggtaatgtggctgattcgacggaaccgaccaaacgtatgctgtcttttcaaggtctggcggaactggctcatcgtgaatat
caggcgggtgattttgaagcggccgaacgtcattgtatgcaactgtggcgccaggaaccggataacaccggcgtgctgctgctgctgagctct
atccattttcagtgccgtcgcctggatcgtagcgcacacttctctacgctggctattaaacaaaatccgctgctggcagaagcttatagcaacctg
ggtaatgtttacaaagaacgcggccaactgcaggaagcgatcgaacattatcgtcacgccctgcgcctgaaaccggattttatcgacggctac
attaacctggcagctgcgctggtcgccgcaggtgatatggaaggcgccgttcaagcatatgtctctgcgctgcagtataacccggacctgtact
gtgtgcgtagtgatctgggtaatctgctgaaagcactgggccgcctggaagaagcgaaagcctgttacctgaaagcgattgaaacccagccg
aactttgcagtcgcttggtctaacctgggttgcgtgttcaatgcacagggcgaaatctggctggctattcatcactttgaaaaagcggtgaccctg
gacccgaatttcctggatgcatatattaacctgggtaatgttctgaaagaagcgcgtatcttcgatcgtgcagtcgctgcatacctgcgtgcactg
agtctgtccccgaaccatgccgtggttcacggtaatctggcatgtgtgtattacgaacagggcctgatcgacctggctattgatacctatcgtcgc
gcgattgaactgcagccgcattttccggatgcctactgcaacctggcgaatgccctgaaagaaaaaggttcggttgcagaagctgaagattgtt
ataacaccgcactgcgtctgtgtccgacccatgctgattccctgaacaacctggcgaacattaagcgtgaacaaggcaacattgaagaagcc
gtccgtctgtatcgtaaagcgctggaagtctttccggaattcgcggcggcacatagtaacctggcctccgtgctgcagcaacagggcaagctg
caggaagctctgatgcactataaagaagcgattcgtatctctccgacctttgccgatgcatacagtaacatgggtaatacgctgaaagaaatgc
aagacgtgcagggcgccctgcaatgttatacccgcgcaattcagatcaacccggctttcgcggatgcccattcaaatctggcatcgattcacaa
agactccggcaacatcccggaagcaattgcttcatatcgtaccgcgctgaagctgaaaccggattttccggacgcttactgcaatctggcgcat
tgtctgcagatcgtctgcgattggacggattatgacgaacgtatgaaaaagctggtctctattgtggccgatcagctggaaaagaaccgcctgc
cgtcagttcatccgcatcactcgatgctgtacccgctgagccatggtttccgtaaagcgatcgccgaacgccacggcaacctgtgcctggataa
gattaatgttctgcataaaccgccatatgaacacccgaaggacctgaaactgagtgatggtcgtctgcgcgttggctacgtcagctctgattttgg
taaccatccgaccagccacctgatgcagtctattccgggcatgcataatccggataagtttgaagtgttctgttatgcgctgtccccggatgacgg
tacgaactttcgtgtgaaagttatggcagaagctaatcacttcatcgatctgtcacagattccgtgcaacggcaaggctgcggaccgcatccatc
aagatggcattcacatcctggttaacatgaatggttacaccaaaggcgcccgtaatgaactgttcgccctgcgtccggcaccgattcaggcaat
gtggctgggttatccgggtaccagcggtgccctgttcatggactacattatcaccgatcaagaaacgtcgccggcagaagtggctgaacagta
tagcgaaaaactggcctacatgccgcatacctttttcatcggtgatcatgctaacatgtttccgcacctgaaaaagaaagcggttattgacttcaa
atcgaatggtcacatctatgataaccgtattgtcctgaatggcatcgacctgaaggcgtttctggacagcctgccggatgtgaaaattgttaagat
gaaatgtccggatggcggtgacaacgccgatagttccaacaccgcactgaatatgccggtgattccgatgaacacgatcgcggaagccgtta
tcgaaatgattaatcgcggccaaattcagatcaccattaacggtttcagcatttctaatggcctggcgaccacgcagatcaacaataaagccgc
aaccggtgaagaagtgccgcgtacgattatcgttaccacgcgctctcagtatggcctgccggaagatgccattgtgtattgcaactttaatcaact

gtacaagatcgacccgtctaccctgcagatgtgggcaaacattctgaaacgtgtcccgaatagtgtgctgtggctgctgcgctttccggccgttg
gtgaaccgaacatccaacagtatgcacaaaacatgggcctgccgcagaatcgtattatcttcagcccggtcgccccgaaagaagaacatgt
gcgtcgcggtcagctggcagacgtgtgtctggataccccgctgtgcaatggtcacaccacgggcatggatgttctgtgggctggtaccccgatg
gtcacgatgccgggcgaaaccctggcaagtcgtgttgctgcctcccagctgacctgcctgggttgtctggaactgattgctaagaaccgccag
gaatatgaagacatcgcggttaaactgggtaccgatctggaatacctgaagaaagttcgtggcaaggtctggaaacaacgcatttcatcgccg
ctgtttaataccaaacagtatacgatggaactggaacgcctgtacctgcaaatgtgggaacactacgcagcaggcaataagccggaccatat
gattaagccggtggaagtgaccgaaagcGCGGCCGCGGGAAGCGGAGCTACTAACTTCAGCCTGCTGAAGC
AGGCTGGAGACGTGGAGGAGAACCCTGGACCTATGAGCGAGCTGATTAAGGAGAACATGCACA
TGAAGCTGTACATGGAGGGCACCGTGGACAACCATCACTTCAAGTGCACATCCGAGGGCGAAG
GCAAGCCCTACGAGGGCACCCAGACCATGAGAATCAAGGTGGTCGAGGGCGGCCCTCTCCCCT
TCGCCTTCGACATCCTGGCTACTAGCTTCCTCTACGGCAGCAAGACCTTCATCAACCACACCCAG
GGCATCCCCGACTTCTTCAAGCAGTCCTTCCCTGAGGGCTTCACATGGGAGAGAGTCACCACAT
ACGAAGACGGGGGCGTGCTGACCGCTACCCAGGACACCAGCCTCCAGGACGGCTGCCTCATCT
ACAACGTCAAGATCAGAGGGGTGAACTTCACATCCAACGGCCCTGTGATGCAGAAGAAAACACT
CGGCTGGGAGGCCTTCACCGAGACGCTGTACCCCGCTGACGGCGGCCTGGAAGGCAGAAACG
ACATGGCCCTGAAGCTCGTGGGCGGGAGCCATCTGATCGCAAACGCCAAGACCACATATAGATC
CAAGAAACCCGCTAAGAACCTCAAGATGCCTGGCGTCTACTATGTGGACTACAGACTGGAAAGA
ATCAAGGAGGCCAACAACGAGACCTACGTCGAGCAGCACGAGGTGGCAGTGGCCAGATACTGC
GACCTCCCTAGCAAACTGGGGCACAAGCTTAATTAA

Cell culture and maintenance

The mESCs used in this study were cultured without feeder cells on 0.1% gelatin-coated plates using GMEM-BHK 21 medium (Gibco, ref 11710035) supplemented with 10% [v/v] fetal bovine serum (Gibco, A5256701), 1 mM sodium pyruvate (Gibco, 11360088), 1000 units/mL LIF (MERK, ESG1107), 0.1 mM MEM nonessential amino acids (Gibco, 11140050), and 0.1 mM 2-mercaptoethanol (Gibco, 31350010) in a 5% CO2 environment at 37 ºC. Cells were maintained in an undifferentiated state without changes in morphology and replication speed and were passaged at a 1:5 ratio. For freezing, cells were preserved using the described GMEM-BHK21 medium supplemented with 10% DMSO. No bacterial contamination was detected throughout the experiment. Cells tested negative for mycoplasma before the experiment began.

To improve transfection efficiency, OGT-sfGFP mESCs cultured in GMEM-BHK 21 medium was switched to 2i medium[5], consisting of 50% DMEM/F-12 with GlutaMAX™ Supplement (Gibco, 31331-093), 50% Neurobasal (Gibco, 21103049), 1X N-2 Supplement (100X) (Gibco, 17502048), and 1X B-27 Supplement (50X, 17504044), supplemented with 1 µM MEK inhibitor PD0325901 (MERK, PZ0162), 3 µM GSK3 inhibitor CHIR99021 (MERK, SML1046), 1000 units/ml LIF (MERK, ESG1107), and 2% [v/v] FBS (Gibco, A5256701). OGT-sfGFP mESCs were cultured in 2i medium for all transfection experiments.

Generation of OGT-sfGFP mES cell line

To tag endogenous OGT with sfGFP, mESCs cultured in GMEM-BHK 21 medium were transfected with Cas9 D10A nickase[6], designed gRNA sequences, and a repair template using Lipofectamine 3000, following the manufacturer's protocol. Details of CRISPR/Cas reagents and cloning procedures are provided above. Post-transfection, the cells underwent puromycin selection at 1 µg/ml for 48 h. The successful tagging was confirmed through restriction enzyme analysis and genomic DNA sequencing. For detailed procedures of the CRISPR/Cas editing, refer to our previously published paper[4].

OSMI-4b and Thiamet G (TMG) treatment

For immunoblotting, 0.5 million OGT-sfGFP mESCs were seeded into 6-well plates and, after 24 hours, treated with 10 µM OSMI-4b or TMG, both at a final concentration of 0.1% DMSO, along with a 0.1% DMSO control and a no-treatment control. After 24 hours of treatment, the cells were harvested for immunoblotting. This experiment was independently repeated three times on different days, each using cells from different passage numbers.

For flow cytometry analysis, 0.1 million OGT-sfGFP mESCs were seeded into 12-well plates. After 24 hours of growth, the cells were treated with 10 µM OSMI-4b or TMG, along with a 0.1% DMSO control. Following 24 hours of treatment, the cells were analysed by flow cytometry (see gating strategy in the No-transfection section of Figure S2). The median sfGFP fluorescence from the sfGFP histogram was used for statistical analysis. This experiment was also independently repeated three

times on different days, with cells from different passage numbers. Stock solutions of OSMI-4b and TMG were prepared in 100% DMSO at a concentration of 10 mM.

Western blot

The harvested cells were washed with PBS and lysed in RIPA buffer (Thermo Fisher). The quantity of extracted crude protein was measured using the PierceTM 660 nm Protein Assay Reagent (Thermo Fisher). 20 µg of crude cell lysate was loaded onto 4-12% Bis-Tris NuPAGE gels (Invitrogen) and transferred to nitrocellulose membranes using a semi-dry transfer system. The transferred membrane was first blocked with 5% BSA [w/v] in TBS-T buffer for at least 1 hour at room temperature. Following this, the membrane was incubated with primary antibodies for the specified times listed below. After primary antibody incubation, the membrane was washed three times with TBS-T buffer, each wash lasting five minutes. The membrane was then incubated with IRDye 680RD (Licor) or IRDye 800CW secondary antibodies for 1 hour at room temperature. Imaging was performed using the Odyssey CLx (Licor) system with 700 nm and 800 nm channels.

The primary antibodies used in this study include OGT (Sigma, DM-17, 1:1000, 2 hours at room temperature), RL2 for O-GlcNAc detection (Thermo Fisher, 1:500, overnight at 4°C), GFP (Proteintech, 66002-1-Ig, 1:5000, 1.5 hours at room temperature), PGK1 (Proteintech, 17811-1-AP, 1:5000, 1.5 hours at room temperature), OGA (Thermo Fisher, SAB4200267, 1:1000, 4 hours at room temperature), and pan-RFP for mTagBFP2 detection (Chromotek, pabr1, 1:5000, 1.5 hours at room temperature).

RT-qPCR analysis

Total RNA was extracted from the sorted mTagBFP2$^+$ cells using the RNAeasy Kit (Qiagen), followed by reverse transcription of 500 ng of RNA using the qScript cDNA Synthesis Kit (Quantabio) according to the manufacturer’s protocol. qPCR was then performed using Perfecta SYBR Green FastMix for iQ (Quantabio), as previously described[4], with *Actb* and 18S serving as internal controls. Each sample was analysed in triplicate, with the mean mRNA abundance considered as one independent replicate. The experiment was repeated three times, with each selected plasmid transfected into OGT-sfGFP cells on different days, using cells of different passage numbers. This provided three independent replicates per plasmid type for statistical analysis. Primers are listed in Table S4.

Transfection

For all transfection experiments, OGT-sfGFP mESCs were cultured in 2i medium[5] to improve transfection efficiency (details of medium composition provided in Cell culture and maintenance section above). The transfection method reported before[3] was used (see also Note S2), where 1.5 µg of DNA plasmids was mixed with 3 µl of Lipofectamine 2000 (L2K) in 100 µl of Opti-MEM according to the manufacture’s protocol. A suspension of 2.5×10^5 cells was incubated with the transfection complex for 5 min before being seeded into 12-well plates. After a 24 h incubation, the cell medium was refreshed, and following an additional 24 h period, cells were harvested for flow cytometry analysis. This transfection procedure applies to all experiments without cell sorting.

OGT-sfGFP mESCs were also transfected with a subset of plasmids, followed by sorting of mTagBFP2$^+$ cells and subsequent RT-qPCR analysis and immunoblotting. For RT-qPCR analysis, 3 µg DNA plasmid was mixed with 6 µl L2K transfection reagent in 250 µl Opti-MEM medium according to the manufacture’s protocol. Then a suspension of 0.6 x 10^6 OGT-sfGFP cells were incubated with the transfection complex for 5 minutes before seeded onto 6-well plate. After a 24 h incubation, the cell medium was refreshed, and following an additional 24 h period, cells were harvested for sorting. The gating strategy followed the same principles presented in Figure S1, with approximately one million mTagBFP2$^+$ cells per plasmid type sorted for RT-qPCR analysis after transfection.

For immunoblotting, 6 µg DNA plasmid was mixed with 18 µl L2K transfection reagent in 600 µl Opti-MEM medium according to the manufacture’s protocol. Then a suspension of 2.0 x 10^6 OGT-sfGFP cells were incubated with the transfection complex for 5 minutes before seeded onto 100 mm petri dish. After a 24 h incubation, the cell medium was refreshed, and following an additional 24 h period, cells were harvested for sorting. The gating strategy followed the same principles presented in Figure S1, with at least two million mTagBFP2$^+$ cells per plasmid type sorted for immunoblotting.

Representative density plots for all types of plasmid transfection are shown in Figure S4.

Flow cytometer configuration
For sample analysis, NovoCyte 3000 flow cytometer equipped with three lasers (405nm, 488nm, and 640nm) and 13 fluorescence detectors (Agilent, Santa Clara, CA) was utilized. mTagBFP2 fluorescence was detected using a 405 nm laser combined with a 445/45 detector. The 488 nm laser, along with a 488/10 detector, was employed for forward scatter (FSC) and side scatter (SSC) measurements. The detection of sfGFP fluorescence was carried out using a 530/30 detector, and PI detection was achieved with a 615/20 detector. Data acquisition was conducted using the NovoExpress package (version 1.6.2, Agilent, Santa Clara, CA) and data analysis was performed using FlowJo™ (version 10). The gating strategy employed is detailed in Figure S2.

For sorting cells, FACSAria III with 4 lasers (405 nm, 488 nm, 561 nm, 633 nm) and 12 fluorescence detectors (BD Biosciences, San Jose, CA) was utilized. mTagBFP2 fluorescence was detected using a 405 nm laser combined with a 450/40 detector. The 488 nm laser, along with a 488/10 detector, was employed for forward scatter (FSC) and side scatter (SSC) measurements, and detection of sfGFP fluorescence was carried out using a 530/30 detector. PI was detected using a 561 nm laser, along with a 610/20 detector. Data acquisition was conducted using the BD FACSDiva Software version 8.0.2 (BD Biosciences, San Jose, CA).

Flow cytometry analysis
Harvested cells were centrifuged at 300 rpm for 3 min, after which the supernatant was discarded. The cell pellet was then resuspended in a non-sticky cell sorting buffer, which comprised PBS supplemented with 1% BSA, 2.5 mM EDTA, and 25 mM HEPES buffer. Propidium iodide (PI) was added to a final concentration of 10 µg/ml. Samples were stored on ice until analysis, with the flow cytometer configuration listed above. OGT-sfGFP mESCs treated with OSMI-4b or TMG were analysed using the No-transfection gating strategy in Figure S2, while all transfected cells were analysed with the Plasmid-transfection gating strategy in Figure S2. Each sample's OGT-sfGFP fluorescence intensity is determined by the median sfGFP fluorescence value from the sfGFP histogram, which is then used for statistical analysis.

Enzyme activity analysis and Differential scanning fluorimetry
In the *in vitro* O-GlcNAcylation assays, purified OGT (323-1044 aa) was incubated at a concentration of 100 nM with 1 µM of the TAB1 substrate (7-420 aa) in the presence of 1 mM UDP-GlcNAc for multiple 10 min intervals before O-GlcNAc Western blot analysis (using RL2 antibody). The pathogenic N648Y OGT-CDG variant[7] served as a negative control. This procedure was independently replicated three times on separate days. For kinetic studies, 50 nM of purified OGT or its variants (323-1044 aa) were incubated with 20 µM TAB1 (7-420 aa) and varying UDP-GlcNAc concentrations, from 512 µM to 0 µM, for 40 min (substrate consumption was less than 10%). Reactions were terminated and analysed using the UDP-Glo™ kit, adhering to the manufacturer's protocol. The experiment was repeated three times on the same day.

For protein thermal stability, purified OGT (323-1014 aa) or its variants were prepared by diluting the protein to a final concentration of 1 mg/mL in a buffer containing 100 mM Tris-HCl, 200 mM NaCl, and 0.5 mM TCEP. This solution was then mixed with SYPRO Orange Protein Gel Stain (Sigma-Aldrich) at a dilution of 1:5000. Subsequently, 25 µL of the assay samples were subjected to testing and analysis as previously described[8].

Supplemental references

1. Robert, X., and Gouet, P. (2014). Deciphering key features in protein structures with the new ENDscript server. Nucleic Acids Res. *42*, W320–W324. https://doi.org/10.1093/nar/gku316.

2. Chen, S., Francioli, L.C., Goodrich, J.K., Collins, R.L., Kanai, M., Wang, Q., Alföldi, J., Watts, N.A., Vittal, C., Gauthier, L.D., et al. (2024). A genomic mutational constraint map using variation in 76,156 human genomes. Nature *625*, 92–100. https://doi.org/10.1038/s41586-023-06045-0.

3. Tamm, C., Kadekar, S., Pijuan-Galitó, S., and Annerén, C. (2016). Fast and Efficient Transfection of Mouse Embryonic Stem Cells Using Non-Viral Reagents. Stem Cell Rev. Rep. *12*, 584–591. https://doi.org/10.1007/s12015-016-9673-5.

4. Pravata, V.M., Muha, V., Gundogdu, M., Ferenbach, A.T., Kakade, P.S., Vandadi, V., Wilmes, A.C., Borodkin, V.S., Joss, S., Stavridis, M.P., et al. (2019). Catalytic deficiency of O-GlcNAc transferase leads to X-linked intellectual disability. Proc. Natl. Acad. Sci. U. S. A. *116*, 14961–14970. https://doi.org/10.1073/pnas.1900065116.

5. Ying, Q.-L., Wray, J., Nichols, J., Batlle-Morera, L., Doble, B., Woodgett, J., Cohen, P., and Smith, A. (2008). The ground state of embryonic stem cell self-renewal. Nature *453*, 519–523. https://doi.org/10.1038/nature06968.

6. Shen, B., Zhang, W., Zhang, J., Zhou, J., Wang, J., Chen, L., Wang, L., Hodgkins, A., Iyer, V., Huang, X., et al. (2014). Efficient genome modification by CRISPR-Cas9 nickase with minimal off-target effects. Nat. Methods *11*, 399–402. https://doi.org/10.1038/nmeth.2857.

7. Pravata, V.M., Gundogdu, M., Bartual, S.G., Ferenbach, A.T., Stavridis, M., Õunap, K., Pajusalu, S., Žordania, R., Wojcik, M.H., and van Aalten, D.M.F. (2020). A missense mutation in the catalytic domain of O-GlcNAc transferase links perturbations in protein O-GlcNAcylation to X-linked intellectual disability. FEBS Lett. *594*, 717–727. https://doi.org/10.1002/1873-3468.13640.

8. Omelková, M., Fenger, C.D., Murray, M., Hammer, T.B., Pravata, V.M., Bartual, S.G., Czajewski, I., Bayat, A., Ferenbach, A.T., Stavridis, M.P., et al. (2023). An *O* -GlcNAc transferase pathogenic variant linked to intellectual disability affects pluripotent stem cell self-renewal. Dis. Model. Mech. *16*, dmm049132. https://doi.org/10.1242/dmm.049132.
